# Data‐Driven Design of Biofunctional Molecularly Imprinted Polymers for Precision Diagnostics

**DOI:** 10.1002/advs.78276

**Published:** 2026-11-09

**Authors:** Nastasia Sanda Moldovean‐Cioroianu, Tamim B. Sarwar, Ekin Sehit, Roland Aydin, Zeynep Altintas

**Affiliations:** ^1^ Bioinspired Materials and Biosensor Technologies Institute of Materials Science Faculty of Engineering Kiel University Kiel Germany; ^2^ Institute of Continuum and Material Mechanics Hamburg University of Technology Hamburg Germany

**Keywords:** bioinspired recognition materials, biosensors, cancer biomarkers detection, ensemble models, in silico design, ML‐driven molecularly imprinted polymers

## Abstract

Conventional imprinted receptors design is laborious and reliant on trial‐and‐error experimentation. To overcome existing challenges, this study integrates molecular mechanics/molecular dynamics (MM/MD) and machine learning (ML) to design molecularly imprinted polymer nanoparticles (nanoMIPs), aiming to uncover synergies and gaps between physics‐informed and data‐driven approaches. By systematically comparing and experimentally evaluating MM/MD formulations with ML‐predicted recipes, we establish a multimodal framework for cross‐validation, method selection, and accelerated development of biomimetic materials for precision cancer diagnostics. A monomer library is screened using hybrid MM/MD method, leading to a generalizable nanoMIP formulation for detecting cancer biomarkers. In parallel, we introduce an ML algorithm that predicts nanoMIP formulations, reducing formulation prediction time for new epitopes from months of conventional computational/experimental screening to seconds after model training. Trained on epitope fragments, the ML framework supports epitope‐mediated imprinting by generating recipes tailored to the physicochemical properties of targeted peptides. The curated dataset and integrated MM/MD‐ML workflow developed in this study, capture molecular interactions beyond experimental reach, while experimental synthesis and evaluation ensure alignment between formulation predictions and nanoMIP performance under experimentally relevant conditions. This multimodal, feedback‐loop‐based framework improves predictive accuracy of imprinted polymer formulations, provides mechanistic insights, and supports hypothesis‐guided design of advanced nanoMIPs.

## Graphical abstract

Integrating molecular mechanics/dynamics (MM/MD) with machine learning (ML) enables data‐driven design of nanoMIPs with exceptional performance in multiplex cancer biomarker detection. Guided by the epitopes’ physicochemical and structural traits, ML‐optimized nanoMIPs enhance their charge complementarity, colloidal stability, and binding specificity. This predictive, transferable framework advances rational nanoMIPs design for diagnostics and next‐generation of adaptive biomimetic materials.

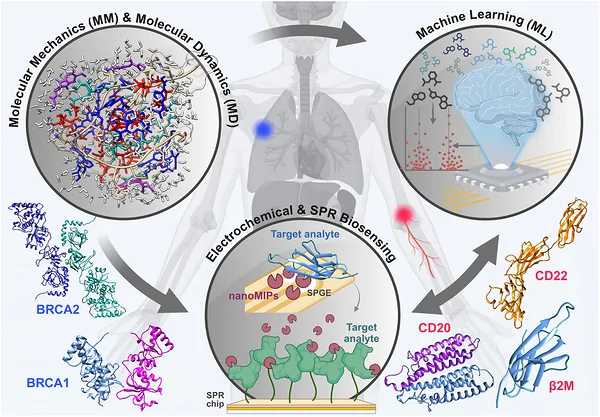

## 1 Introduction

Engineered with cavities tailored for a large array of template (T) molecules, molecularly imprinted polymer nanoparticles (nanoMIPs) excel in recognizing, binding, and retaining the analytes with remarkable selectivity and affinity [1, 2, 3, 4]. Unlike natural receptors, which can be rather unstable, and functional only under limited conditions, nanoMIPs combine the high specificity of natural receptors with the durability, stability and robustness of synthetic materials, retaining selective rebinding, with easy preparation, low cost, and reusability [5–9].

However, their complexity arises from numerous variables including chemical composition related to template (T), functional monomer (FM) and crosslinker (CL) selection, structural dynamics and synthesis conditions, all of which impact morphology, physicochemical properties, and T‐FM‐CL binding capabilities. Additionally, the significant time, resources, and extensive optimization processes demanded by traditional experimental approaches in the development of bioinspired materials present substantial challenges [10–12]. Conversely, in silico methods such as molecular docking, and molecular dynamics (MD), together with machine learning (ML) models can be used to predict both molecular properties and components of bioinspired materials before their actual synthesis, significantly reducing the time and resources required for experiments [13]. Also, scattered and/or incomplete datasets in bioinspired materials field hinder consistent ML data formatting, and limit model learning from high‐quality findings [14, 15]. Moreover, the lack of kinetic data on polymerization and the scarcity of diverse, multi‐domain datasets restrict even further the effective integration of ML in bioinspired materials discovery, design and optimization.

Computational approaches overcome some of the existing challenges in identifying most favorable T:FM:CL complexes for selective and specific nanoMIPs, bypassing conventional and labor‐intensive trial‐and‐errors. In silico methods enable the modeling of host‐guest systems providing predictions often unattainable through experimental means alone [13]. Using empirical scoring functions or molecular mechanics (MM) force fields, molecular docking estimates most stable conformers and receptor‐ligand binding affinities, while MD models explore molecular motions over time in solvated and equilibrated systems, guiding nanoMIPs optimization.

Additionally, epitope‐mediated imprinting offers a practical alternative to whole‐protein imprinting, thereby reducing challenges such as steric hindrance and conformational instability during polymerization. Hence, epitope selection requires careful consideration of multiple structural and physicochemical characteristics, including peptide length, surface exposure, hydropathicity, and secondary‐structure (SS) propensity. However, epitopes that are too short may lack sufficient recognition moieties, whereas longer sequences (i.e. >20 amino acids) can introduce conformational heterogeneity. Therefore, epitopes are selected within a defined size range to properly retain sufficient structural information while remaining practical to synthesize. Moreover, SS propensity represents one of several descriptors associated with epitope rigidity, solvent accessibility, and the exposure of functional moieties. Together with nanoMIP chemical composition, these characteristics are associated with the formation and reproducibility of molecularly imprinted cavities. Within this context, integrating molecular docking‐initialized MD (MM/MD) simulations with experimental data into ML training datasets enables the incorporation of complementary physics‐based structural, dynamic, and physicochemical information beyond that available from sequence descriptors alone. This broader molecular representation allows the ML model to learn relationships between target‐analyte characteristics and nanoMIP components.

Based on this rationale, we developed an integrated computational‐experimental framework for epitope‐mediated nanoMIP formulation design. This study combines three complementary components (Figure 1): (i) MM/MD‐based design of nanoMIP formulations targeting cancer biomarker epitopes; (ii) development of a hybrid MM/MD‐experimental training dataset for an ML model capable of predicting nanoMIP formulation classes, and its deployment on previously unseen epitopes; and (iii) experimental evaluation of MM/MD‐ and ML‐nanoMIP formulations to assess the predictive capability of our trained ML model and the complementary roles of physics‐based and data‐driven approaches in nanoMIP design. The selected biomarker epitopes were deliberately kept separate from ML model development and treated as previously unseen inputs during deployment, enabling an independent experimental evaluation of physics‐based and supervised data‐driven computational design strategies.

**FIGURE 1:**
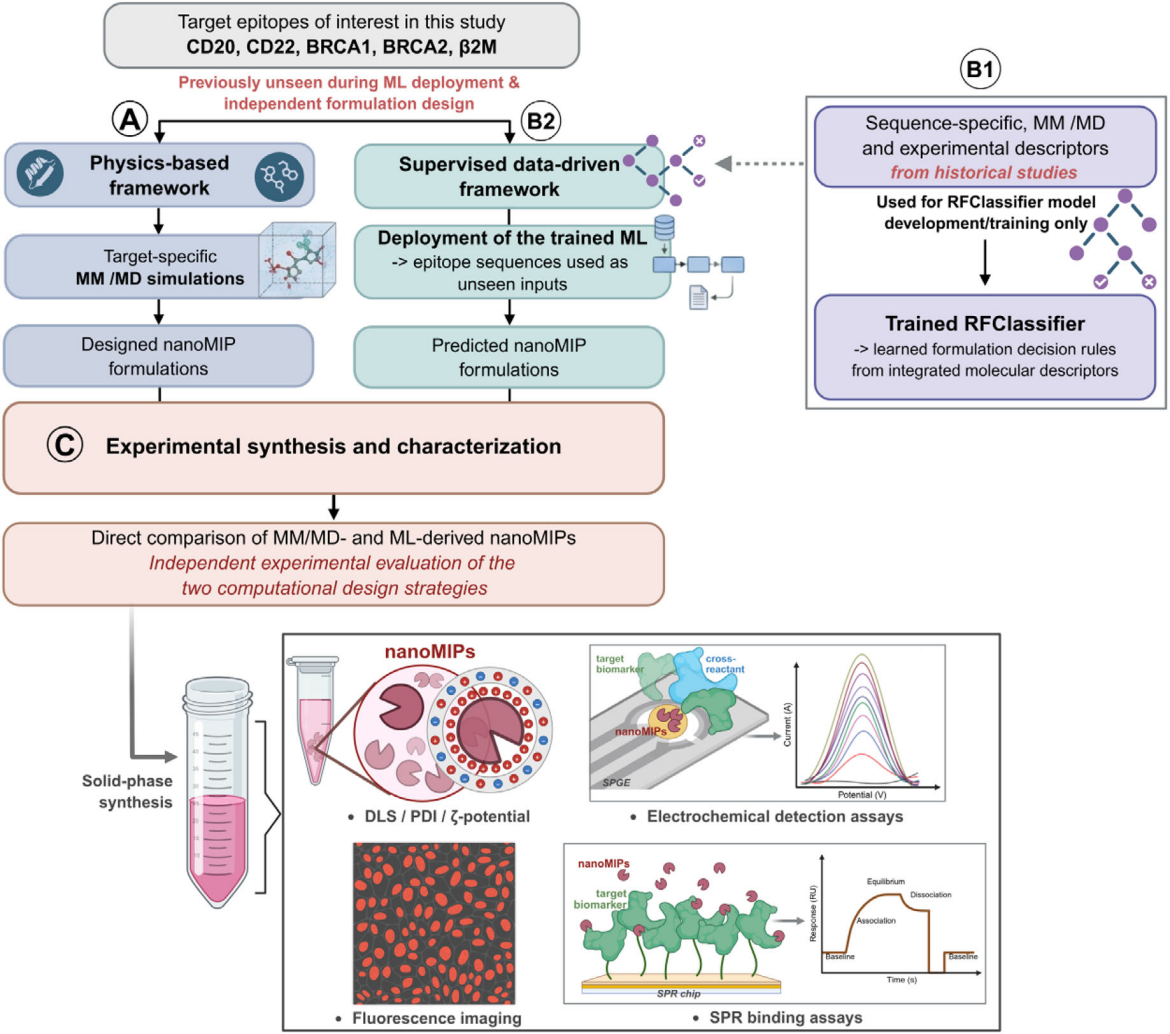
Overview of the integrated computational‐experimental framework and study design for nanoMIP formulation. (A) Physics‐based MM/MD framework used to design target‐specific nanoMIP formulations for the selected epitopes of interest. (B1) Development of the supervised RFClassifier using integrated sequence‐specific, MM/MD, and experimental descriptors derived from historical studies. (B2) Deployment of the trained RFClassifier to predict target‐specific nanoMIP formulations for the same epitopes – treated as previously unseen inputs. (C) Experimental synthesis, physicochemical characterization, and rebinding studies of both MM/MD‐ and ML‐derived nanoMIPs, enabling direct experimental evaluation of the two computational design strategies.

Representative cancer biomarkers used as case studies include: cluster of differentiation 20 and 22 (CD20, CD22), breast cancer type 1 and 2 susceptibility proteins (BRCA1, BRCA2), β2‐microglobulin (β2M), matrix metalloproteinase‐9 (MMP9), and BRAF V600E mutant protein. In all investigated systems, the template (T) molecules correspond to epitope structures (peptides) extracted from whole protein biomarkers. Since isolated epitopes exhibit considerable conformational variability in solvents, secondary structures were assessed in terms of MD trajectory‐based propensities rather than preservation of their native motifs. Epitope selection was guided primarily by root‐mean‐square‐fluctuations (RMSF), solvent‐accessible surface area (SASA), and structural propensities within whole target biomarkers, as indicators of their degrees of flexibility and exposure.

Within the proposed framework, MM/MD and ML models perform complementary rather than interchangeable roles. MM/MD provides the physics‐based molecular descriptors characterizing T‐FM‐CL interactions that, together with experimental measurements, constitute the ML training dataset. The ML model subsequently learns relationships between epitope characteristics and nanoMIP formulation classes, enabling rapid formulation prediction for previously unseen epitopes without requiring additional MM/MD simulations, while remaining within the descriptor space established during training. Accordingly, the nanoMIPs designed using both MM/MD and the trained ML model were produced through solid‐phase synthesis [16, 17], and their quality was defined by their ability to achieve strong and selective rebinding, while maintaining favorable hydrodynamic sizes, low polydispersity index (PDI), and stable ζ‐potential. These experiments assess the feasibility of synthesizing the MM/MD‐ and ML‐based formulations and their measured performance under the investigated conditions. Concurrently, the nanoMIPs' rebinding performance was investigated via square wave voltammetry (SWV) and surface plasmon resonance (SPR) binding assays.

By combining mechanistic modeling with supervised data‐driven prediction, this framework provides a broadly applicable strategy for accelerating epitope‐mediated nanoMIP design across diverse biomolecular targets.

## 2 Results and Discussion

To enable an unbiased experimental assessment of physics‐based and supervised data‐driven nanoMIP formulation strategies, the selected case‐study epitopes were deliberately treated as previously unseen inputs during ML deployment. Consequently, the same epitope sequences were independently subjected to the established MM/MD workflow and trained ML framework, allowing the resulting nanoMIP formulations to be evaluated following experimental synthesis and characterization.

### 2.1 NanoMIPs Design Using a hybrid MM/MD Method

Accordingly, in this study we first designed the physics‐based computational formulations by applying the MM/MD workflow to the selected epitopes (Figure 1A). To identify suitable imprinting targets, epitope candidates were extracted from full‐length cancer biomarker proteins using MD simulations in explicit solvent. Per‐residue RMSF, SASA, and SS propensities were evaluated over production trajectories to assess conformational stability, surface exposure, and structural variability (Figure S1) of the selected epitopes, under predefined MD simulation conditions. Importantly, the calculated SS propensities of epitopes represent the conformational preferences sampled during simulations and are used during ML model training as MD descriptors. They should not be interpreted as evidence that these structural elements are fully retained during the subsequent experimental nanoMIP synthesis.

Prior to molecular docking studies of the T sequences (epitopes) with a library of FM candidates (17 monomers), T sequences were selected based on: (i) surface exposure, to ensure proper non‐bonded interactions with FM candidates, (ii) sequence length of 9–20 amino acids, and (iii) presence of distinct SS propensities (loops, helices, β‐sheets, and combinations thereof), as shown in Table S1 and Figure S1. The biomarkers of interest, and their Protein Data Bank (PDB) [18] identifiers include: CD20 (PDB: 6Y97 [19]), CD22 (PDB: 5VKJ [20]), BRCA1 (PDB: 4IGK [21]), BRCA2 (PDB: 1IYJ [22]), β2M (PDB: 2D4F [23]), MMP9 (PDB: 1L6J [24]), and BRAF‐V600E (PDB: 4MNF [25]).

Each selected epitope was subjected to molecular docking against the 17 FM candidates to identify favorable T‐FM anchoring patterns for subsequent MD refinement. Among all investigated T‐FM docked complexes (Table S2, Figure 2A), 2‐acrylamido‐2‐methyl‐1‐propanesulfonic acid (AMPSA), 2,6‐bis(acrylamido)pyridine (BAAPy), 2‐hydroxyethyl methacrylate (HEMA), and N‐isopropylacrylamide (NIPAM) monomers were selected as best‐performing candidates based on their binding affinity (kcal/mol), RMSD (nm), interatomic minimum distances (Figure 2B) and frequency of T‐FM contacts (Figure 2C). The acidic monomer AMPSA showed consistently favorable docking scores (−2.4 to −4.0 kcal/mol) toward all biomarker investigated epitopes, while BAAPy showed the strongest affinity (−3.0 to −4.2 kcal/mol), particularly with the CD20 and BRAF‐V600 epitopes (Table S2, Figure 2A). These trends reflected the physicochemical characteristics of investigated monomers (Figure 2A), with BAAPy favoring π‐π/hydrophobic contacts and AMPSA favoring H‐bonding and electrostatic contributions. Hot‐spot analysis further identified recurrent T‐FM contacts involving aromatic and hydrophobic residues, including PHE, TRP, LEU, ILE, and VAL (Figure 2C). In contrast, loop‐rich epitopes such as those within MMP9 displayed fewer affinity hot‐spots than more aromatic or charged epitopes such as CD20.

**FIGURE 2:**
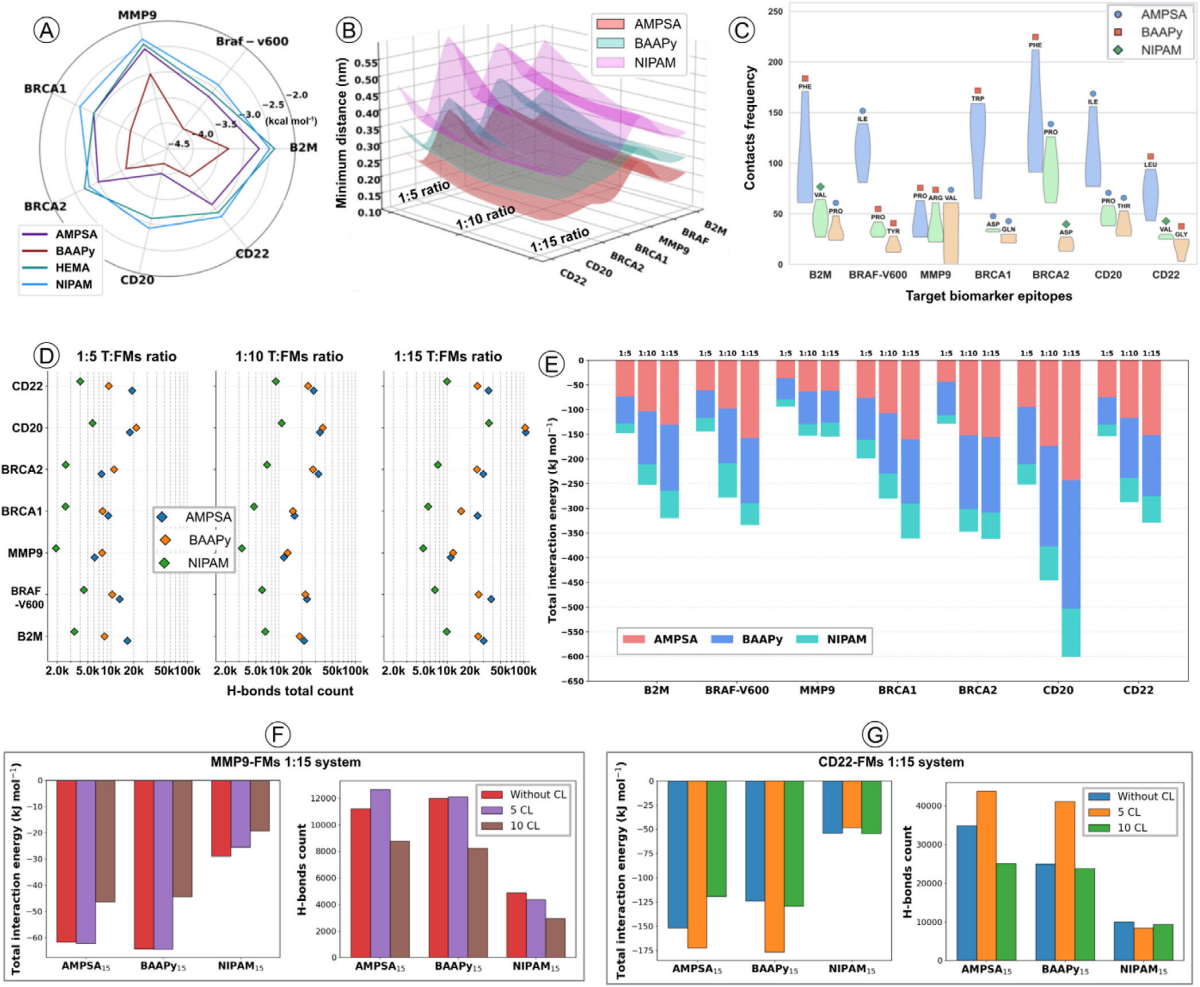
MM‐derived binding affinities for the highest‐scoring T‐FM docked complexes (A), MD‐refined monomer screening reported as average minimum T‐FM distances (B), contact‐frequency analysis between selected FMs and the most populated interacting residues of each biomarker template (C), H‐bond counts for T‐FM interactions across 1:5, 1:10, and 1:15 monomer ratios (D), average total T‐FMs interaction energies (E), and the effect of incorporating additional crosslinkers in the polymerization mixtures for previously under‐performing systems (MMP9 and CD22), highlighting changes in nanoMIP complexation behavior (F‐G).

The top‐ranked T‐FM docked complexes were subsequently refined by 200 ns MD productions, and analyzed considering the following metrics: (i) the average interatomic minimum distance (MinDist) between each FM and its corresponding T (Figure 2B); (ii) the distribution of intermolecular H‐bonds (Figure 2D); and (iii) the total T‐FMs interaction energies, defined as the sum of short‐range Lennard‐Jones and Coulomb potentials (Figure 2E).

An initial low‐ratio MM/MD screening (Table S3) identified AMPSA, BAAPy, and NIPAM as the most promising monomer candidates, which were subsequently evaluated at increasing T:FM ratios of 1:5, 1:10, and 1:15. Expectedly, increasing the T:FM ratio generally strengthened T‐FM interactions, as reflected by shorter interatomic minimum distances, higher total intermolecular H‐bond counts, and more favorable total interaction energies (Figure 2B,D,E; Tables S4 and S5). AMPSA consistently formed the highest number of H‐bonds across epitopes, whereas BAAPy exhibited strong interactions with selected epitopes. Although NIPAM displayed larger interatomic distances with epitopes at lower ratios, its performance improved at higher concentrations (e.g., from 0.258 nm at 1:5 to 0.203 nm at 1:15 in CD20 systems). The most pronounced ratio‐dependent enhancement was observed for CD20‐ and β2M‐based systems. For instance, for CD20‐FM complexes, from 1:5 to 1:15 ratio, interatomic T‐FM distances decreased from 0.235‐0.258 nm (1:5) to 0.189‐0.203 nm (1:15), H‐bond total counts increased from 5,782‐21,542 (1:5) to 35,313‐107,072 (1:15), and interaction energies strengthened from −41.460 to −115.957 kJ mol^−^^1^ (1:5), and from −97.410 to −260.598 kJ mol^−^^1^ (1:15). By contrast, MMP9‐based systems showed little to no improvement beyond 1:10 ratio, with total H‐bond counts decreasing for AMPSA and BAAPy from 11,634 and 13,019 to 11,211 and 11,998, respectively, and their interaction energies becoming slightly less favorable. As another example, in BRAF‐V600 systems, from 1:10 to 1:15 ratio NIPAM became substantially less favorable energetically, changing from −69.136 kJ mol^−^^1^ to −44.214 kJ mol^−^^1^, despite modest improvements in interatomic distances and H‐bonds count (Table S5). Moreover, for BRCA2 systems, total T‐FM H‐bonds count decreases with the increasing concentration of both AMPSA and BAAPy monomers in the mixture. These contrasting instances indicate that while increasing FM concentration can indeed enhance interaction density for some epitopes, such as CD20 and β2M (Tables S4 and S5), it may also provide diminishing effects once the epitope‐specific interaction environment becomes saturated or geometrically constrained, as observed for MMP9. In other words, increasing FM concentration alone may not always compensate for unfavorable epitope conformations, such as trappings within highly flexible loops/coils or mismatched secondary structures. Rather, both monomer composition and stoichiometry must be tailored to the molecular recognition landscape of each target epitope.

Crosslinker addition was tested only for the weakest‐performing MD systems, MMP9 and CD22, at the 1:15 T:FMs ratio, by adding 5 vs 10 N,N′‐methylenebisacrylamide (BIS) molecules (Figure 2F,G). As a result, crosslinking behavior showed divergent effects: in CD22 systems, low BIS concentration (1:5) moderately enhanced T‐FM interactions, while higher concentration had detrimental effects (Table S6). Within MMP9 systems, T‐FM interactions weakened upon BIS addition (Table S7) in both ratios. Thus, the effect of crosslinking content represents a formulation‐specific optimization parameter rather than a universally transferable design variable.

The selected MM/MD nanoMIP recipes including AMPSA‐BAAPy‐NIPAM monomers for CD20 (1:15), CD22 (1:15, with 5 BIS molecules), BRCA1 (1:15), BRCA2 (1:10), and β2M (1:15) target epitopes, are highlighted in Table S5. These formulations were synthesized via solid‐phase method [16, 17] and characterized using physicochemical analyses, including dynamic light scattering (DLS), polydispersity index (PDI), ζ‐potential, as well as affinity measurements (epitope/protein electrochemical assays and SPR), and cross‐reactivity tests (electrochemical studies).

Collectively, the MM/MD analyses show that nanoMIP formulation is based on a complex chemical interplay between monomer composition, stoichiometry, and crosslinker content, with their relative contributions varying across epitopes. These observations are inherently bounded by the molecular systems and simulation protocols employed, in which alternative formulations, including systems with and without BIS, were investigated through independent MM/MD simulations. Moreover, the MM/MD trends should be interpreted as physics‐derived mechanistic insights and formulation design principles for the pre‐polymerization molecular recognition stage, rather than representing the polymerization process itself. Separately, historical physics‐derived descriptors were used as an independent benchmarking strategy, and incorporated into the ML training because they capture molecular‐recognition features that cannot be inferred directly from sequence alone, enabling the Random Forest (RF) to learn descriptor‐formulation relationships grounded in both historical physicochemical interactions and experimental observations. The selected MM/MD formulations were therefore used for independent experimental evaluation and comparison with the formulations predicted by our ML framework, deployed on the same target epitopes. This parallel experimental evaluation remains essential because neither the MM/MD nor the ML framework predicts nanoMIP performance; rather, both independently generate candidate nanoMIP formulations whose physicochemical properties and binding behavior must ultimately be verified experimentally.

### 2.2 ML Algorithm Development and Features Relevance

Concurrently with the MM/MD studies, the supervised ML framework was developed to transition the complex MM/MD‐experiment relationships into a platform for rapid nanoMIP formulation class prediction. Unlike the deterministic, physics‐based MM/MD workflow, our ML framework learns descriptor‐formulation class relationships from historical MM/MD, experimental, and (known) epitope sequence‐derived descriptors. The epitopes of interest (CD20, CD22, BRCA1, BRCA2, and β2M) were deliberately excluded from model development and treated as previously unseen during ML deployment (Figure 3). The training dataset comprises 37 numerical and categorical descriptors derived from epitope sequences, MM/MD simulations, and experimentally characterized nanoMIPs (Figure 3A, Tables S10 and S11). Additional dataset‐related details are provided in the SI.

**FIGURE 3:**
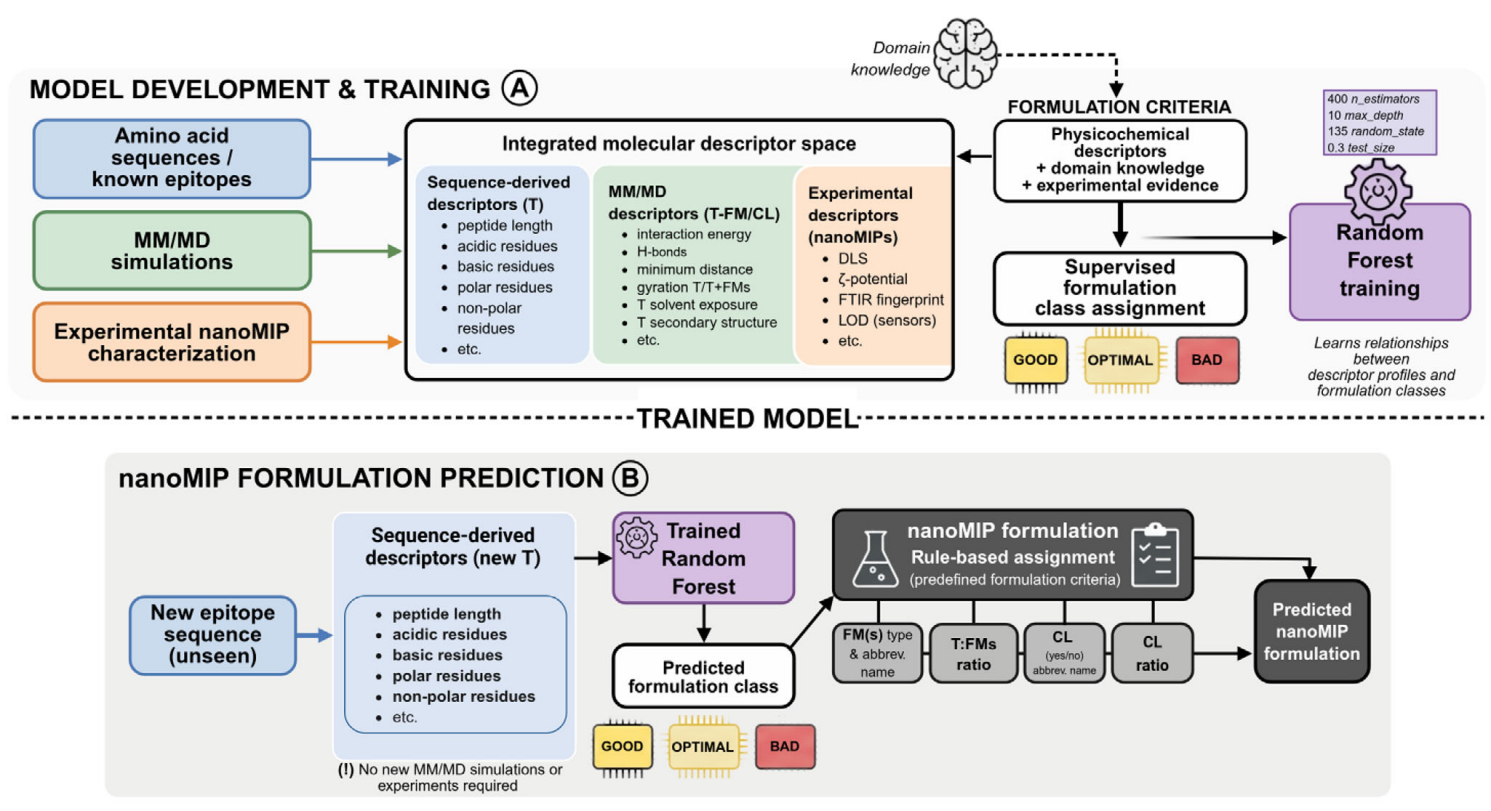
Workflow of the ML framework for nanoMIP formulation prediction. (A) Model development and training. Historical (“known”) epitopes are represented by an integrated molecular descriptor space comprising historical sequence‐derived features, MM/MD data and experimentally characterized nanoMIP properties. Based on predefined formulation criteria established from these physicochemical profiles, experimental evidence, and domain knowledge, each historical formulation is assigned to one of three supervisory formulation classes (“Good”, “Optimal”, or “Bad”). The RFClassifier is then trained to learn the relationships between the descriptor space and the corresponding formulation classes. (B) Model deployment. A previously unseen epitope is represented only by sequence‐derived features, which are processed by the trained RFClassifier to predict the most appropriate formulation class. The predicted formulation class is subsequently used to construct a target‐specific nanoMIP formulation by applying the predefined formulation criteria. No additional MM/MD simulations or experimental characterization are required during deployment.

During model development (Figure 3A), the integrated descriptor space was associated with three supervisory MIP formulation classes (labeled as “Bad”, “Good”, and “Optimal”) using predefined criteria derived from MM/MD descriptors, experimental evidence, and domain knowledge. Consequently, each formulation class represents a multidimensional physicochemical state defined by numerous correlated molecular, structural, energetic, and experimental descriptors rather than by any individual feature. The RF classifier was then trained to learn the relationships between these descriptors and the corresponding formulation classes. During deployment (Figure 3B), previously unseen epitope sequences are represented only by sequence‐derived features, which are processed by the trained RF to predict the formulation class (direct RF output). This predicted class is subsequently used to construct the target‐specific nanoMIP formulation by applying the same predefined criteria established during development (Figure 3A), thereby determining the FM types, T:FMs ratio, crosslinking requirement and ratio (Figure 3B). This strategy combines data‐driven prediction with chemically informed formulation rules. Moreover, experimental and MM/MD descriptors are required only during model development and training to establish and learn the descriptor‐formulation class relationships, therefore are not needed during deployment.

We employed the RF classifier model [26–28] due to its ability to handle heterogeneous datasets, it is less prone to overfitting, and provides interpretable feature importance rankings, aligning well with the study's objective of identifying the descriptors most relevant for nanoMIP formulation prediction. The scalability and efficiency of RF further supported its use, ensuring reliable predictions while remaining computationally feasible. For this purpose, we used a pipeline with *StandardScaler* for feature scaling and *GridSearchCV* for hyperparameter tuning (Figure 4A).

**FIGURE 4:**
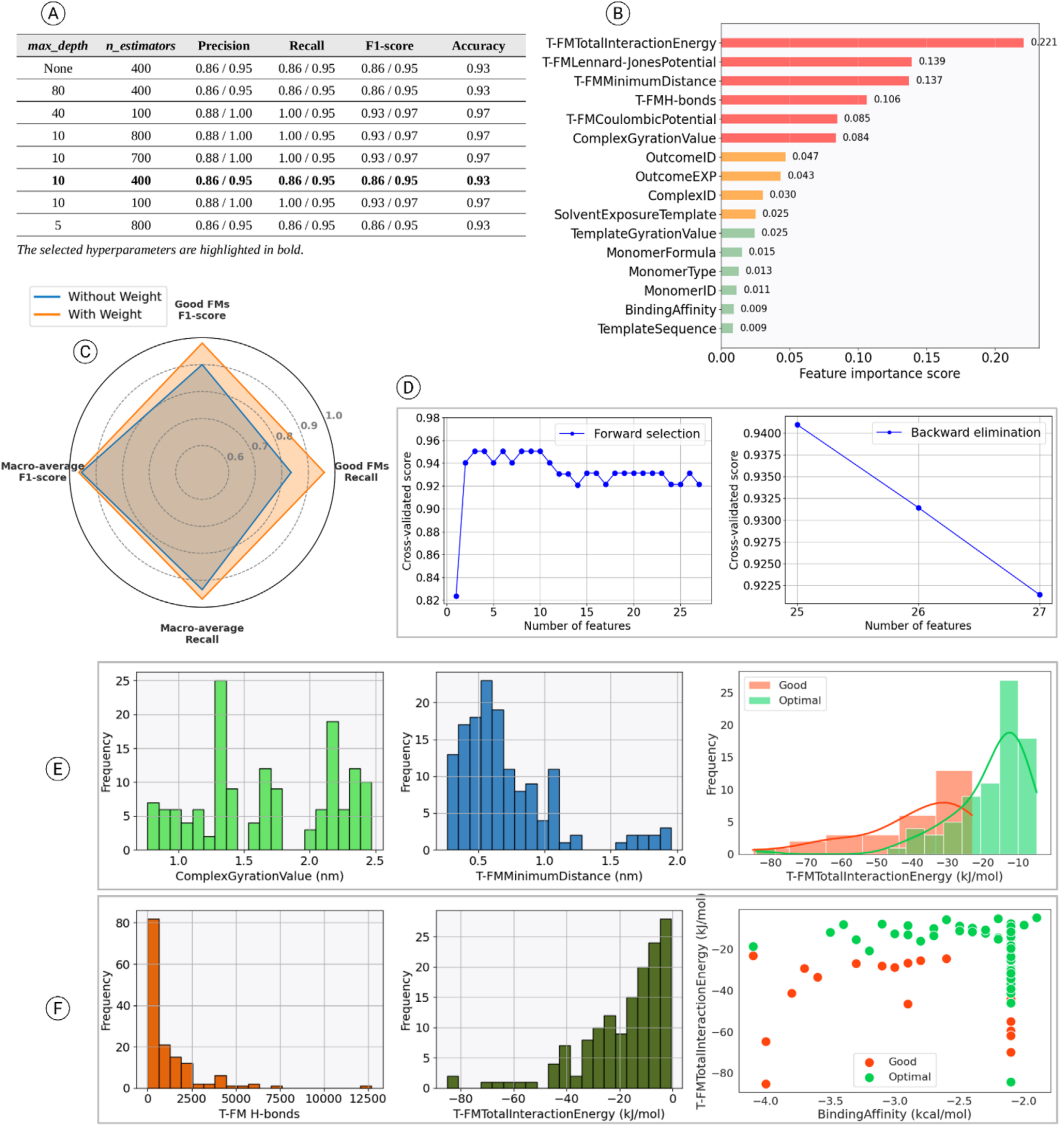
Hyperparameters tuning of RF classifier with ‘good’/‘optimal’ MIP formulation classes prediction (A), feature relevance analysis (B) for both numerical and categorical data, comparison of recall, F1‐score and macro‐averages for ‘Good’ MIP components in both weight‐related scenarios (C), sequential forward features selection and backward feature elimination (D), as well as data distribution of the most relevant features used for selection criteria refinement (E, F).

Upon testing multiple hyperparameter combinations, the optimized RF employed 400 estimators and a maximum tree depth of 10. Model training used a test size of 0.3 with a random state of 135, providing the best balance between predictive performance and model generalization (Figures 3A and S2).

In terms of feature relevance, the conducted studies evaluated mean decrease in impurity importance, permutation analysis, and SHapley Additive exPlanations (SHAP) values. The model prioritizes energy components (Lennard‐Jones, Coulomb), H‐bonds, and distance‐related properties (Figure 4B), while categorical features, although less dominant than the numerical ones, also contribute to revealing patterns.

The focus was also oriented toward exploring which combinations of features have the highest impact on the model's predictions. Recursive Feature Elimination (RFE) was used on the unweighted dataset to systematically identify and rank the most critical features contributing to nanoMIP rule‐based formulation prediction, enabling efficient dimensionality reduction without loss of accuracy (Figure 4D; SI for details). The classification metrics for nanoMIP components labeled as ‘Bad’ remained consistent across RFE scenarios, with stable precision and recall regardless of the feature subset size. Separately, a dedicated feature‐weighting analysis revealed that feature weights improved precision, recall, and F1‐scores for classes labeled as ‘Good,’ enhancing the model's ability to identify positive cases (Figure 4C). Overall, the model achieved 95% accuracy in identifying favorable instances with feature‐assigned weights.

Finally, to properly account for selection criteria values, we conducted a distribution analysis (Figure 4E,F) on the datapoints describing the most relevant features (detailed in SI). Outliers were assessed, and any missing data were imputed via median values. Moreover, the rationale behind the T‐FM ratios and crosslinking integration, as well as structural, dynamical and physicochemical correlations behind the formulation classes predictions, are thoroughly depicted in the SI.

The trained ML framework was subsequently deployed to predict nanoMIP formulations for the previously unseen study epitopes (CD20, CD22, BRCA1, BRCA2, and β2M), enabling an independent experimental evaluation alongside the MM/MD‐derived formulations.

### 2.3 MM/MD vs ML nanoMIP Recipes for Cancer Biomarkers Detection

The secondary structure (SS) descriptors discussed throughout the following sections correspond to structural propensities predicted by our MD simulations during epitope selection studies, rather than experimentally determined structural components. These computational descriptors form part of the integrated descriptor space for ML‐based formulation classes prediction, to capture the epitopes structural preferences that may influence T‐FM associations.

The ML deployment on the same epitope sequences, considered unseen, prompted the same FM types (AMPSA, BAAPy, and NIPAM), as the ones derived from MM/MD studies, across most of the targeted sequences (CD20, BRCA1, BRCA2), while changing only the FMs concentrations. However, for CD22 and β2M nanoMIPs the ML model also changed the FMs type (Table S8). For CD22 nanoMIPs, it identified N‐(3‐aminopropyl) methacrylamide (APMA; instead of BAAPy) as the preferred basic monomer and N,O‐bismethacryloyl ethanolamine (NOBE; instead of NIPAM) as the neutral one. For β2M nanoMIPs, 2‐(methacryloyloxy)ethyl phosphate (MEP; instead of AMPSA) was predicted as the best acidic candidate, with APMA (instead of BAAPy) again favored as the basic monomer.

The differences between the MM/MD‐guided and ML‐predicted nanoMIP formulations may be associated with the increased β‐sheet propensities predicted for the CD22 and β2M epitopes using MD simulations (Tables S1, S8). These β‐sheet‐like conformations are characterized by extended backbone geometries and directional H‐bonding networks, often exposing alternating polar and non‐polar side chains on opposite sides of the strand [29]. Although similar effects may occur in other structural motifs, mechanistically speaking, increased β‐sheet propensities may reduce the likelihood of forming complementary imprinting cavities by limiting favorable T‐FM interactions during FMs assembly.

The substitution of MEP monomer for AMPSA may be attributed to the distinction between phosphate (MEP) vs sulfonate (AMPSA) interactions. Although less acidic than AMPSA, MEP's phosphate group can form diverse H‐bonds and salt bridges, potentially offering more adaptable binding behavior for the targeted epitope. Moreover, the substitution of NIPAM by NOBE may reflect differences in the balance between H‐bonding capability and overall physicochemical compatibility with the predicted formulation class. Also, unlike NIPAM, NOBE is a bifunctional methacrylate that can participate in polymer network stabilization while simultaneously providing polar interaction sites. This additional structural role may contribute to the physicochemical profile identified by our ML framework as favorable for the epitope. Lastly, APMA – exchanged from BAAPy, has a primary amine group indicating its potential to strongly interact with negatively charged and/or polar residues via electrostatics and stable H‐bonds. Additionally, BAAPy monomer lacks the flexible side chain of APMA, being more rigid due to the presence of an aromatic ring. Nevertheless, the precise molecular traits behind these monomer substitutions remain difficult to isolate, because our ML framework evaluates multiple correlated descriptors simultaneously. Hence, the ML‐based nanoMIP formulations should be interpreted as reflecting descriptor patterns learned from historical nanoMIP formulations, rather than the influence of a single molecular/chemical property.

### 2.4 NanoMIP Clustering, Pair‐Wise Analyses and Descriptor Correlations

To experimentally evaluate the formulations proposed by our ML framework, nanoMIPs corresponding to the five MM/MD formulations and their five ML‐predicted counterparts (Table S8) were synthesized using the solid‐phase method. The resulting nanoMIPs were characterized in terms of hydrodynamic size, PDI, ζ‐potential, and epitope rebinding performance (in PBS). Rebinding profiles were assessed via electrochemical assays, and the resulting experimental data were fitted to the Langmuir (L), Freundlich (F), and Langmuir‐Freundlich (L‐F) binding isotherms. The corresponding heterogeneity indices (m) were extracted from the L‐F fitting. These physicochemical descriptors (hydrodynamic size, PDI, and ζ‐potential) and binding descriptors (Kd, m, and R^2^ values obtained from the isotherm and linear fittings) were subsequently used for the t‐SNE and pair‐wise analyses (Figure 5).

**FIGURE 5:**
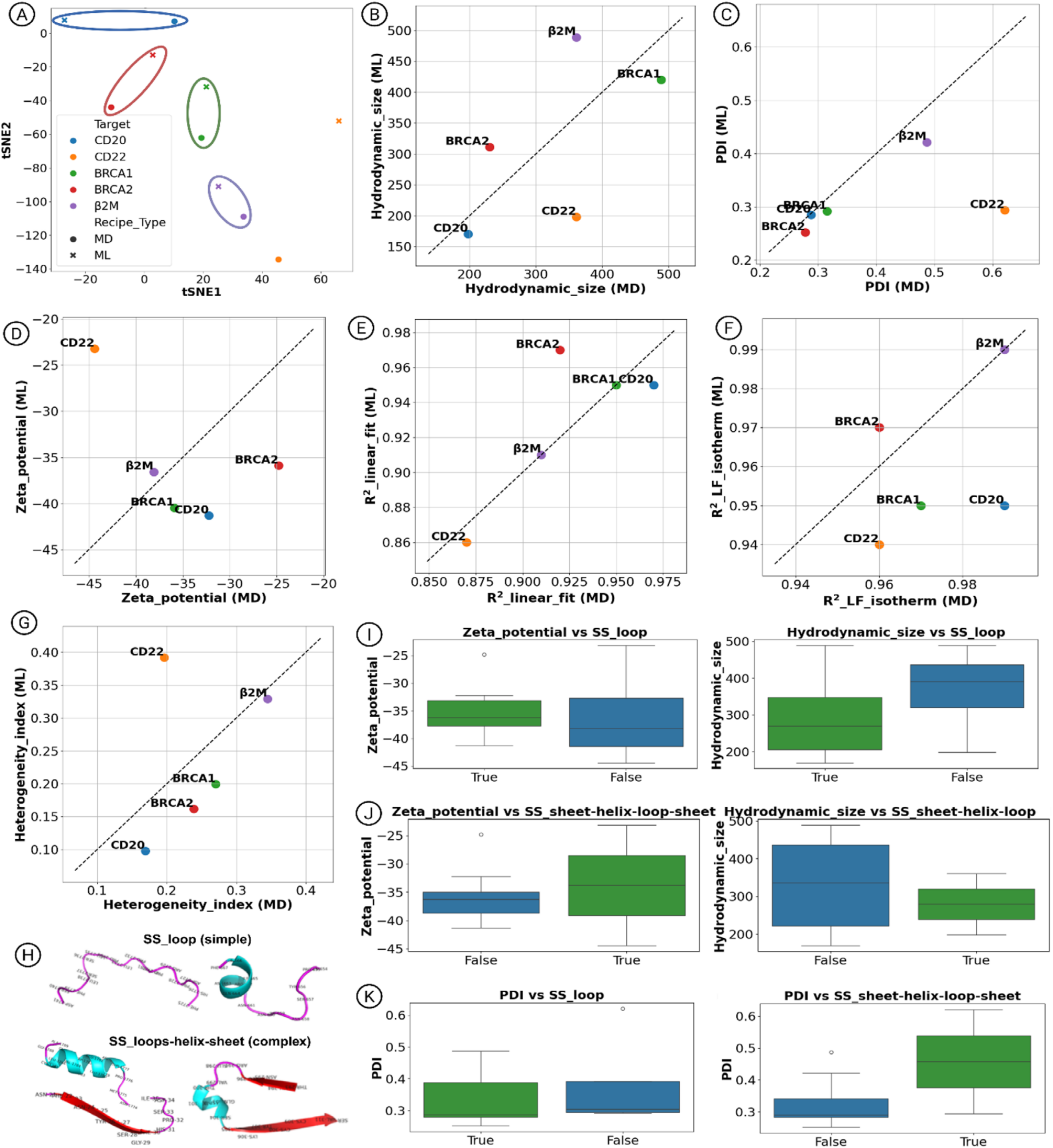
Cluster and pair‐wise analysis comparing MM/MD vs ML nanoMIP formulations. The t‐SNE embedding integrates amino acid sequence descriptors of the T molecules with normalized z‐scores of nanoMIP performance (A), offering a global perspective on how targeted sequence patterns align with functional nanoMIP outcomes. This analysis was performed on a dataset of 10 samples, each represented by a set of aggregated performance descriptors: hydrodynamic size, PDI, ζ‐potential, linear R^2^, isotherm fittings, Kd values, and heterogeneity indices. Complementary pair‐wise comparisons across MM/MD and ML nanoMIP formulations reveal specific trends in hydrodynamic sizes, polydispersity index, and ζ‐potential (B‐D), together with consistent peptide rebinding metrics captured through linear‐fit R^2^ values, Langmuir‐Freundlich isotherm fits, and corresponding heterogeneity indices (E‐G). The MD‐predicted structural propensities (H) provide qualitative context for interpreting the experimentally observed nanoMIP profiles. Boxplot analyses compare nanoMIPs associated with predominantly loop‐like structural propensities (SS_loop) and mixed structural propensities (SS_sheet‐helix‐loop‐sheet) predicted by the MM/MD simulations (I‐K). The t‐SNE with perplexity of 3 was applied to explore local similarities within data. The comparisons between MM/MD‐ and ML nanoMIPs are intended to identify trends rather than establish causal relationships. Each point (B‐G) represents a matched pair for a given target. The dashed line indicates perfect agreement (y = x). For SS propensity involvement (I‐K), samples were grouped based on the presence or absence of specific structural motifs. For each group, distributions of ζ‐potential, hydrodynamic size, and polydispersity index (PDI) were compared. Due to the limited sample size, these analyses are intended solely to explore trends and potential associations, rather than establish definitive statistics; therefore, no formal statistical tests were applied.

Considering the data size, t‐SNE analysis is intended solely for qualitative exploration of data rather than quantitative analysis. Accordingly, the clustering patterns should be interpreted together with the experimental physicochemical and binding measurements. Results reveal distinct experimental trends across the investigated formulations. MM/MD‐based CD22 nanoMIPs showed the highest PDI and the lowest linear R^2^ values among all samples, whereas the corresponding ML‐based formulation substantially reduced both the hydrodynamic size and PDI of the synthesized CD22 nanoMIPs. Similarly, the ML formulations for CD20, BRCA1, and BRCA2 tend to improve particle quality (Table S8) together with comparable or improved epitope rebinding performance relative to their MM/MD counterparts.

Pair‐wise analyses of the physicochemical and binding descriptors for both formulation strategies are summarized in Figure 5B–G. Overall, the ML‐based recipes showed smaller hydrodynamic sizes compared to MD formulations, particularly for the CD22 epitope, which exhibited higher MD‐predicted β‐sheet structural propensity. In this case, the particle size decreased from 361 nm (MM/MD) to 198 nm (ML), corresponding to a 45% reduction. Following similar patterns, although less pronounced, CD20 and BRCA1 nanoMIP sizes showed a size reduction of ∼14% for the ML recipes. The ML formulations also reduced particle polydispersity, most notably for CD22, where the PDI decreased by approximately 54% (Table S8), while CD20 and BRCA nanoMIPs maintained similarly low PDI values across both formulation strategies (Figure 5C). Collectively, these findings (Figure 5A–G) suggest the epitope sequence‐dependent nature of nanoMIP formulation and indicate that our ML framework can identify alternative formulations yielding improved physicochemical characteristics and binding behavior, even for epitopes exhibiting more complex MD‐predicted structural propensities.

Moreover, ML formulations show higher ζ‐potentials (in magnitude) for the BRCA1, BRCA2, and CD20 nanoMIPs, indicating improved colloidal stability (Figure 5D). However, CD22 and β2M showed the opposite trend, reflecting the changes in monomer composition introduced by our ML framework. For CD22, the more negative ζ‐potential of the MM/MD formulation (‐44.4 mV vs. ‐23.2 mV for ML) may be associated with the higher proportion of the strongly anionic AMPSA monomer (1:15). For β2M nanoMIPs, the smaller ζ‐potential differences may arise from more balanced contributions of the acidic (MEP) and basic (APMA) monomers within the ML formulation.

Figure 5E–G shows the pair‐wise trends with respect to the nanoMIPs' capability to rebind the targeted epitopes. Linear fitting of the electrochemical responses (for MM/MD‐ML formulations) indicated R^2^ values of 0.92–0.97 for the BRCA and CD20 nanoMIPs, 0.86–0.87 for CD22, and 0.91 for both β2M formulations. Moreover, the L‐F fittings show R^2^ values between 0.94–0.99 across all nanoMIPs (Figure 5F). The corresponding heterogeneity indices (*m*) remained largely comparable between the MM/MD‐ and ML‐based formulations, with CD22 representing the main exception (Figure 5G). For reference, *m* values closer to 1 indicate more homogeneous binding sites, while values closer to 0 are reported as heterogeneous [30]. MD‐based CD22 nanoMIPs show more heterogeneity (*m* = 0.1965), while the ML ones indicate more homogeneous cavities (*m* = 0.3921). This may be attributed to the substitution of NIPAM by the bifunctional monomer NOBE within the ML formulation; however, this finding should be interpreted in the context of the complete formulation rather than a single compositional change. Notably, the objective of comparing these findings is not necessarily to indicate that ML‐derived formulations systematically outperform the MM/MD ones. Rather, in this study, the two frameworks are complementary: MM/MD productions provide the mechanistic understanding and form part of the historical formulation knowledge used to develop the ML framework, whereas the trained ML model rapidly proposes alternative formulations that can subsequently be synthesized and experimentally evaluated. Therefore, improvements observed for specific epitope targets should be interpreted as evidence of the complementary nature of the two approaches (mechanistic and data‐driven), rather than superiority of one over the other.

For reference, all linear and isotherm fittings of the experimental data obtained for epitope affinity studies are illustrated in Figures S5–S7.

To further explore the experimental trends observed across the investigated formulations, we examined whether the MD‐predicted structural propensities of the template epitopes were qualitatively associated with the experimental nanoMIP profiles (Figure 5H–K). Epitopes exhibiting mixed MD‐predicted structural propensities (SS_sheet‐helix‐loop‐sheet) tended to show the largest changes in particle descriptors following ML‐based reformulation, whereas predominantly loop‐like propensities were associated with comparatively smaller changes. Although these observations do not establish epitopes' MD‐predicted structural propensities as independent determinants of nanoMIP behavior, they suggest that embedding these structural descriptors, alongside sequence, MM/MD molecular interactions, and experimental data, may capture information relevant to ML‐formulation prediction, consistent with the complementary, multimodal framework proposed herein.

Regarding the relationships between most relevant descriptors across the synthesized and characterized nanoMIPs, strong negative correlations were found between PDI and nanoMIP linear fit of epitope binding (−0.60), while PDI showed a moderate positive correlation with particle hydrodynamic sizes (0.37) (Figure S4). The ζ‐potential correlated negatively with both hydrodynamic sizes (−0.43) and PDI (−0.52), consistent with particles exhibiting greater surface charge magnitude tending to be more uniformly distributed, and less aggregated. Additional correlations are provided in Figure S4.

The following sections compare the affinity and specificity of both MM/MD‐guided and ML‐predicted nanoMIPs toward the target epitopes and corresponding proteins, using complementary electrochemical and SPR detection platforms. Correlations between key experimental descriptors such as R^2^ values from binding isotherms fitting, hydrodynamic sizes, polydispersity, heterogeneity (*m*), and dissociation constants (K_d_), are also examined.

### 2.5 Affinity Studies Toward Epitopes and Correlations Between nanoMIP Descriptors

The concentration range of interest for epitope (peptide) rebinding assays is 0.01–10^6^ pg/mL. All nanoMIPs showed linear responses over the tested range, with R^2^ values between 0.86 and 0.97. For most assays, both MM/MD and ML formulations showed similar R^2^ values, with negligible differences within ∼0.01–0.05; though ML‐based nanoMIP sensors often displayed steeper slopes, indicating potentially higher sensitivity.

The strongest epitope concentration dependencies were obtained for β2M nanoMIPs, ML‐based BRCA1 and BRCA2 nanoMIPs. In contrast, the lowest slopes were observed for CD20 and CD22 nanoMIPs (0.67–2.54). This finding suggests that among all particles, β2M nanoMIP‐based sensors may exhibit higher sensitivity, while the CD20 and CD22 ones were the least sensitive under this linear approximation.

While there was no correlation (r = 0.00 in Pearson correlation analysis, Figure S4) between hydrodynamic sizes and R^2^ of the linear fit for epitope rebinding, polydispersity trends were negatively correlated with both the linear fit of rebinding (−0.60) and the R^2^ fit of Langmuir isotherms (−0.53). These findings highlight that a more uniform particle distribution (lower PDI) may result in more homogeneous and better‐fitting peptide rebinding behavior (Figure S4). Higher heterogeneity index values, reflecting more homogeneous binding sites, were associated with stronger Langmuir‐type behavior (0.97), significantly weaker Freundlich characteristics (−0.94), and lower dissociation constants (−0.54). Collectively, these correlations indicate that particle uniformity and binding‐site homogeneity are more closely associated with epitope rebinding behavior than hydrodynamic size alone. The experimentally observed relationships provide valuable feedback for the iterative refinement of the complementary MM/MD‐ML nanoMIP formulation framework.

Additional insights into epitope rebinding, including linear and isotherm fittings, are provided in Figures S5–S7.

### 2.6 Cancer Protein Biomarker Detection and Specificity Studies

After verifying that nanoMIPs can recognize their templated epitope with high sensitivity, the binding response toward the target cancer biomarkers was subsequently evaluated using electrochemical techniques (Figure 6A). Across all nanoMIPs, the lowest protein rebinding linear fits were observed for MD‐based CD22 and β2M nanoMIPs (Figure S8A, G), consistent with peptide binding assays. ML formulations were associated with improved R^2^ values, from 0.78 to 0.96 for β2M nanoMIPs (Figure S8B) and from 0.89 to 0.95 for CD22 nanoMIPs (Figure S8H). Both ML‐based BRCA1 and CD20 nanoMIPs also showed stronger R^2^ fits, compared to MD‐based recipes (Figure S8C‐F).

**FIGURE 6:**
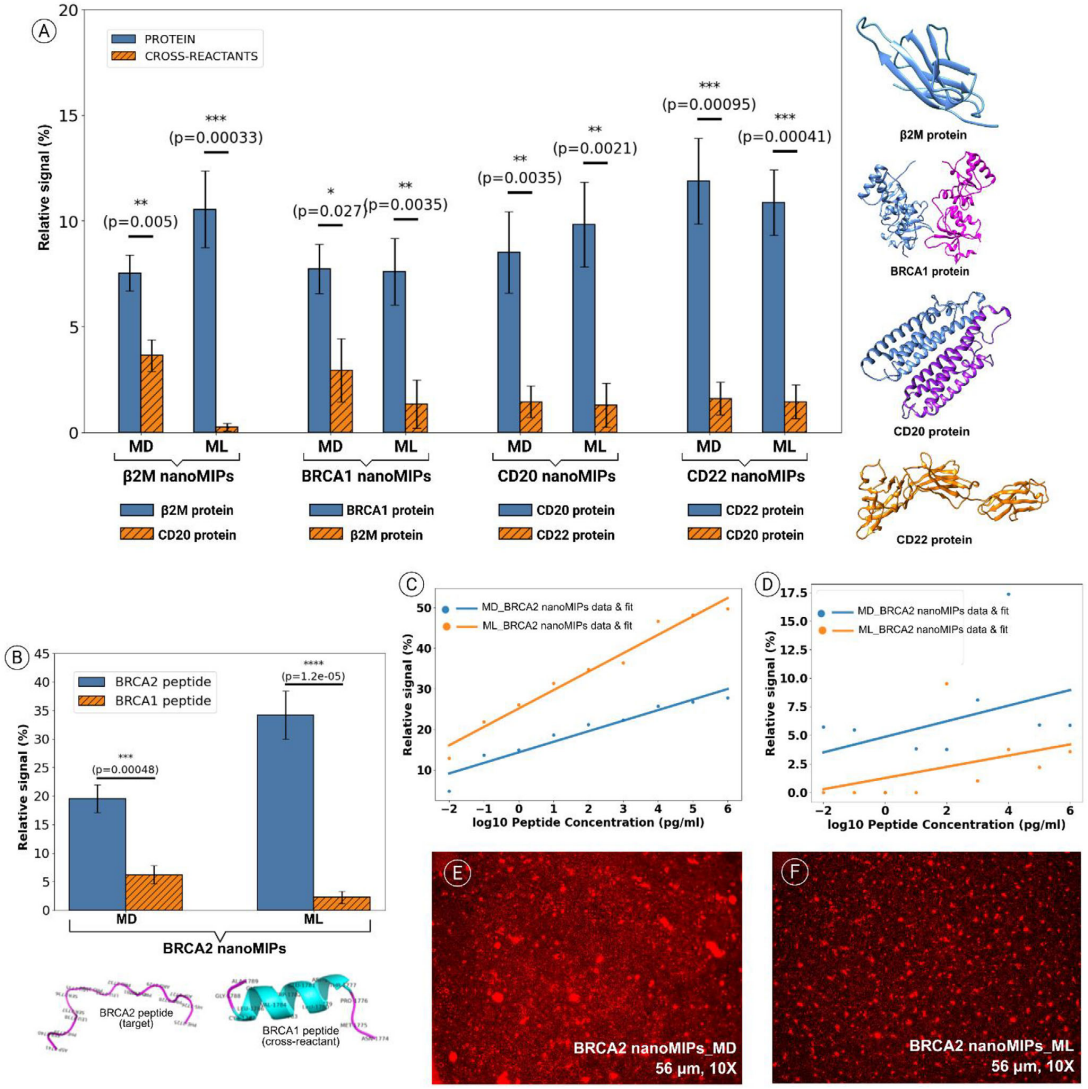
Cancer protein marker detection and cross‐reactivity studies conducted via electrochemical assays (A) highlight the comparative sensing performance of the two design strategies for β2M, BRCA1, CD20, and CD22 nanoMIPs. For BRCA2 nanoMIPs, affinity and specificity analyses (B) further confirm the receptor‘s preferential binding to BRCA2 peptide over the BRCA1 sequence. Concentration‐dependent affinity (C) and cross‐reactivity (D) profiles refine this characterization, revealing how nanoMIP‐peptide interactions evolve across the concentration range. Fluorescence imaging of the screen‐printed gold electrodes used in the sensor platform (E, F) visually confirms nanoMIP adsorption, with the particles appearing as distinct red features.

The sensor response increased with protein concentration for all investigated nanoMIPs. Pearson (*r* = 0.885‐0.994) and Spearman (ρ = 0.893‐0.990) correlation analyses confirmed strong concentration‐dependent rebinding across the investigated concentration range (10 pg/mL µg/mL), while the ML‐derived formulations generally indicated steeper responses, particularly at higher analyte concentrations (10–1000 ng/mL).

NanoMIP specificity was investigated using electrochemical assays against non‐targeted proteins (Figure 6A). All nanoMIPs showed significantly weaker binding toward the cross‐reactants than toward their target proteins (Mann‐Whitney U, p<0.05), supporting selective molecular recognition (Figures 7A, S9), with reductions in mean relative binding signals by ~52%‐97% for ML‐based nanoMIPs. Moreover, compared to MM/MD recipes, ML nanoMIPs generally showed higher R^2^ values for Langmuir fitting, as a potential indicator for more homogeneous binding behavior. In addition, BRCA1 peptide was used as a cross‐reactant for BRCA2 nanoMIPs. As shown in Figure 6B–D, both MM/MD‐ and ML‐derived BRCA2 nanoMIPs displayed minimal BRCA1 binding while maintaining a strong response toward BRCA2, further supporting their selective recognition. Taken together, these findings suggest that, for the investigated epitopes, our supervised ML framework generates nanoMIP formulations associated with reduced non‐specific binding while preserving target‐specific recognition.

The binding isotherm fittings for protein detection are presented in Figure 7.

**FIGURE 7:**
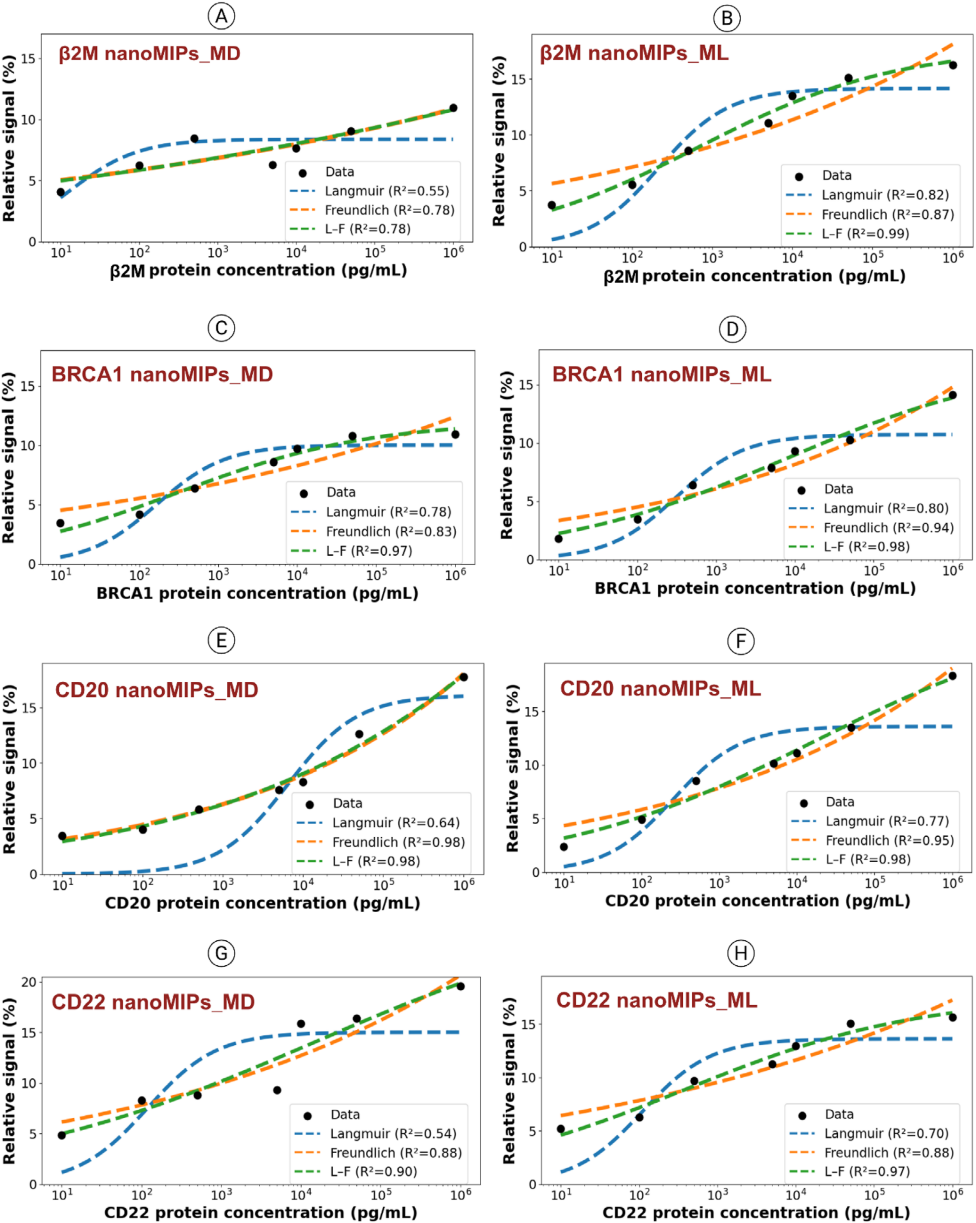
Isotherms (Langmuir, Freundlich, and Langmuir‐Freundlich) fitting of the affinity assays toward proteins across CD20, CD22, BRCA1, and β2M nanoMIP formulations: MD (A, C, E, G) vs ML (B, D, F, H). The concentration range of interest is 10 pg/mL, 100 pg/mL, 500 pg/mL, 5000 pg/mL, 10000 pg/mL, 50000 pg/mL, and 1000000 pg/mL.

Regression analysis was used to investigate pair‐wise relationships between root‐mean‐square‐error (RMSE) of L‐F protein rebinding fits and the nanoMIPs' hydrodynamic sizes, ζ‐potential, and L‐F heterogeneity indices. Pearson heatmap (Figure 8A) shows that binding sites homogeneity strongly correlates with protein L‐F fit quality (*r* = 0.76, p<0.05), while Spearman (Figure 8B) analysis confirms this through a strong negative correlation with RMSE (ρ = 0.74, p<0.05), indicating that as *m* rises toward more homogeneous affinity states, the fit error decreases.

**FIGURE 8:**
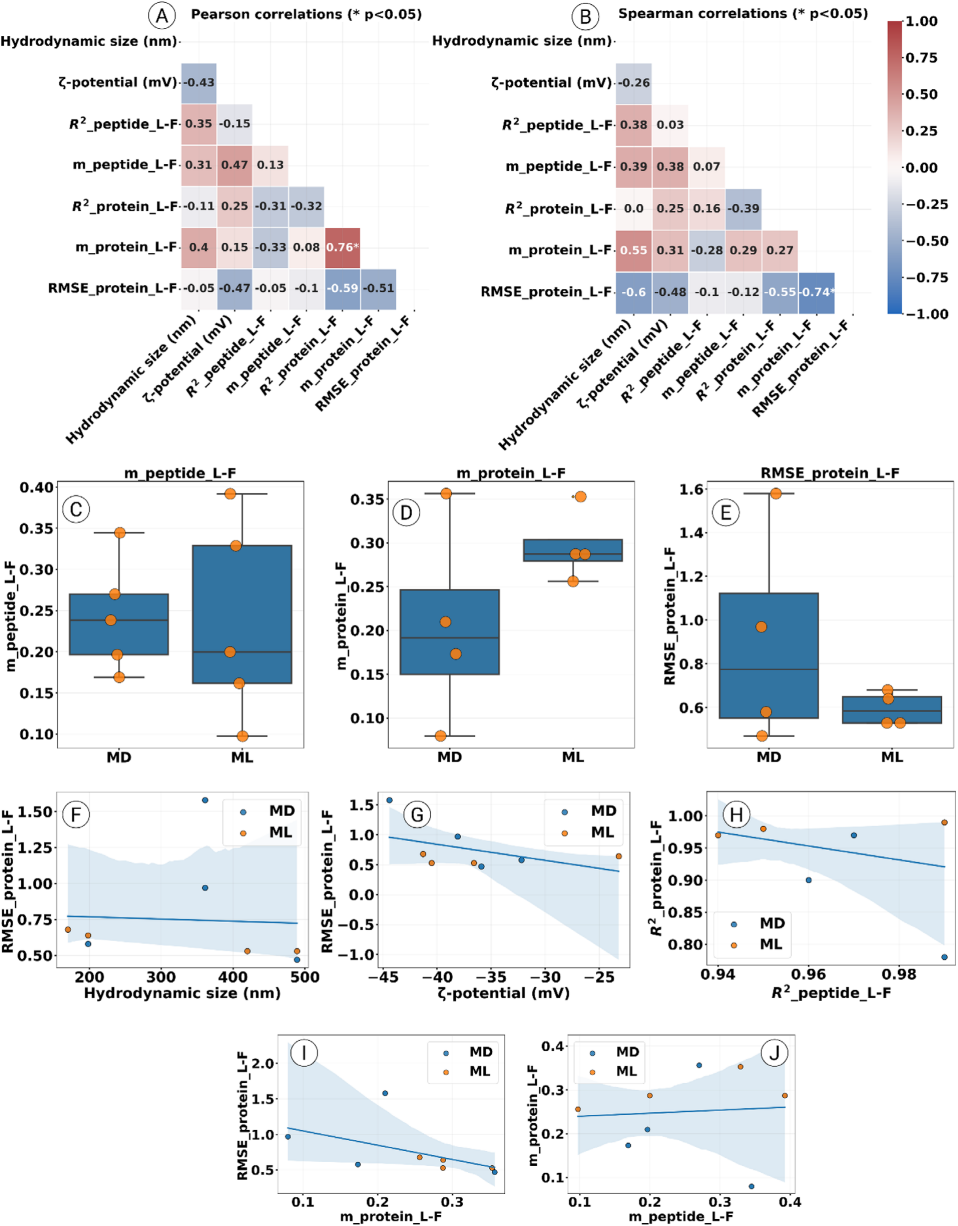
Comparative analysis of MM/MD vs ML‐derived nanoMIP descriptors. Linear and monotonic correlation analyses (A, B) reveal relationships among key nanoMIP physicochemical and performance features. Boxplots of binding‐site heterogeneity for peptide (C) and protein (D) rebinding, together with protein binding RMSE values (E), highlight systematic differences between MM/MD and ML formulations. Complementary regression analysis (F‐J) further quantifies these MM/MD vs ML distinctions across the same descriptors, extending the comparison to hydrodynamic size and ζ‐potential. Descriptive statistics were first calculated for all variables. Pearson and Spearman correlation coefficients were then computed for the numerical variables, together with corresponding p‐values; significance was assessed at α = 0.05. Homogeneity of variance was assessed using Levene's test, and normality using Shapiro‐Wilk test. Analyses are intended to identify trends and potential associations, rather than establish definitive statistics.

Moreover, ML‐based nanoMIPs exhibited higher and more consistent heterogeneity indices (Welch's T‐test, p = 0.041), and lower protein rebinding RMSE (Mann‐Whitney U, p = 0.032) than the corresponding MM/MD nanoMIPs (Figure 8C–E). Protein rebinding RMSE (Figure 8F–I) was independent of nanoMIPs hydrodynamic sizes, with only a slight tendency to decrease as ζ‐potential (in magnitude) increases (Figure 8I). Moreover, the binding‐site heterogeneity indices of nanoMIPs rebinding both peptide and proteins were only weakly associated (Figure 8J), while L‐F fit quality for peptide rebinding does not necessarily predict protein rebinding performance. Given the focused nature of this dataset, these comparisons are intended to identify potential trends and associations rather than establish definitive statistical significance or direct causal relationships.

To assess nanoMIPs batch‐to‐batch reproducibility, additional electrochemical assays were performed for both protein affinity and nanoMIP specificity using independent SPGE surfaces (Figure S10 and S11). For cross‐reactivity studies on the new SPGEs, an expanded panel of cross‐reactants was used to allow a broader assessment across multiple biomarkers.

### 2.7 SPR‐Based Affinity Studies Against Cancer Protein Biomarkers

The nanoMIPs affinity toward their respective target proteins was evaluated using surface plasmon resonance (SPR)‐based characterization (Figure 9). NanoMIPs were injected over immobilized protein surfaces to obtain real‐time sensograms (Figure 9A–E), and the resulting data were fitted to a 1:1 Langmuir binding model. Kinetic data, fitting parameters and dissociation constants (K_d_) for each nanoMIP are listed in Table S9. Across all targets, K_d_ values fell within the 10^−^^1^ ^3^–10^−^^10^ M range (Table S9), consistent with previously reported high‐affinity synthetic receptors [31, 32], confirming that both the MM/MD‐based and ML‐predicted formulations generated high‐affinity nanoMIPs for the investigated targets.

**FIGURE 9:**
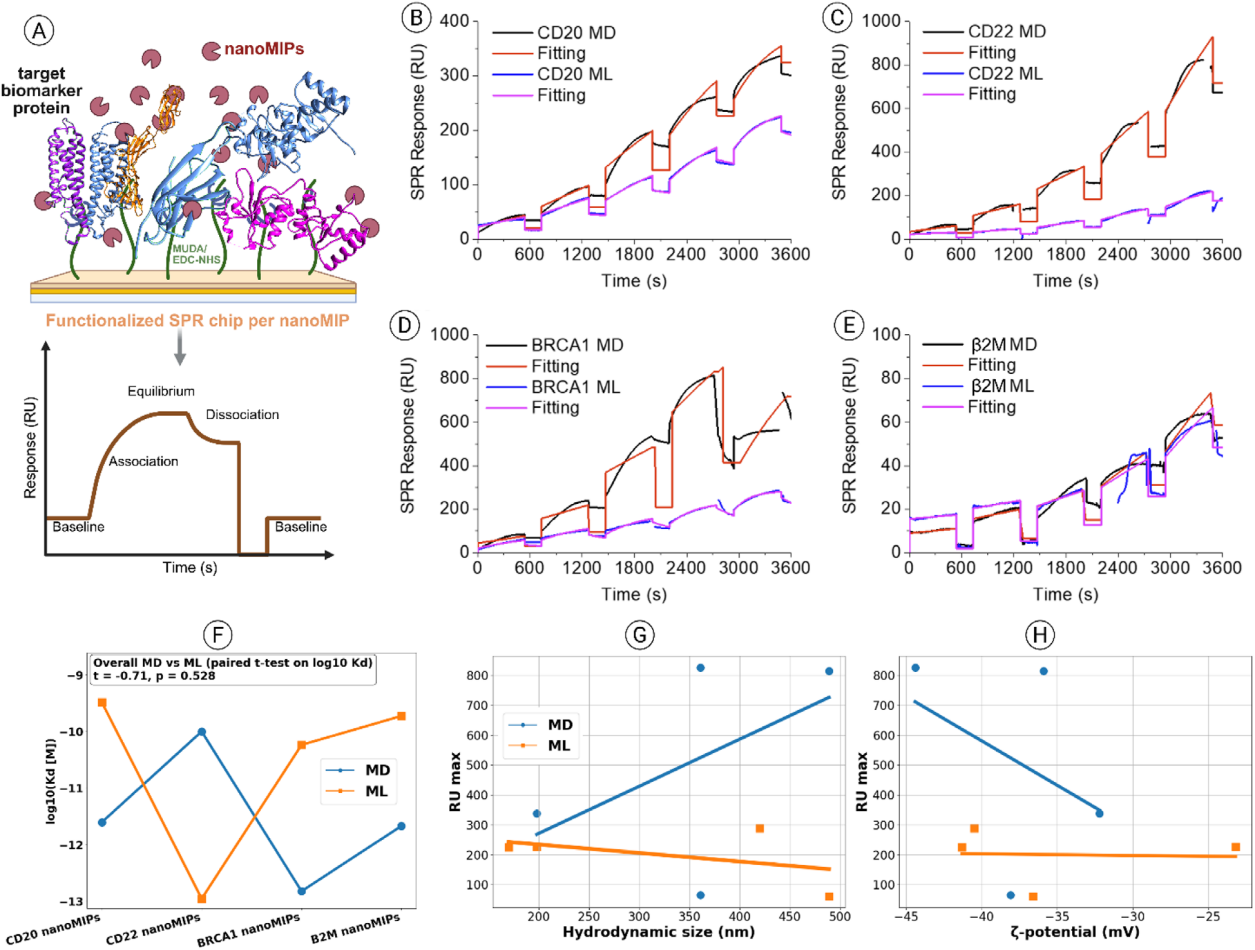
SPR‐based cancer protein binding kinetics (A). Real‐time sensograms and their corresponding Langmuir fits for CD20 (B), CD22 (C), BRCA1 (D), and β2M (E) epitope‐imprinted nanoMIPs reveal differences in association and dissociation behavior between MD‐ and ML‐guided formulations. A paired t‐test comparing the resulting K_d_ values (F) quantifies these performance differences as non‐statiscally significant (significance threshold of α = 0.05). Scatter plots with linear regression fits (G, H) further relate maximum response units (RU) to particles hydrodynamic size and ζ‐potential, respectively.

MM/MD‐driven nanoMIPs for CD20, BRCA1, and β2M showed lower K_d_ values than their ML counterparts, whereas ML‐based formulation exhibited a substantially lower Kd for CD22 than its MM/MD counterpart (Table S9). Geometric mean affinities were ∼3.0 × 10^−^^1^ ^2^ M for MD nanoMIPs and ∼2.5 × 10^−^^1^ ^1^ M for ML, suggesting a tendency toward lower Kd values for the MM/MD formulations. However, this difference was not statistically significant (paired t‐test, t = −0.71, p = 0.53, Figure 9F). Both approaches therefore consistently yielded binders with K_d_ in the pM range, demonstrating strong binding affinity regardless of the formulation design method.

Among the investigated targets, however, the ML‐derived CD22 nanoMIPs showed enhanced binding kinetics, with an ∼900‐fold lower Kd than the MM/MD formulation. This improvement coincides with substantially smaller hydrodynamic size (198 nm) and lower polydispersity index (PDI = 0.294) of the ML‐derived formulation, compared to the MM/MD counterpart (361 nm; PDI = 0.621), representing the sharpest physicochemical contrast observed between the two formulation strategies. Notably, the CD22 epitope also exhibited the most complex MD‐based structural propensities among investigated targets, including β‐sheets, helical, and loop components. Although these observations do not establish a causal relationship, they suggest that the complementary MM/MD‐ML framework can identify effective alternative recipes, and may be particularly valuable when physics‐based MM/MD formulations can be further refined through data‐driven predictions.

Additional analyses showed a broader distribution of RU values for MM/MD‐based nanoMIPs (SD ∼374) than for ML‐based formulations (SD ∼97), indicating more consistent responses for the latter. Interestingly, both MM/MD‐ and ML‐driven β2M nanoMIPs demonstrated lower RU values than the other receptors developed in this work, potentially reflecting their larger hydrodynamic sizes exceeding the effective penetration depth of the evanescent field. The RU values correlated positively with particle hydrodynamic size for MM/MD nanoMIPs (0.53) but negatively for ML‐based ones (−0.74) (Figure 9G,H); however, differences in mean and maximum RU were not significant (p>>0.05), limiting further interpretation given the small sample size. Overall, SPR analysis independently confirmed that both formulation strategies yield high‐affinity nanoMIPs, while the marked improvement observed for CD22 nanoMIPs points to the potential of supervised data‐driven prediction to refine specific formulations within the complementary MM/MD‐ML framework.

### 2.8 Cancer Protein Biomarker Detection in Human Serum

The performance of the developed nanoMIP‐based sensors was further evaluated in a more complex biological matrix using electrochemical binding assays in 10% human serum spiked with the target proteins.

Across all nanoMIPs, the logarithmic regression analysis of binding response demonstrated a consistent concentration‐dependent signal increase, indicating preserved target recognition in the presence of serum (Figure 10). Both MM/MD‐ and ML‐derived nanoMIP formulations exhibited comparable slopes for most target proteins, suggesting similar calibration sensitivity between the two formulation strategies. A notable exception was observed, once again, for the CD22 nanoMIPs, where the ML formulation displayed a steeper slope. Moreover, ML‐derived nanoMIPs also frequently exhibited higher intercepts, corresponding to increased baseline signal levels. Collectively, these results show that concentration‐dependent protein biomarker recognition is retained by both MM/MD‐ and ML‐nanoMIPs despite serum interference.

**FIGURE 10:**
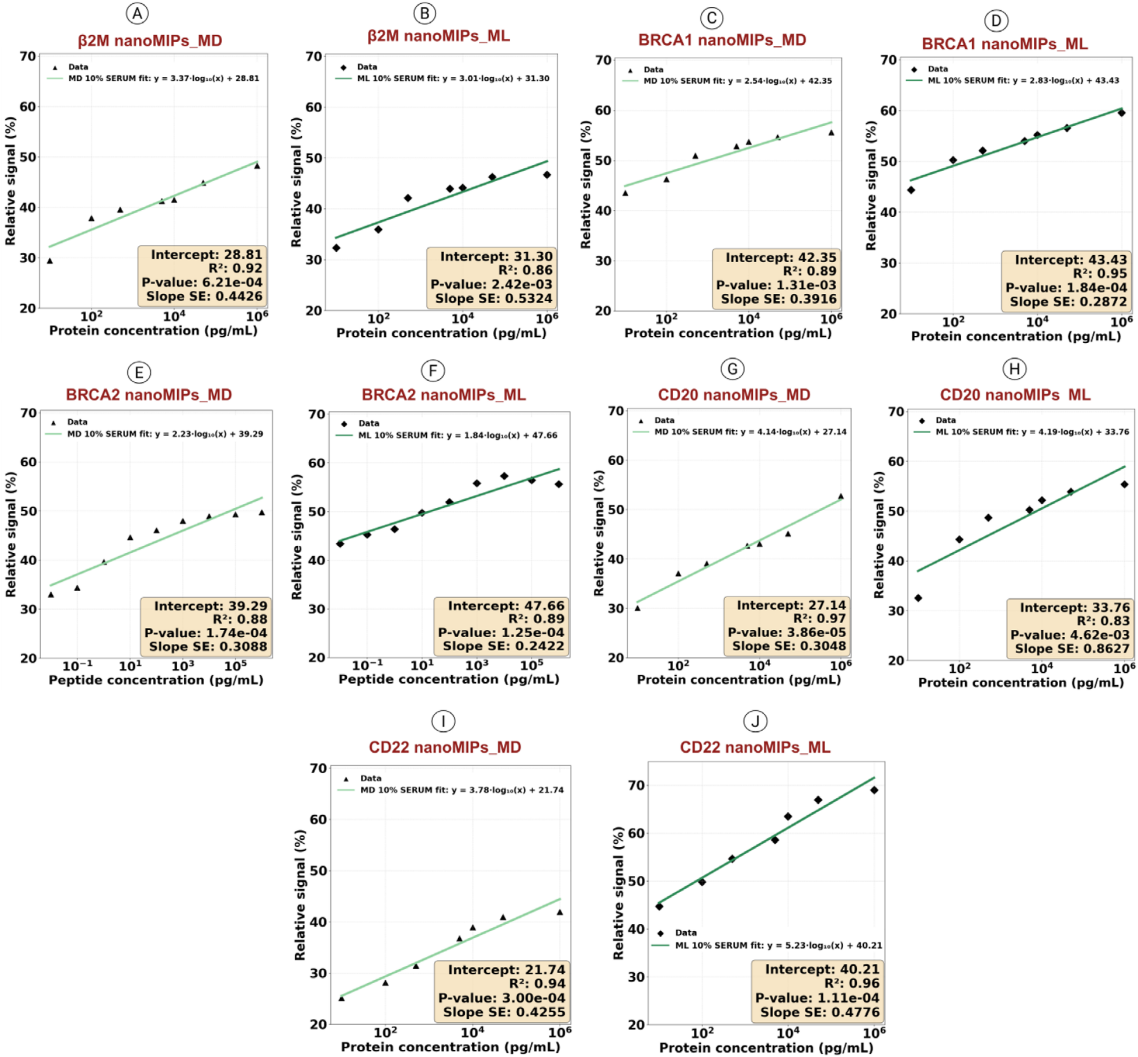
Electrochemical binding assays conducted in 10% human serum spiked with the target protein biomarkers. Linear fit of the experimental data across nanoMIP formulations: MD (A, C, E, G, I) and ML (B, D, F, H, J). The concentration range of the target protein analytes (A‐D, G‐J) in 10% human serum is 10 pg/mL, 100 pg/mL, 500 pg/mL, 5000 pg/mL, 10000 pg/mL, 50000 pg/mL, and 1000000 pg/mL. The concentration range of peptide in 10% serum (E, F), corresponding to BRCA2 nanoMIPs, is 0.01‐10^6^ pg/mL.

## 3 Conclusion

This study presents a proof‐of‐concept approach for rapid, target‐specific nanoMIP formulation, combining physics‐based MM/MD productions with supervised ML and experimental evaluation. Trained on epitope fragments of up to 20 amino acids, our ML framework supports epitope‐mediated imprinting [33–35] and generates nanoMIP recipes suitable for, but not limited to, solid‐phase synthesis. A central contribution is the integration of MM/MD‐derived, experimental, and epitope sequence‐derived information into a curated training dataset, allowing the RF model to learn formulation‐class relationships from both molecular interactions and previously observed nanoMIP behavior. Tested on five unseen cancer biomarker epitopes, the baseline RF model achieved 93% accuracy in formulation‐class prediction, with F1‐scores of 92%–96% across the three formulation classes. Once trained, the complete framework translates the predicted formulation class into a synthesis‐ready nanoMIP recipe for previously unseen epitopes within seconds. By integrating MM/MD with supervised ML workflows within a multimodal formulation framework, we highlight critical features, uncover correlations, and capture molecular‐level information that is difficult to resolve experimentally.

Importantly, the purpose of our ML framework is not to outperform MM/MD, and the experimental results further show that neither formulation strategy is consistently superior across the investigated nanoMIP formulations. Nevertheless, notable formulation‐dependent differences were observed, with ML‐predicted nanoMIP formulations improving particle size and monodispersity for specific targets, as well as reducing cross‐reactivity. However, rather than competing approaches, in this study, MM/MD and ML play complementary roles. Mechanistic understanding identified the molecular interactions and physicochemical descriptors relevant to nanoMIP formulation, while recognizing that these describe the pre‐polymerization recognition stage rather than the final material. Supervised data‐driven predictions made this accumulated physics‐based and experimental knowledge rapidly reusable for previously unseen target epitopes, without requiring the full design process to be repeated for each new target epitope. Experimental evidence then ensured that the proposed formulations actually produced functional nanoMIPs and provided new information that can further refine subsequent predictions.

As additional MM/MD and experimental data are incorporated, this approach can be progressively refined and extended to a broader nanoMIP formulation space, reinforcing how mechanistic understanding, data‐driven prediction, and experimental evidence can work together to guide nanoMIP development.

## 4 Methods

### 4.1 Molecular Mechanics (MM) and Molecular Dynamics (MD)

Epitope candidates were identified via MD simulations based on multiple criteria, including solvent‐accessible surface area (SASA), atomic‐root‐mean‐square fluctuations (RMSF) per residue, structural stability, location within whole protein structures, and overall structural diversity. To ensure comparability and experimental feasibility, selected epitopes were limited to a maximum length of 20 amino acids. In addition, Circular Dichroism (CD) measurements of the isolated, selected epitopes are shown in Figure S12 and discussed in the Supporting Information.

Seventeen FMs were screened via molecular docking using AutoDock Vina [36], followed by the selection of top candidates based on binding affinities, H‐bonds, and root‐mean‐square deviation (RMSD) values. The molecular structures of all monomers were retrieved from PubChem database [37]. Visualization and analysis of the docking results were accomplished using UCSF Chimera [38]. Subsequently, the pre‐selected and docked FMs were used in low‐ (1:3, 1:5) and high‐ratio (1:10, 1:15) MD simulations, with N,N'‐methylenebisacrylamide (BIS) as crosslinker, which was considered only in underperforming MIP complexes.

MD simulations were conducted to assess the stability and dynamic behavior of T‐FMs docked complexes using Gromacs package [39]. All systems were solvated with TIP3P water model, neutralized using Na^+^/Cl^−^ ions and energy minimized in 50 000 steps using Steepest Descent method [40]. CHARMM36 force field [41] was employed for all productions, under NVT (constant number of particles, volume, and temperature) and NPT (constant number of particles, pressure, and temperature) ensembles, with equilibration at 298 K and 1 bar. Both NVT and NPT equilibrations were performed for 1000 ps. All MD productions in NPT ensemble were carried out for 200 ns per system, with periodic boundary conditions (PBC) applied, and without constraints. The full list of monomers, SS propensities and amino acid sequences of epitopes, force field description, and further computational setup details are provided in Figure S13.

### 4.2 ML Algorithm Development

The developed ML framework predicts nanoMIP formulation recipes from the amino‐acid sequence of the targeted epitopes (1‐letter code, ≤ 20 residues). For a new epitope, sequence‐derived descriptors are calculated and used within the trained framework, with the RF model predicting the corresponding formulation class (Figure 3). This prediction is subsequently translated through chemistry‐informed selection criteria into a synthesis‐ready recipe. The framework outputs the recommended monomer types, their ratios per template molecule, and whether crosslinking monomers should be included and in what ratio. Thus, no new MM/MD simulations are required for an unseen epitope during deployment. Model performance metrics are also reported. The mechanistic interpretation presented in this work was obtained during model fine‐tuning and deployment through the analysis of molecular descriptor importance, alongside comparison and correlation with the obtained experimental nanoMIP data. Thus, while users receive nanoMIP formulation predictions strictly from the epitope sequence input, the mechanistic insights discussed in this work reflect the analysis performed during algorithm development and RF model deployment.

Historical experimental descriptors are used during model training and therefore influence subsequent formulation class predictions. For new epitope sequences, the framework relies on sequence‐derived features, with experimental and MM/MD data influencing the predictions indirectly through the patterns learned during training.

Python version 3.8.10 was used for scripting. The model's performance was assessed using metrics such as accuracy, precision, recall, and F1‐score. The dataset was split into training and testing sets using 70/30 ratio. The input features and their mapping used for our training dataset development are illustrated in Figure S14 and Tables S10 and S11. The selection criteria functions evaluate the conditions based on sequence‐derived descriptors, specific features, and return a labeled classification (e.g. ‘Good’) if the criteria are met. For example, one condition requires the epitope sequence to contain at least one acidic or polar residue (acidic_count >0 or polar_count >0). Other conditions include, among others, specific numerical ranges of gyration values, minimum distances, H‐bonds, and total interaction energies for all potential classes: ‘Good’, ‘Bad’ and ‘Optimal’ nanoMIP components. Importantly, all these conditions are combined using the logical *and* operator, meaning every single condition must be true for the entire expression to be true. Herein, the MD‐based structural propensities of template molecules and their physicochemical properties, alongside their involvement in dynamic interactions with distinct monomers, are combined. In this way, sequence‐derived properties are considered together with the molecular and formulation relationships represented in the training dataset.

Finally, for an unseen peptide sequence, the RF‐predicted formulation class is combined with the chemistry‐informed selection criteria to generate the final, synthesis‐ready nanoMIP formulation. Details on the selection criteria function, mapping of monomers to their respective amino acid types, and crosslinking ratio dependencies are provided in the SI.

### 4.3 NanoMIPs Synthesis and Characterization Methods

All nanoMIPs were synthesized using the solid‐phase method [16, 17], detailed in SI and illustrated in Figure S15, along with the monomer chemical structures and polymerization mixtures (Table S12). Moreover, all chemicals, reagents, and apparatus, as well as the obtained nanoMIP yields (Figure S16), are provided in the SI. Regarding nanoMIPs characterization, hydrodynamic size and surface charge measurements were conducted on a Zetasizer Pro (Malvern Panalytical Ltd, Herrenberg, Germany) after 3 mL of synthesized nanoMIPs were sonicated for 25 min at 20°C–25°C, and filtered using 0.45 µm pore diameter filters. The sample volume used in the capillary cell (Malvern cuvette) was 700 µL. The equilibration time was set for 200 s at 25°C, and three measurements for both dynamic light scattering (DLS) and ζ‐potential were recorded during each acquisition. The methodology presented herein, including synthesis and DLS/ζ‐potential measurements, was consistently applied using the same parameters across all nanoMIP formulations.

### 4.4 Sensitivity and Specificity Studies: Electrochemical and SPR Assays

Peptide and protein detection assays, along with cross‐reactivity studies for all nanoMIPs, were performed using electrochemical sensors. NanoMIPs were immobilized on screen‐printed gold electrodes (SPGEs) by physical adsorption (drop‐casting), chosen to maintain consistency across all formulations, since only ML‐based CD22 and β2M nanoMIPs contain free amines from APMA for potential EDC/NHS coupling, and to better preserve recognition site availability. Fluorescence imaging (Figure S17, Figure 5E,F) confirmed stable immobilization and retention of nanoMIPs onto the SPGEs throughout the measurements. Further electrochemical assay details are provided in the SI. In parallel, SPR assays were conducted to evaluate protein‐nanoMIP binding kinetics and affinity, with the cancer proteins immobilized first and nanoMIPs injected afterward for real‐time analysis (see SI for details).

### 4.5 Statistical Analysis

All statistical models and methods used in this study are detailed in the SI.

## Author Contributions

N.S.M‐C. contributed to study conceptualization, performed the experimental studies, developed the algorithm, conducted all related analyses, and drafted the manuscript. T.B.S. carried out the computational and SPR‐based studies, and contributed to drafting the manuscript. E.S. guided and performed the SPR‐based studies and related analyses, and contributed to manuscript drafting and revision. R.A. contributed to manuscript revision. Z.A. conceptualized and supervised the study, secured funding, led the project, guided the experiments, and critically revised and improved the manuscript.

## Conflicts of Interest

The authors declare no conflicts of interest.

## Supporting information




**Supporting File**: advs78276‐sup‐0001‐SuppMat.pdf.

## Data Availability

The data that support the findings of this study are available from the corresponding author upon reasonable request.
