# Supplementary material for: Data‐Driven Design of Biofunctional Molecularly Imprinted Polymers for Precision Diagnostics
Source: Adv Sci (Weinh). 2026 Nov 9:e78276. Online ahead of print. doi: 10.1002/advs.78276 (PMC13748772; doi:10.1002/advs.78276)
Supplement: Supplementary file 1 [file ADVS-9999-e78276-s001.pdf]

Supporting Information

Data-Driven Design of Biofunctional Molecularly Imprinted Polymers for Precision Diagnostics

Nastasia Sanda Moldovean-Cioroianu[1], Tamim B. Sarwar[1], Ekin Sehit[1], Roland Aydin[2],

*Zeynep Altintas[1,]**

[1] Bioinspired Materials and Biosensor Technologies, Institute of Materials Science, Faculty of Engineering, Kiel University, 24243 Kiel, Germany

[2] Institute of Continuum and Material Mechanics, Hamburg University of Technology, Eißendorfer Straße 42, 21073 Hamburg, Germany

* Corresponding author: Z. Altintas
E-mail: zeynep.altintas@tf.uni-kiel.de

List of Abbreviations

AA: Acrylic Acid;
AAM: Acrylamide;
AMPSA: 2-acrylamido-2-methyl-1-propane sulfonic acid;
AN: Acrylonitrile;
APMA: N-(3-aminopropyl) methacrylamide;
B2M: beta-2-Microglobulin;
BAAPy: 2,6-Bis(Acrylamido)Pyridine;
BIS: Methylenebisacrylamide;
BMA: Benzyl Methacrylate;
BRCA1: breast cancer type 1 susceptibility protein;
BRCA2: breast cancer type 2 susceptibility protein;
BRCT: BRCA1 C Terminus;
CD20: cluster of differentiation 20;
CD22: cluster of differentiation 22;
DLS: Dynamic light scattering;
EMA: Ethyl Methacrylate;
FM: Functional Monomer;
GRAVY: Grand Average of Hydropathicity;
HEMA: 2-Hydroxyethyl Methacrylate;
LOD: Limit of detection;
MAA: Methacrylic Acid;
MD: Molecular Dynamics;
MEP: 2-methacryloyloxyethyl phosphate
ML: Machine learning;
MM: Molecular Mechanics;
MMA: Methyl Methacrylate;
NanoMIPs: Molecularly Imprinted Polymer nanoparticles;
NIPAM: N-Isoproplacrylamide:
NOBE: N,O-bis methacryloyl ethanolamine;
NVI: N-Vinylimidazole;
NVP: N-Vinylpyrrolidone;
PDI: Polydispersity index;
RF: Random Forest;
RFE: Recursive Feature Elimination;
RMSF: Root mean square fluctuation;
SASA: Solvent accessible surface area;
SPR: Surface Plasmon Resonance;
SWV: Square Wave Voltammetry;
SPGE: Screen printed gold electrode;
T: Template;
TfMAA: 2-(trifluoromethyl)acrylic acid;
VI: Vinylimidazole.

2. Results

2.1. NanoMIPs design using a hybrid MM/MD method

To identify optimal imprinting targets, epitope candidates were extracted from whole targeted cancer biomarkers via MD simulations of protein biomarkers in explicit water model. Per-residue root-mean-square fluctuations (RMSF), solvent accessible surface area (SASA), and secondary structure propensity were evaluated over the production trajectories to assess conformational stability, surface exposure, and structural diversity of epitopes (Figure S1), within the predefined MD environment.

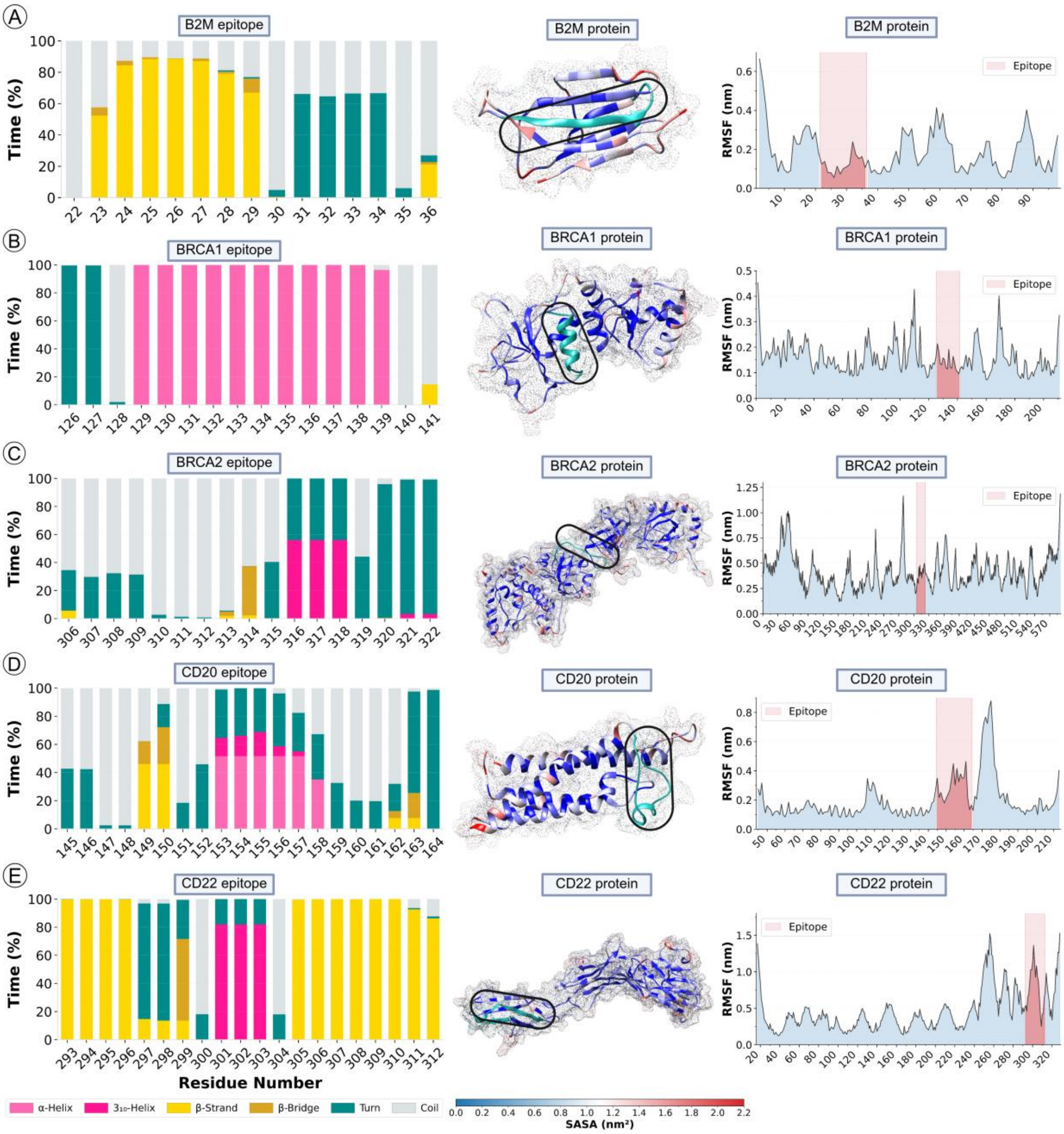

Figure S1. MD-derived secondary structure (SS) analysis of the selected epitopes, along with SASA and RMSF profiles and their localization within the full protein structures, supporting the structure-informed epitope selection.

All selected epitopes reside in solvent-exposed regions, as confirmed by elevated SASA values and their surface-accessible regions within whole proteins (Figure S1, middle panel). The average RMSF profiles (Figure S1, right panel) show that each epitope region has been extracted from highly exposed domains. However, in our unconstrained MD productions, RMSF-based selection inherently involves a certain trade-off between epitopes' structural rigidity and their dynamic accessibility, as highly flexible regions (e.g., loops) may exhibit increased solvent exposure and binding capability, whereas epitopes with intrinsically lower RMSF values (e.g., helices, β-strands) may offer better structural definition but slightly reduced accessibility. Secondary structure analysis shows that the selected epitopes span a broad range of structural motifs, from stable β-strands in β2M and CD22, to turn-dominated segments in BRCA2 and mixed α-helix/turn compositions in CD20.

Each epitope was selected from a functionally relevant region of its parent protein: the immunoglobulin fold of β2M,[1] BRCA1 epitope from the C Terminus (BRCT) domain,[2] the DNA-binding domain of BRCA2,[3] the extracellular loop of CD20,[4] and the Ig ectodomain of CD22.[5] Together, these criteria ensured that the selected epitopes present stable, surface-accessible, and structurally defined segments, providing well-defined templates for subsequent nanoMIP design via the hybrid MM/MD workflow.

Table S1. One letter-code of the amino acid sequences, and physicochemical properties of the seven cancer biomarker epitopes used in MM/MD studies. The SS propensities are derived exclusively from MD productions.

Biomarker of interest	Secondary structural components	Amino acid (AA) sequence (one-letter code)	GRAVY (*) score	Physicochemical properties (counts of AA types)
β2M	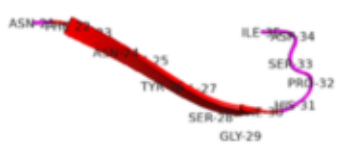	NFLNCYVSGFHPSDI	0.133	Acidic: 1, Basic: 1<br>Polar: 6, Nonpolar: 7
BRAF-V600	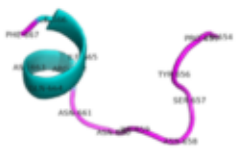	LPYSNINNRDQIIF	-0.400	Acidic: 1, Basic: 1<br>Polar: 6, Nonpolar: 6
MMP9	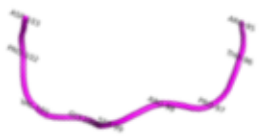	RTPRSGVPD	-1.489	Acidic: 1, Basic: 2<br>Polar: 2, Nonpolar: 4
BRCA1	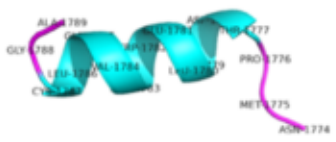	NMPTDQLEWMVQLCGA	-0.075	Acidic: 2, Basic: 0<br>Polar: 5, Nonpolar: 9
BRCA2	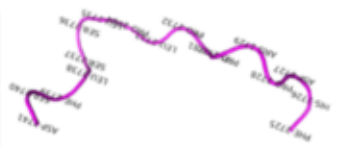	FHDPRPFPLPLSSLFSD	-0.218	Acidic: 2, Basic: 2<br>Polar: 3, Nonpolar: 10
CD20	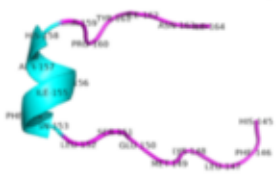	HFLKMESLNFIRAHTPYINI	0.035	Acidic: 1, Basic: 4<br>Polar: 5, Nonpolar: 10
CD22	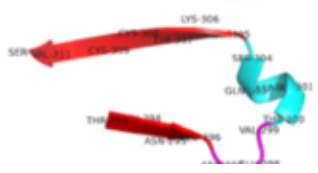	TLNLREVTKDQSGKYCCQVS	-0.675	Acidic: 2, Basic: 3<br>Polar: 10, Nonpolar: 5

(*) Grand Average of Hydropathicity (GRAVY) scores indicate the average hydrophobicity and hydrophilicity of peptide sequences. [6]

Positive GRAVY scores reflect more hydrophobic motifs, correlated with tendencies to form the folded core of proteins. In contrast, negative scores reflect more hydrophilic motifs, more solvent exposed and flexible regions, with greater solvent exposure and regional flexibility, often rich in polar and/or charged amino acids. The biomarker epitopes of interest in this study

account for both types of hydropathicity. However, the physicochemical properties of individual amino acids alone do not determine the overall behavior of a peptide sequence; rather, the sequence context, the specific order of amino acids, and the presence of other interacting partners have a great impact on peptide's structural and dynamic properties.

Table S2. Binding affinity scores between FMs and T molecules of interest derived from docking studies.

Functional monomer	β2M (kcal/mol)	BRAF-V600 (kcal/mol)	MMP9 (kcal/mol)	BRCA1 (kcal/mol)	BRCA2 (kcal/mol)	CD20 (kcal/mol)	CD22 (kcal/mol)
AA	-2.2	-2.4	-1.9	-2.2	-2.2	-2.5	-2.2
AAM	-2.2	-2.2	-1.9	-2.3	-2.2	-2.5	-2.1
AMPSA	-2.7	-3.2	-2.4	-2.9	-3.0	-4.0	-3.1
AN	-1.7	-2.0	-1.4	-1.7	-1.7	-1.8	-1.8
APMA	-2.7	-3.5	-2.5	-2.9	-3.1	-3.5	-2.9
BAAPy	-3.3	-4.0	-3.0	-3.7	-3.6	-4.2	-3.8
BMA	-1.8	-2.1	-1.6	-2.0	-2.0	-2.1	-2.0
DEAA	-2.2	-2.8	-1.9	-2.5	-2.8	-2.7	-2.6
EMA	-2.3	-2.5	-2.1	-2.4	-2.4	-2.6	-2.4
HEMA	-2.6	-3.1	-2.7	-2.9	-2.7	-3.1	-2.9
MAA	-2.4	-2.8	-2.1	-2.8	-2.6	-2.7	-2.6
MMA	-2.2	-2.5	-1.8	-2.3	-2.3	-2.4	-2.3
NIPAM	-2.6	-2.9	-2.6	-2.6	-2.8	-2.9	-2.8
NVI	-2.0	-2.3	-1.9	-2.2	-2.2	-2.4	-2.3
NVP	-2.4	-2.8	-2.1	-2.5	-2.7	-2.8	-2.7
TfMAA	-3.1	-3.3	-2.8	-3.2	-3.1	-3.6	-3.3
VI	-2.3	-2.5	-2.1	-2.4	-2.5	-2.3	-2.4

The best-performing and selected FMs against cancer biomarker templates are highlighted in blue.

Table S3. MD-derived metrics for T:FM formulations at lower ratios, including inter-atomic minimum distances (MinDist), total H-bonds, and total interaction energies per T-FMs combination.

MIP recipe	T – FM complex	MinDist T-FM (nm)	H-bonds T-FM	Total interaction energy (kJ/mol)
β2M – $AMPSA_1BAAPy_2HEMA_2NIPAM_3$	T – $AMPSA_1$	0.955	2045	-9.942
	T – $BAAPy_2$	0.585	3970	-26.715
	T – $HEMA_2$	0.807	806	-6.36
	T – $NIPAM_3$	0.613	1625	-11.006
BRAF-V600 – $AMPSA_1BAAPy_3HEMA_1NIPAM_3$	T – $AMPSA_1$	0.524	4256	-20.631
	T – $BAAPy_3$	0.265	13091	-85.062
	T – $HEMA_1$	0.657	1322	-7.77
	T – $NIPAM_3$	0.320	4653	-26.490
MMP9 – $AMPSA_1BAAPy_1HEMA_1NIPAM_1$	T – $AMPSA_1$	0.729	1170	-7.187
	T – $BAAPy_1$	0.628	1839	-13.913
	T – $HEMA_1$	0.788	569	-3.872
	T – $NIPAM_1$	0.795	501	-4.05
BRCA1 – $AMPSA_2BAAPy_1HEMA_1NIPAM_2$	T – $AMPSA_2$	0.283	5862	-46.291
	T – $BAAPy_1$	0.397	2343	-29.289
	T – $HEMA_1$	0.555	1022	-12.855
	T – $NIPAM_2$	0.347	2079	-24.423
BRCA2 – $AMPSA_2BAAPy_2HEMA_2NIPAM_2$	T – $AMPSA_2$	0.427	5223	-28.681
	T – $BAAPy_2$	0.412	4384	-33.347
	T – $HEMA_2$	0.519	1961	-13.388
	T – $NIPAM_2$	0.464	2260	-15.932
CD20 – $AMPSA_3BAAPy_1HEMA_1NIPAM_1$	T – $AMPSA_3$	0.279	12691	-64.627
	T – $BAAPy_1$	0.724	2793	-18.590
	T – $HEMA_1$	0.860	737	-5.784
	T – $NIPAM_1$	0.837	914	-7.018
CD22 – $AMPSA_2BAAPy_2HEMA_2NIPAM_1$	T – $AMPSA_2$	0.608	5949	-27.964
	T – $BAAPy_2$	0.526	7541	-41.259
	T – $HEMA_2$	0.786	1179	-8.549
	T – $NIPAM_1$	1.089	953	-6.636

Table S4. MD-derived metrics for T:FM formulations at 1:5 ratios, including inter-atomic minimum distances (MinDist), total H-bonds, and total interaction energies per T-FMs combination.

MIP recipe	T – FM complex	MinDist T-FM (nm)	H-bonds T-FM	Total interaction energy (kJ/mol)
β2M – $AMPSA_5BAAPy_5NIPAM_5$	T – AMPSA	0.292	16510	-73.271
	T – BAAPy	0.311	8293	-55.155
	T – NIPAM	0.417	3343	-19.573
BRAF-V600 – $AMPSA_5BAAPy_5NIPAM_5$	T – AMPSA	0.273	13089	-60.936
	T – BAAPy	0.281	10452	-56.070
	T – NIPAM	0.319	4449	-27.355
MMP9 – $AMPSA_5BAAPy_5NIPAM_5$	T – AMPSA	0.348	6155	-35.978
	T – BAAPy	0.379	7714	-43.734
	T – NIPAM	0.440	1923	-14.045
BRCA1 –	T – AMPSA	0.249	9263	-75.985

$AMPSA_5BAAPy_5NIPAM_5$	T – BAAPy	0.226	7825	-85.491
	T – NIPAM	0.285	2560	-37.217
BRCA2 – $AMPSA_5BAAPy_5NIPAM_5$	T – AMPSA	0.369	7568	-43.252
	T – BAAPy	0.356	11053	-68.063
	T – NIPAM	0.492	2581	-17.698
CD20 – $AMPSA_5BAAPy_5NIPAM_5$	T – AMPSA	0.235	17784	-94.452
	T – BAAPy	0.229	21542	-115.957
	T – NIPAM	0.258	5782	-41.460
CD22 – $AMPSA_5BAAPy_5NIPAM_5$	T – AMPSA	0.291	19037	-75.172
	T – BAAPy	0.311	9414	-55.101
	T – NIPAM	0.376	3990	-23.513

Table S5. MD-derived metrics for T:FM formulations at 1:10 and 1:15 ratios, including inter-atomic minimum distances (MinDist), total H-bonds, and total interaction energies per T-FMs combination.

MIP recipe	T – FM complex	MinDist T-FM (nm)	H-bonds T-FM	Total interaction energy (kJ/mol)
β2M – $AMPSA_{10}BAAPy_{10}NIPAM_{10}$	T – $AMPSA_{10}$	0.226	21360	-103.550
	T – $BAAPy_{10}$	0.221	18660	-107.147
	T – $NIPAM_{10}$	0.272	6631	-41.363
β2M – $AMPSA_{15}BAAPy_{15}NIPAM_{15}$	T – $AMPSA_{15}$	0.210	30142	-130.069
	T – $BAAPy_{15}$	0.207	25554	-134.032
	T – $NIPAM_{15}$	0.233	9933	-55.506
BRAF-V600 – $AMPSA_{10}BAAPy_{10}NIPAM_{10}$	T – $AMPSA_{10}$	0.224	23303	-98.104
	T – $BAAPy_{10}$	0.225	22203	-110.715
	T – $NIPAM_{10}$	0.258	6040	-69.136
BRAF-V600 – $AMPSA_{15}BAAPy_{15}NIPAM_{15}$	T – $AMPSA_{15}$	0.206	37823	-157.107
	T – $BAAPy_{15}$	0.214	25939	-132.529
	T – $NIPAM_{15}$	0.245	6958	-44.214
MMP9 – $AMPSA_{10}BAAPy_{10}NIPAM_{10}$	T – $AMPSA_{10}$	0.301	11634	-63.727
	T – $BAAPy_{10}$	0.273	13019	-65.832
	T – $NIPAM_{10}$	0.331	3281	-22.983
MMP9 – $AMPSA_{15}BAAPy_{15}NIPAM_{15}$	T – $AMPSA_{15}$	0.284	11211	-61.671
	T – $BAAPy_{15}$	0.275	11998	-64.329
	T – $NIPAM_{15}$	0.317	4892	-28.902
MMP9 – $AMPSA_{15}BAAPy_{15}NIPAM_{15}BIS_5$	T – $AMPSA_{15}$	0.278	12671	-62.217
	T – $BAAPy_{15}$	0.277	12128	-64.366
	T – $NIPAM_{15}$	0.342	4352	-25.494
MMP9 – $AMPSA_{15}BAAPy_{15}NIPAM_{15}BIS_{10}$	T – $AMPSA_{15}$	0.357	8773	-46.274
	T – $BAAPy_{15}$	0.371	8236	-44.388
	T – $NIPAM_{15}$	0.402	2923	-19.219
BRCA1 – $AMPSA_{10}BAAPy_{10}NIPAM_{10}$	T – $AMPSA_{10}$	0.213	16029	-106.925
	T – $BAAPy_{10}$	0.212	15273	-122.602
	T – $NIPAM_{10}$	0.248	4731	-50.827
BRCA1 – $AMPSA_{15}BAAPy_{15}NIPAM_{15}$	T – $AMPSA_{15}$	0.202	25115	-159.925
	T – $BAAPy_{15}$	0.226	15320	-130.559
	T – $NIPAM_{15}$	0.219	5669	-70.570
BRCA2 – $AMPSA_{10}BAAPy_{10}NIPAM_{10}$	T – $AMPSA_{10}$	0.203	32836	-151.93
	T – $BAAPy_{10}$	0.216	27874	-149.510
	T – $NIPAM_{10}$	0.254	7003	-46.314
BRCA2 – $AMPSA_{15}BAAPy_{15}NIPAM_{15}$	T – $AMPSA_{15}$	0.202	29835	-155.586
	T – $BAAPy_{15}$	0.209	24665	-152.841
	T – $NIPAM_{15}$	0.237	7610	-53.406
CD20 – $AMPSA_{10}BAAPy_{10}NIPAM_{10}$	T – $AMPSA_{10}$	0.196	34466	-173.827
	T – $BAAPy_{10}$	0.195	37458	-202.994

	T – $NIPAM_{10}$	0.221	10880	-68.989
CD20 – $AMPSA_{15}BAAPy_{15}NIPAM_{15}$	T – $AMPSA_{15}$	0.189	107072	-242.912
	T – $BAAPy_{15}$	0.191	104866	-260.598
	T – $NIPAM_{15}$	0.203	35313	-97.410
CD22 – $AMPSA_{10}BAAPy_{10}NIPAM_{10}$	T – $AMPSA_{10}$	0.214	28426	-116.813
	T – $BAAPy_{10}$	0.217	24102	-121.607
	T – $NIPAM_{10}$	0.257	9135	-49.472
CD22 – $AMPSA_{15}BAAPy_{15}NIPAM_{15}$	T – $AMPSA_{15}$	0.200	34918	-151.578
	T – $BAAPy_{15}$	0.219	24987	-123.922
	T – $NIPAM_{15}$	0.247	10014	-53.893
CD22 – $AMPSA_{15}BAAPy_{15}NIPAM_{15}BIS_{5}$	T – $AMPSA_{15}$	0.216	43810	-172.396
	T – $BAAPy_{15}$	0.199	41162	-176.439
	T – $NIPAM_{15}$	0.247	8378	-48.472
CD22 – $AMPSA_{15}BAAPy_{15}NIPAM_{15}BIS_{10}$	T – $AMPSA_{15}$	0.219	25129	-119.309
	T – $BAAPy_{15}$	0.221	23886	-129.239
	T – $NIPAM_{15}$	0.276	9395	-54.271

Entries highlighted in blue correspond to the optimal FM concentration per template which were considered for further experimental validation.

Table S6. Comparison of H-bonds and total interaction energies in CD22 systems at a 1:15 T:FM ratio, upon the addition of 5 vs 10 BIS crosslinker molecules.

Functional monomer ratio (1 T:FM_n)	Without CL		With 5 CL molecules		With 10 CL molecules	
	H-bonds	Interaction energy [kJ/mol]	H-bonds	Interaction energy [kJ/mol]	H-bonds	Interaction energy [kJ/mol]
**$AMPSA_{15}$**	34918	-151.578	43810	-172.396	25129	-119.309
**$BAAPy_{15}$**	24987	-123.922	41162	-176.439	23886	-129.239
**$NIPAM_{15}$**	10014	-53.893	8378	-48.472	9395	-54.271

Table S7. Comparison of H-bond counts and total interaction energies in MMP9 systems at a 1:15 T:FM ratio, upon the addition of 5 vs 10 BIS crosslinker molecules.

Functional monomer ratio (1 T:FM_n)	Without CL		With 5 CL molecules		With 10 CL molecules	
	H-bonds	Interaction energy [kJ/mol]	H-bonds	Interaction energy [kJ/mol]	H-bonds	Interaction energy [kJ/mol]
**$AMPSA_{15}$**	11211	-61.671	12671	-62.217	8773	-46.274
**$BAAPy_{15}$**	11998	-64.329	12128	-64.366	8236	-44.388
**$NIPAM_{15}$**	4892	-28.902	4352	-25.494	2923	-19.219

2.2. ML algorithm development and features relevance

The integrated molecular descriptor space described in the main manuscript forms the training dataset for supervised learning, enabling the RF classifier to learn descriptor-formulation class relationships and generalize to previously unseen template sequences. Categorical features, such as ComplexID and MonomerType, preserve nominal information describing distinct T-

FM combinations and formulation characteristics within the dataset. These variables provide contextual information alongside the numerical molecular descriptors, allowing the RF model to learn relationships between formulation identity and the corresponding physicochemical descriptor space. A mapping dictionary assigns each category a unique integer label, replacing the original text-based identifiers with numerical values compatible with ML training procedures.

Experimental descriptors were incorporated into the integrated descriptor space for training purposes to capture properties of the synthesized nanoMIPs beyond those obtained from sequence analysis and MM/MD simulations. Moreover, they provide experimental evidence supporting the supervisory formulation classes (“Good”, “Bad”, “Optimal”). Rather than serving as prediction targets, these features allow the RF to learn relationships between molecular descriptors and experimentally validated nanoMIP formulation behavior. FTIR fingerprint regions ($\leq$2000 cm^{-1}) were included to represent synthesized polymer chemical signatures, whereas measured parameters such as hydrodynamic size, ζ-potential, and limit of detection (LOD) provided complementary information describing nanoMIP physicochemical characteristics and sensors performance. Experimental LOD was considered as training descriptor because it provides an experimentally validated measure of the practical analytical performance of historically synthesized nanoMIP-based sensors. In contrast to Kd, which reflects equilibrium binding affinity, LOD captures the overall effectiveness of the complete sensing platform, and was therefore considered more representative of formulation performance. Hydrodynamic size measurements acquired at elevated temperature (38 °C) were additionally included as training features to account for temperature-dependent polymer behavior (e.g., for thermo-responsive formulations containing poly(N-isopropylacrylamide) (polyNIPAM)).

The current training dataset comprises 55 historical nanoMIP formulations generated using different T-FM combinations, T:FM ratios, crosslinking conditions, and experimental characterizations, resulting in 146 unique T-FM combinations. Represented across the complete integrated descriptor space of 37 descriptors, these combinations currently correspond to 5,402 individual data points. As additional MM/MD simulations and experimentally characterized nanoMIP formulations become available, the dataset will continue to expand, enabling future refinement of the ML framework.

The RF model was trained and evaluated across all combinations of hyperparameters specified in param_grid using a 5-fold cross-validation method. Alternative models, such as artificial neural networks or SVMs, were not prioritized due to their higher data demands, complexity, and limited interpretability, which are less suited to provide domain-specific insights. The 5-fold cross-validation was chosen to balance computational efficiency and robust evaluation, as it ensures that the model is tested on diverse subsets of data while limiting the training and testing cycles. While leave-one-out cross-validation offers more exhaustive testing, it is computationally expensive and less practical for the present dataset. Moreover, compared to a simple hold-out validation, 5-fold cross-validation provides a more reliable estimate of generalization performance by reducing the impact of any single train-test split. The n_estimators indicate the number of decision trees, while the max_depth parameter represents the maximum depth of trees. For hyperparameters tuning, GridSearchCV’s exhaustive search was used to ensure optimal model configuration, balancing accuracy, reproducibility, and

transparency. While other methods like Random Search or Bayesian Optimization explore larger parameter spaces more efficiently, GridSearchCV suits this study's goals of maximizing classification accuracy while maintaining reproducibility and transparency in the optimization process.

For the selected hyperparameter configuration (highlighted with squares in Figure S2), the class labeled as 'Bad' components has almost perfect scores across all metrics, which is an indicator for an easy-to-classify group, due to the distinct features that set this class apart from the others. In contrast, the 'Good' class shows slightly lower scores, but still robust, with precision and recall values around 0.86-0.88. The 'Optimal' MIP formulation class shows strong performance, with F1-scores of 0.95-0.97, indicating effective classifications. Overall, macro- and weighted-averages are consistent within all instances, reflecting a balanced model performance across different classes. Moreover, the model's performance is consistent across different cross-validation folds (5 vs. 10 folds), reinforcing confidence in the model's stability and reliability.

For the feature relevance analysis, permutation importance measures the features' impact by shuffling them and assessing accuracy changes - greater changes indicate higher feature relevance. Both SHAP values and permutation importance were chosen due to their ability to provide comprehensive insights into the contribution of each feature. While simpler methods, like correlation analysis, could highlight linear relationships, SHAP and permutation importance are better suited for capturing the complex nonlinear interactions inherent in the dataset. These methods also align with the study's focus on interpretability and ensuring that the feature rankings reflect predictive importance while facilitating scientific interpretation.

Additionally, Recursive Feature Elimination (RFE) was used to systematically identify and rank the most critical features. This approach aligns with the study's goal of prioritizing features with both strong predictive power and scientific relevance. Briefly, RFE iteratively eliminates the least important features and derives a ranking based on the order of feature elimination. In this analysis, we defined the *n_features_to_select* = 6.

As a result, the selected features with highest impact on predictions according to RFE include *T-FMTotalInteractionEnergy*, *ComplexGyrationValue*, *T-FMMinimumDistance*, *T-FMH-bonds*, *T-FMLennard-JonesPotential*, *T-FMCoulombPotential*. We observed that at certain point there was no appreciable decline in performance, indicating that additional features do not significantly hinder the model's performance.

To further examine feature contributions, we extracted the ones that produced the largest improvement in model performance during sequential forward selection (Figure 4D, left). For instance, the obtained scores suggested that, while *T-FMMinimumDistance* is a beneficial feature, it is not as strong on its own as the *T-FMTotalInteractionEnergy*. The addition of *Crosslinking* feature to the previous ones, improved our model's prediction score to 0.9505, aligning well with the understanding that crosslinking monomers have an essential role in further enhancing the stability of the formed polymer matrix. Backward elimination (Figure 4D, right) showed that removing two features slightly improved performance (cross-validation score improved from 0.9214 to 0.9410), but further removal had no effect. As additional datapoints will be added, future analyses may refine these rankings, but core physical and chemical influences should remain dominant.

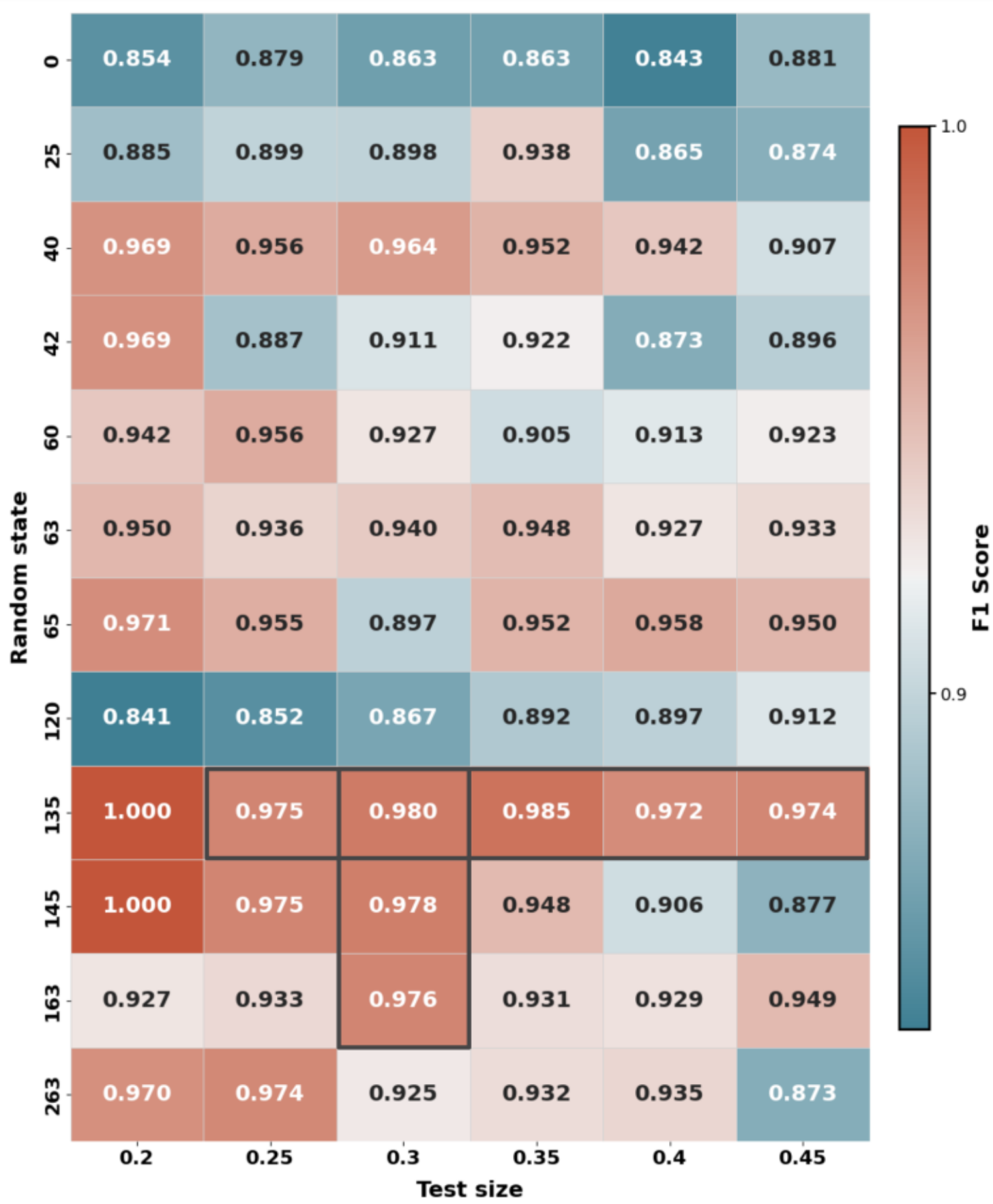

Figure S2. Heatmap of the F1-scores across class labels for different random state and test size combinations.

Following RF prediction of the formulation class, a rule-based module constructs the final nanoMIP recipe using the sequence-derived descriptors calculated from the user-provided (unseen) epitope sequence. This module considers amino acid composition (acidic, basic, neutral, polar, and hydrophobic residues), together with residue-specific descriptors for glycine, proline, and cysteine, and the chemical characteristics of the available FMs to identify chemically compatible formulations. These predefined formulation criteria are based on complementary physicochemical properties of epitopes and FMs, enabling the selection of MIP components consistent with the predicted formulation class. For instance, basic FMs are preferentially selected when the epitope contains acidic or polar residues, as indicated by non-zero acidic or polar amino acid counts, reflecting favorable complementary interactions.

The distribution analysis regarding the most relevant identified features shows that gyration variability reflects MIP properties, with short T-FM distances favoring interactions. Within the historical data, the variability in complex (T+FMs) gyration values (Figure 4E, left) reflects distinctive physical properties of the tested MIPs. T-FM minimum distances (Figure 4E, center) cluster toward lower ranges, indicating the importance of proper distances for frequent and stable interactions. Similarly, the left-skewed distribution of T-FM H-bonds (Figure 4F, left), with a steep drop-off, shows interactions involving fewer H-bonds, while larger H-bond numbers indicate ‘Good’ or ‘Optimal’ classes. Lastly, the distribution of interaction energies

(Figure 4F, center) reveals less negative values for unfavorable classes, and more negative values for the most favorable ones. As expected, consistent with the predefined formulation criteria, the distribution for 'Optimal' FMs generally covers lower (less negative) interaction energy values, when compared to the 'Good' FMs (Figure 4E, right). Moreover, while T:FM ratios indeed affect polymerization and imprinting efficiency, similar ratios can yield both 'Good' and 'Optimal' MIP formulations.

Monomers labeled as ‘Good’ generally show stronger binding affinities and interaction energies (Figure 4F, right), as expected. However, binding affinities from docking studies are limited by reduced conformational space and oversimplified environments. Hence the upper scatter for 'Optimal' FM classes indicates that, while some correlate with good binding affinities, their time-dependent interaction energies within solvated and equilibrated MD systems may not always be optimal.

The ratios between MIP components for a single T molecule correlate with the number of functional moieties in the amino acid sequences. Hence, our function selects FMs (acidic/basic/neutral) based on T’s amino acid types, count, peptide length, and performance range. The latter parameter, being an internal algorithmic threshold, enables the algorithm to recommend alternative formulations meeting the specified performance criteria rather than restricting the output to a single formulation.

To avoid repeatedly recommending identical formulations when multiple candidates satisfy the predefined criteria, the filtered dataframe is randomly shuffled before selecting the final formulation. This introduces controlled variability while avoiding systematic ordering bias and allowing alternative chemically compatible formulations to be proposed.

The logic behind crosslinker predictions is to filter the dataset to include only rows where *Crosslinking* is set to 1; value for TRUE. Taking into account that some FMs present in our training dataset may also act as crosslinking agents, e.g., 2,6-bis-acrylamidopyridine (BAAPy), and N,O-bismetacryloyl ethanolamine (NOBE), the selection criteria for crosslinking ratio is currently based on *T-FMTotalInteractionEnergy* feature. Our reasoning for this decision lies, first of all, in the provided simplicity behind the selection process. Secondly, since historical MM/MD analyses further demonstrated that additional crosslinkers may hinder proper T-FM interactions, *T-FMTotalInteractionEnergy* was the only quantity able to clearly identify these undesired scenarios.

Finally, we conducted a correlation analysis (Figure S3) to better understand the relationships among the most relevant molecular, structural, and energetic descriptors included in the integrated descriptor space.

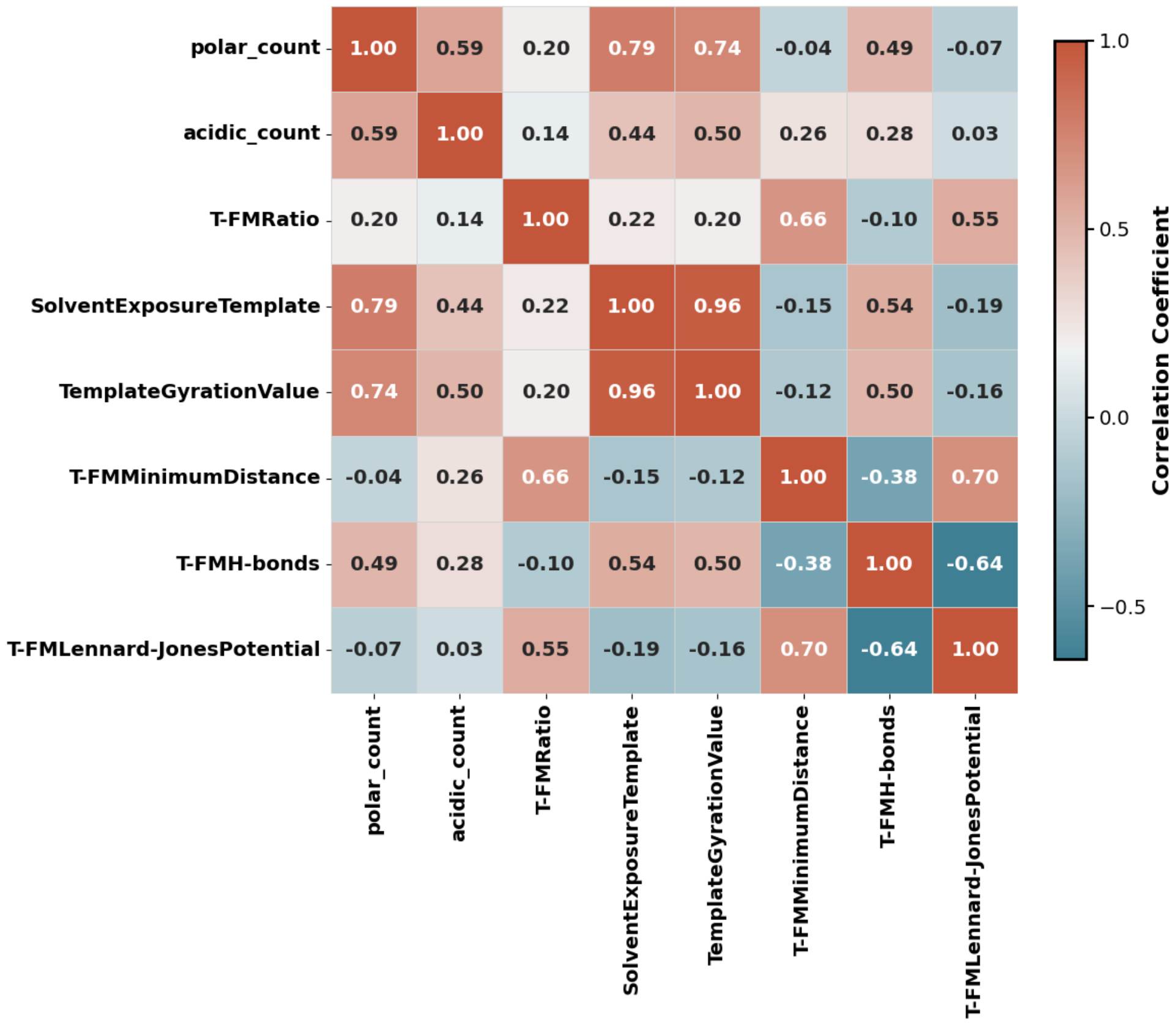

Figure S3. Pearson's correlation matrix between structural and physicochemical features of the resulting ML-based nanoMIP recipes for the unseen T sequences (cancer biomarkers).

As a result, strong positive correlations (r=0.79 and r=0.74) between the number of polar amino acids in the T sequences and the T's solvent exposure and gyration profiles were observed, respectively. Expectedly, the number of formed H-bonds between T and FMs shows moderate positive correlations (r=0.54 and r=0.50) with an increase in both T's solvent exposure and its gyration radius, respectively. Similar trends were observed for high prevalence of acidic amino acids within the T sequences, as charge repulsion between acidic amino acids forces the template structures to adopt more extended conformations (r=0.50), making them slightly better exposed to solvent (r=0.44).

We also examined how T-FM ratios influence pre-complexation kinetics. Higher ratios can influence spatial arrangements, increasing van der Waals forces (in magnitude) and steric hindrance, as shown in Figure S3.

The observed negative correlation (r=-0.64) between H-bond formation and the magnitude of Lennard-Jones potentials is consistent with increased steric hindrance, whereby closer packing may hinder the optimal donor-acceptor distance and angle required for H-bond formation.

Both *T-FMLennard-JonesPotential* and *T-FMCoulombicPotential* features correlated strongly in magnitude (r=0.68), confirming that both types of potentials, as they co-occur, are strongly influenced by the spatial arrangement of atoms within T-FMs complexes (data not shown).

These results indicate that, while descriptors showed expected physicochemical correlations, the integrated descriptor space retains complementary molecular, structural, and energetic data without excessive redundancy. Therefore, no additional dimensionality reduction was considered necessary.

2.3. MD vs ML nanoMIP recipes for cancer biomarkers detection

The Ts' amino acid sequences and the corresponding synthesis recipes used for generating nanoMIPs targeting cancer biomarkers are illustrated in Table S8.

Table S8. Amino acid sequences, recipes, and characterization (DLS, PDI, ζ-potential) of the synthesized MM/MD and ML-based nanoMIPs targeting CD20, CD22, BRCA1, BRCA2 and β2M cancer biomarkers.

	**NanoMIP recipes (**)**	**T's amino acid sequence** (from N- to C-terminal)	**Secondary structural components of T** (from N- to C-terminal)	**Hydrodynamic size** (nm)	**Polydispersity (PDI)**	**ζ-potential** (mV)
CD20 nanoMIPs_MM/MD	AMPSA-BAAPy-NIPAM, 1:15	HFLKMESLNFIRAHTPYINI	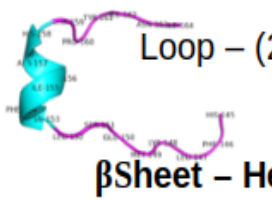 Loop – (2 turns) Helix – Loop	198	0,288	-32,2
CD20 nanoMIPs_ML	AMPSA 1:4, BAAPy 1:1, NIPAM 1:15			170	0,285	-41,3
CD22 nanoMIPs_MM/MD	AMPSA-BAAPy-NIPAM, 1:15, + 5 BIS	TLNLREVTKDQSGKYCCQVS	**βSheet – Helix – Loop – βSheet** 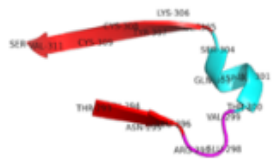	361	0,621	-44,4
* **CD22 nanoMIPs_ML**	**AMPSA 1:3, APMA 1:2, NOBE 1:15**			198	0,294	-23,2
BRCA1 nanoMIPs_MM/MD	AMPSA-BAAPy-NIPAM, 1:15	NMPTDQLEWMVQLCGA	Loop – (4 turns) Helix – Loop 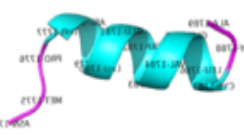	489	0,315	-35,9
BRCA1 nanoMIPs_ML	AMPSA 1:0, BAAPy 1:2, NIPAM 1:14			420	0,292	-40,5
BRCA2 nanoMIPs_MM/MD	AMPSA-BAAPy-NIPAM, 1:10	FHDPRPFPLPLSSLFSD	Loop 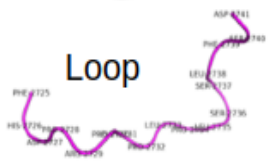	230	0,278	-24,8
BRCA2 nanoMIPs_ML	AMPSA 1:2, BAAPy 1:2, NIPAM 1:13			311	0,252	-35,9
β2M nanoMIPs_MM/MD	AMPSA-BAAPy-NIPAM, 1:15	NFLNCYVSGFHPSDI	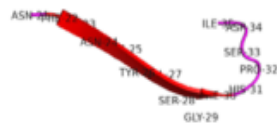 **βSheet – Loop**	361	0,487	-38,1
* **β2M nanoMIPs_ML**	**MEP 1:1, APMA 1:1, NIPAM 1:13**			489	0,421	-36,6

(*) ML output (vs MD) modifying not only the FMs' concentrations, but also the FMs type.
(**) BAAPy and NOBE also act as crosslinkers, and ML-based recipes include +5 BIS molecules.

Although the cancer biomarker epitope sequences and their MM/MD-predicted secondary structure (SS) propensities are presented in Table S1, they are reproduced in Table S8 for convenience to provide structural context for the corresponding nanoMIP formulations. In addition, Table S8 reports key physicochemical characterization parameters, including hydrodynamic diameter (DLS), polydispersity index (PDI), and ζ-potential, of both MM/MD-guided and ML-predicted nanoMIP formulations.

2.4. NanoMIP clustering, pair-wise analyses and descriptor correlations

CD22 nanoMIPs exhibited the lowest performance, although still within acceptable ranges, with R^2 values for epitope rebinding linear fit ~0.86-0.87. Additionally, these nanoMIPs correlated with higher PDI profiles. As a potential correlation with the MD-derived physicochemical properties of the CD22 sequence, its strong hydrophilicity (-0.68 GRAVY score) may decrease the potential interactions with e.g., neutral, less polar FMs such as NIPAM. Therefore, ML algorithm replaced NIPAM monomer with NOBE, which increased specific, directional H-bond T-FM interactions with polar amino acid residues, potentially contributing to more uniform, better-defined binding sites. Although the ML-based CD22 nanoMIP show

significantly reduced PDI (from 0.621 in MD to 0.294 in ML) and hydrodynamic diameters (from 361 nm in MD to 198 nm in ML), it did not substantially affect the particles rebinding capability to the T molecule.

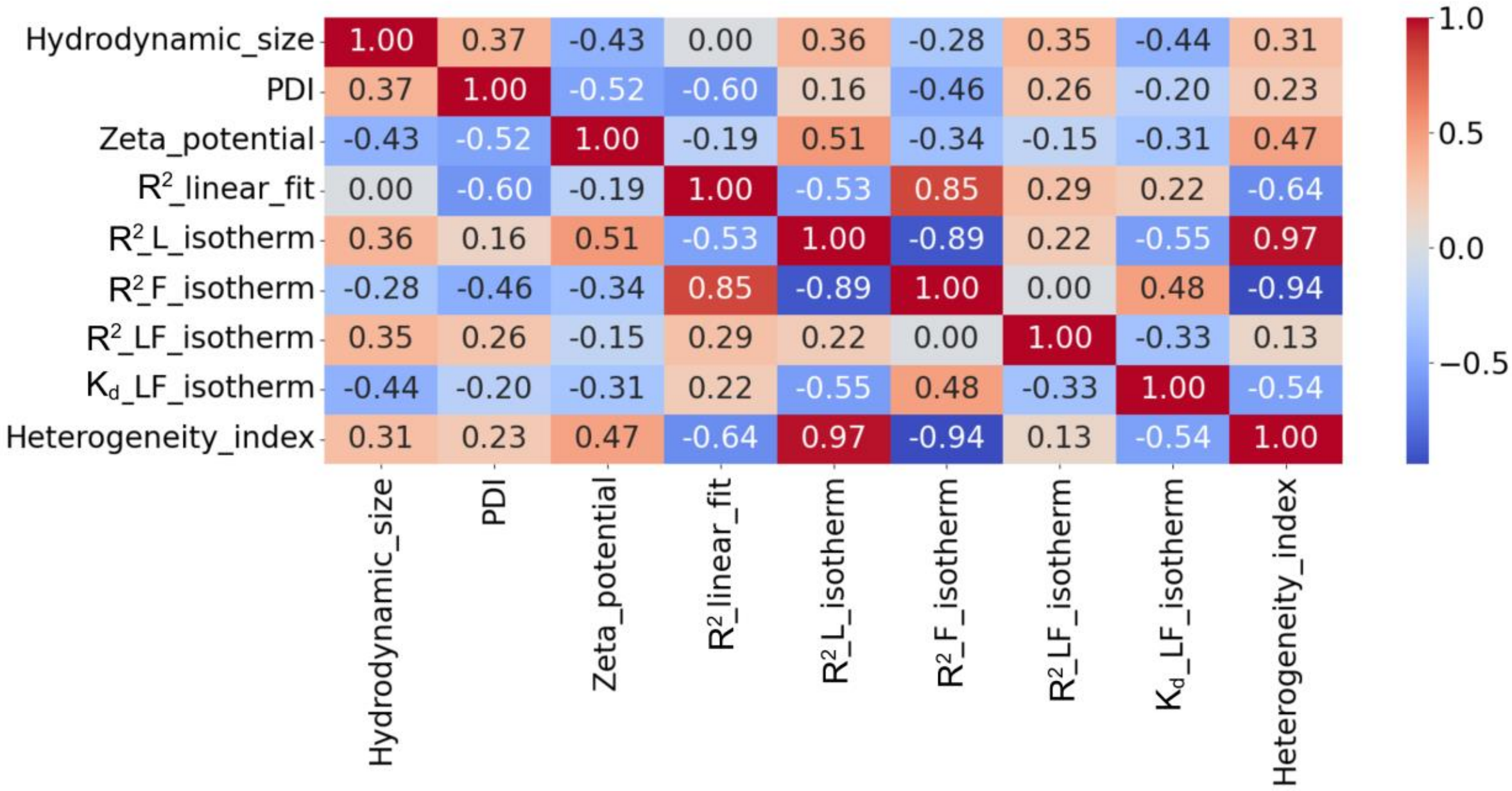

Figure S4. Correlation analysis between the experimental descriptors of the synthesized nanoMIPs. Pearson correlation coefficients were calculated to identify pairwise relationships between numerical variables. For exploratory purposes, group comparisons across different nanoMIP formulations were explored using both parametric (one-way ANOVA) and non-parametric (Kruskal-Wallis) tests. Comparison of both MM/MD- and ML-derived formulations was further assessed using Welch's t-test (two-sided, unequal variance).

2.5. Affinity studies toward epitopes and correlations between nanoMIP descriptors

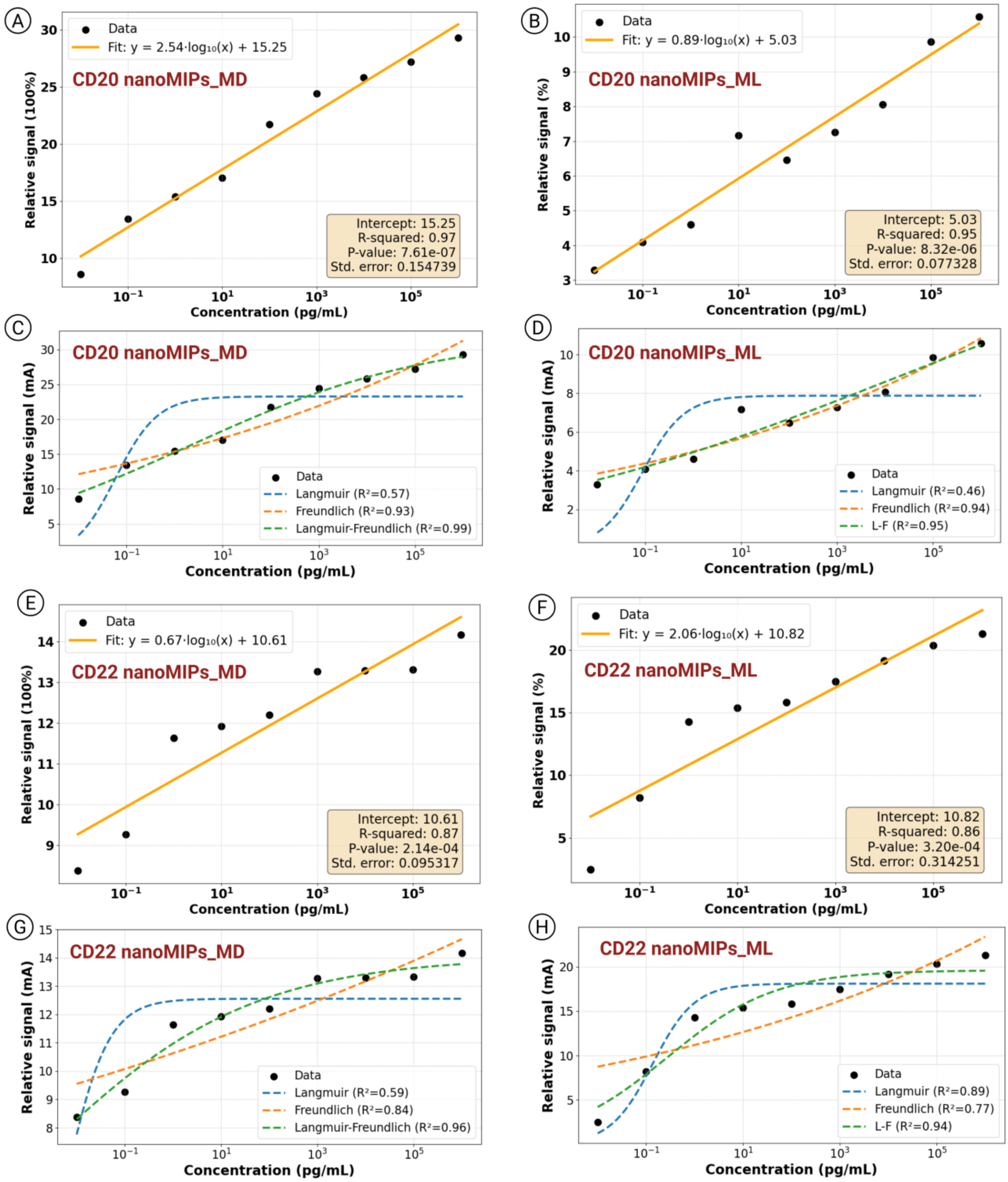

Figure S5. Linear (A, B, E, F) and isotherm fittings (C, D, G, H) applied to the electrochemical sensor measurement data for CD20 and CD22 nanoMIPs (MD and ML).

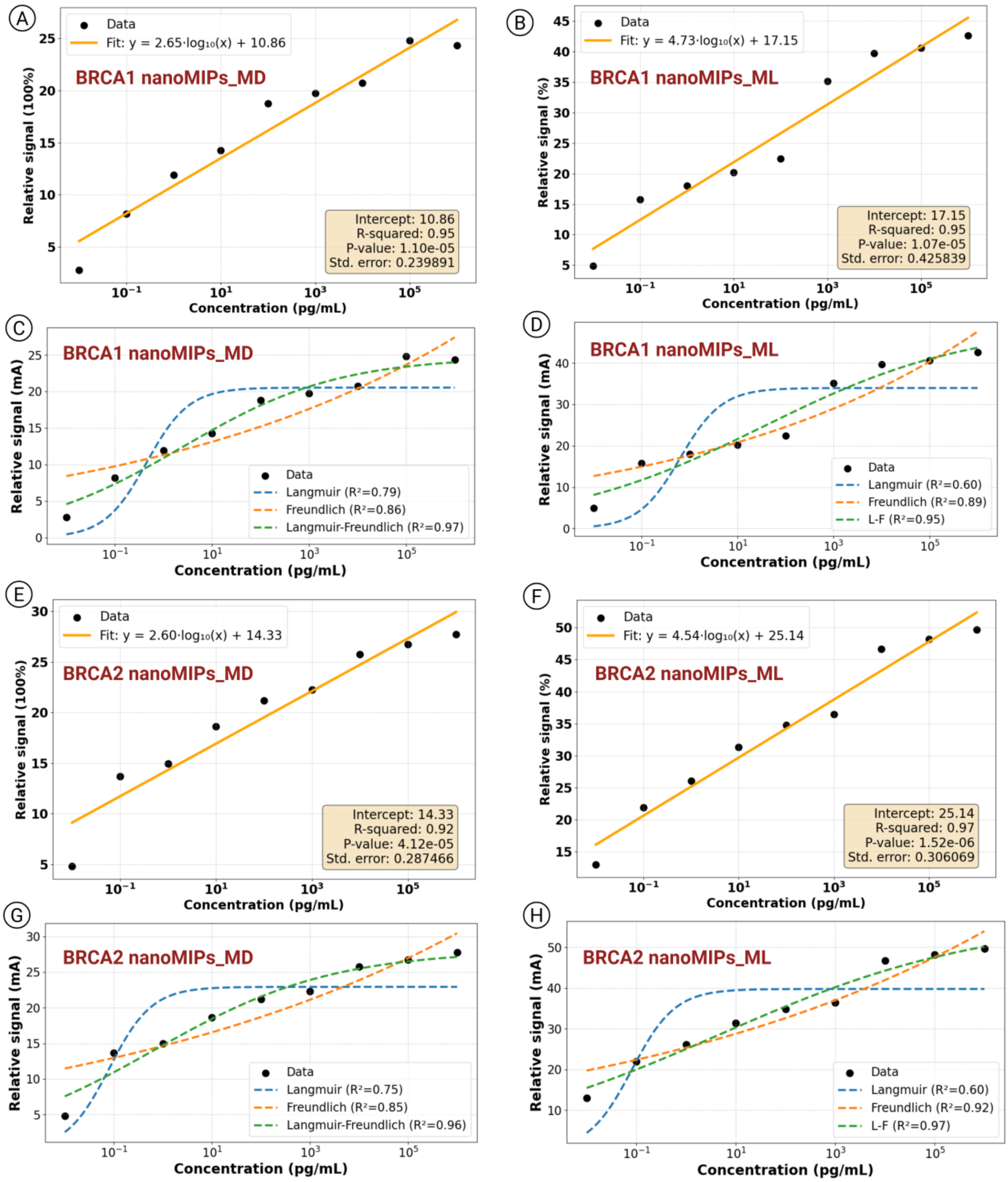

Figure S6. Linear (A, B, E, F) and isotherm fittings (C, D, G, H) applied to the electrochemical sensor measurement data for BRCA1 and BRCA2 nanoMIPs (MD and ML).

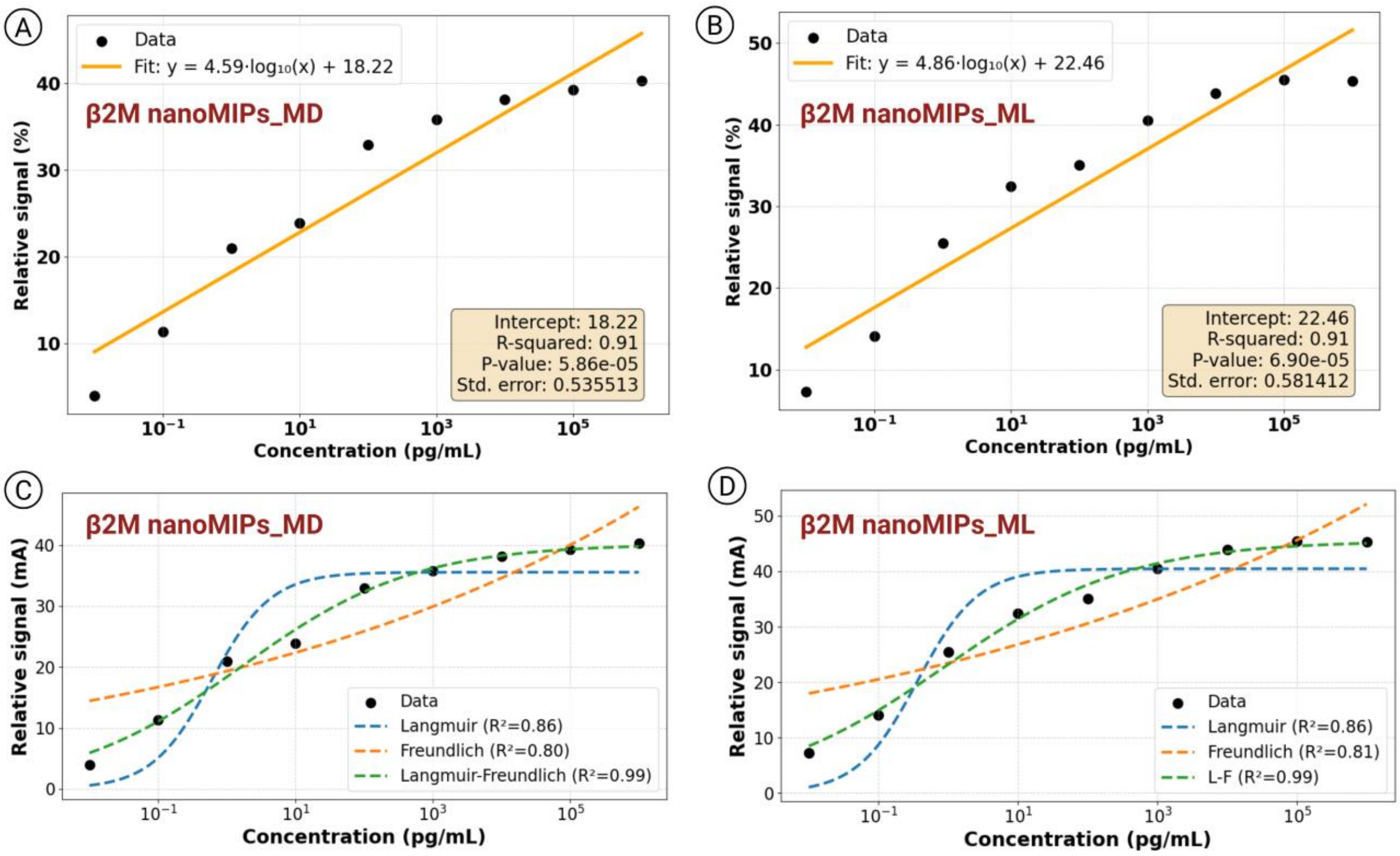

Figure S7. Linear (A, B) and isotherm fittings (C, D) applied to the electrochemical sensor measurement data for β2M nanoMIPs (MD and ML).

2.6. Cancer protein biomarker detection and specificity studies

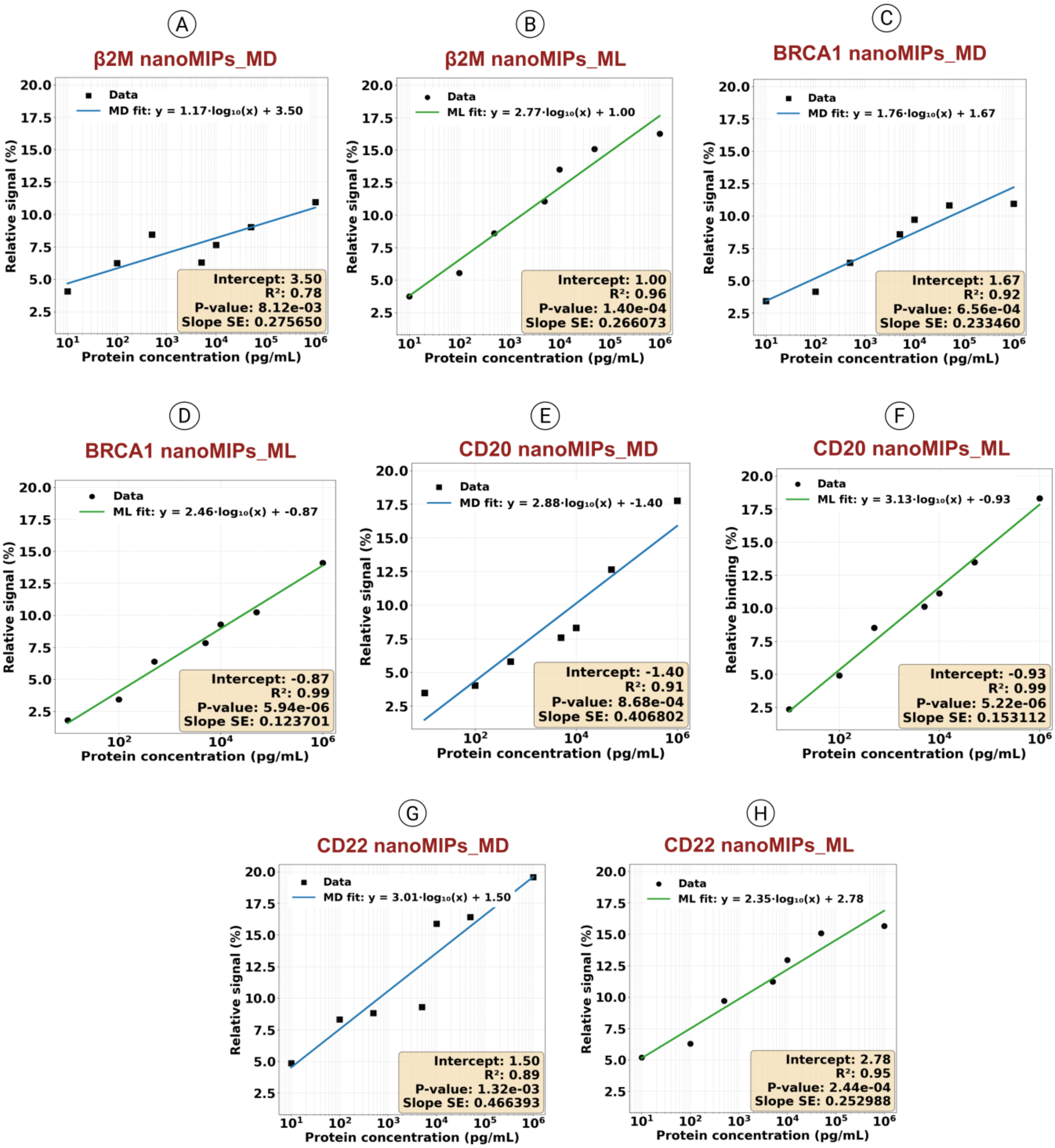

Figure S8. Linear behavior of the protein affinity assays across CD20, CD22, BRCA1, and β2M nanoMIP formulations: MD (A, C, E, G) vs ML (B, D, F, H). The concentration range of the target analytes is: 10 pg/mL, 100 pg/mL, 500 pg/mL, 5000 pg/mL, 10000 pg/mL, 50000 pg/mL, and 1000000 pg/mL.

Figure S9 illustrates the obtained relative binding signals for each nanoMIP (MD vs ML) upon both affinity (Figure S9A) and specificity (Figure S9B) studies conducted via electrochemical assays.

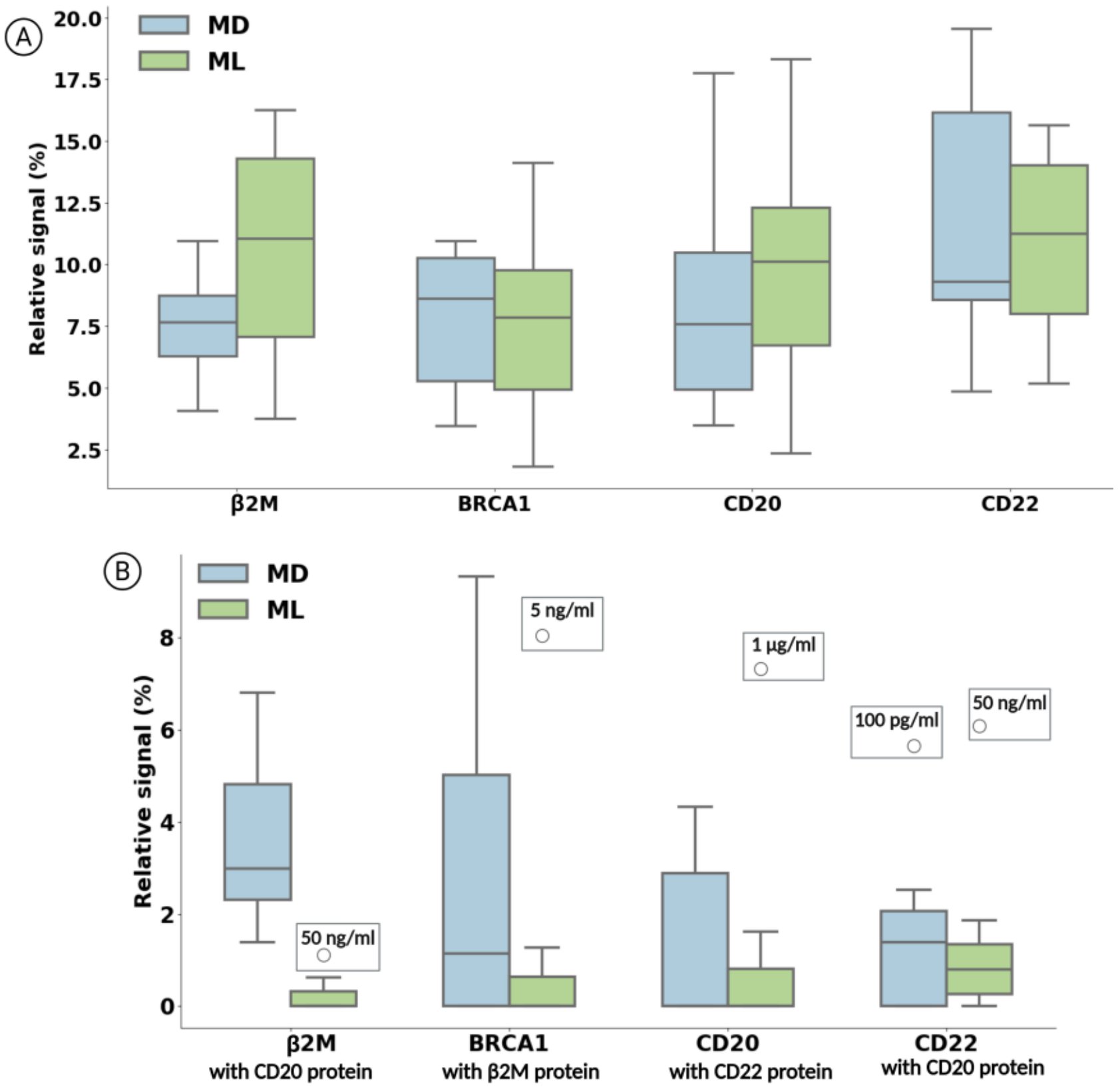

Figure S9. Relative signals associated with MD- and ML-derived nanoMIP binding to their target proteins (A), and the relative signal reduction observed during binding to cross-reactants (B). Outliers (indicated by square markers) correspond to higher analyte concentrations. The x-axis specifies the cross-reactant tested (in this batch) for each nanoMIP formulation.

To assess the batch-to-batch reproducibility for protein affinity and nanoMIP specificity using electrochemical detection, new assays were performed on different, independently prepared SPGEs (Figure S10). Overall, consistent protein binding trends were obtained across both electrode sets (SPGE production batch 1 and SPGE production batch 2), with target protein concentration-dependent responses (Figure S10A). While differences in relative signal intensity were observed for certain nanoMIPs, including a few statistically significant changes, these may be attributed to inherent variations in SPGEs' surface properties and the drop-casting process. Nevertheless, these variations did not affect the relative binding behavior of nanoMIPs, confirming the robustness and reproducibility of both nanoMIP formulation strategies.

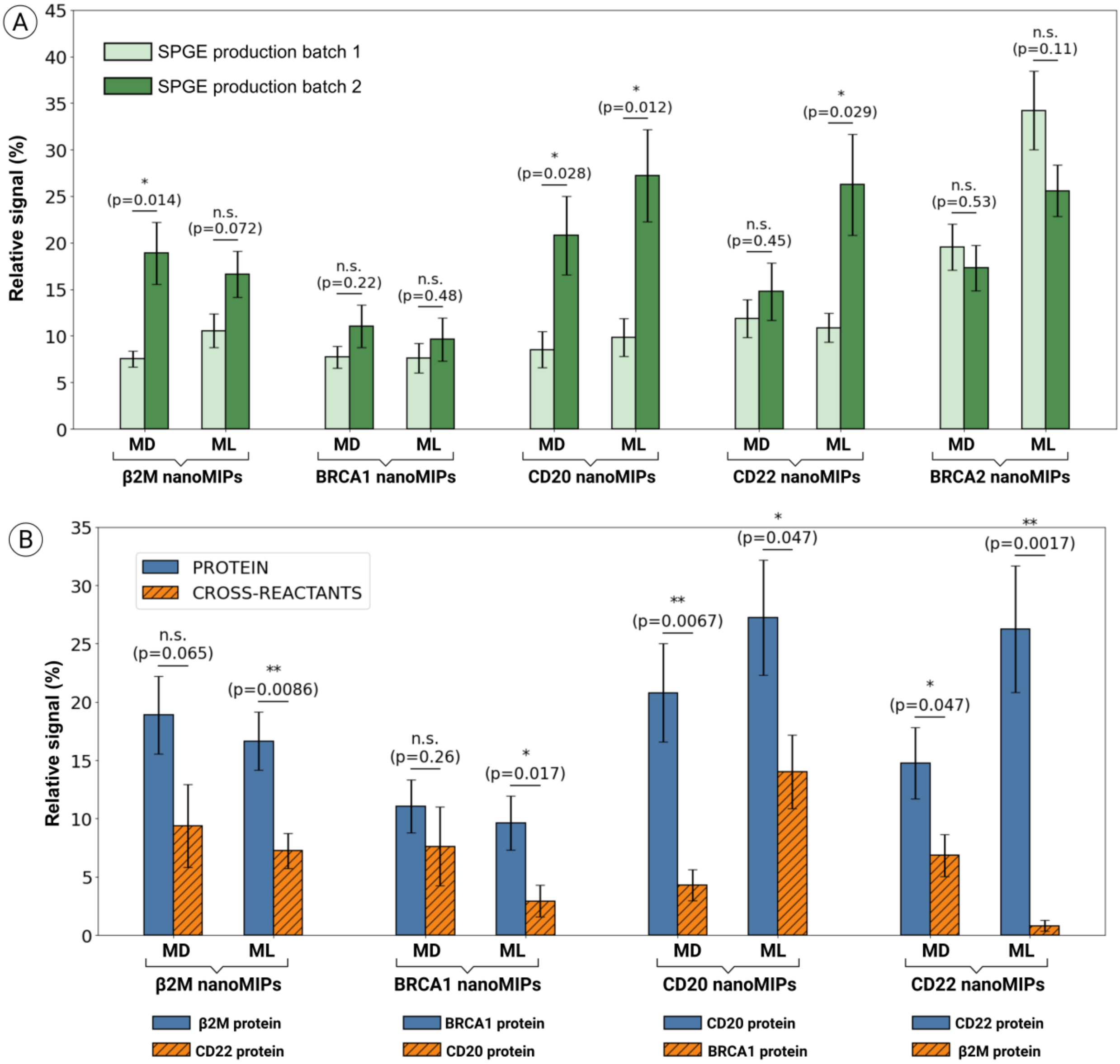

Figure S10. Reproducibility assessment via protein detection assays of MD- vs ML-nanoMIPs, comparing measurements obtained on two independent SPGEs and nanoMIP synthesis batches **(A)**. Bars represent mean signal ± SEM (Each data point represents the mean of three technical replicates (n=3). The total sample size was n=7 per nanoMIP, except for the BRCA2 nanoMIP targeting its epitope, for which n=11). Cross-reactivity analysis showing binding responses toward target proteins (data from SPGE production batch 2) vs cross-reactants (B). For visualization, negative values were clipped to zero prior to averaging, while statistical analyses were performed on raw data. Statistical significance between protein and cross-reactant binding was evaluated using two-sided Welch's t-test (unequal variance) applied to the individual raw data points across concentrations (n = 7 total data points per nanoMIP). Significance levels are indicated as follows: *p < 0.05, **p < 0.01; n.s., not significant.

Figure S10B further evaluates the nanoMIPs specificity through cross-reactivity assays using distinct cross-reactants. In all cases, nanoMIPs exhibit higher binding signals towards the

intended target analytes (SPGE production batch 2 is used), compared to cross-reactants, with most of nanoMIP systems - and all ML-based ones - reaching statistical significance ($p<0.05$).

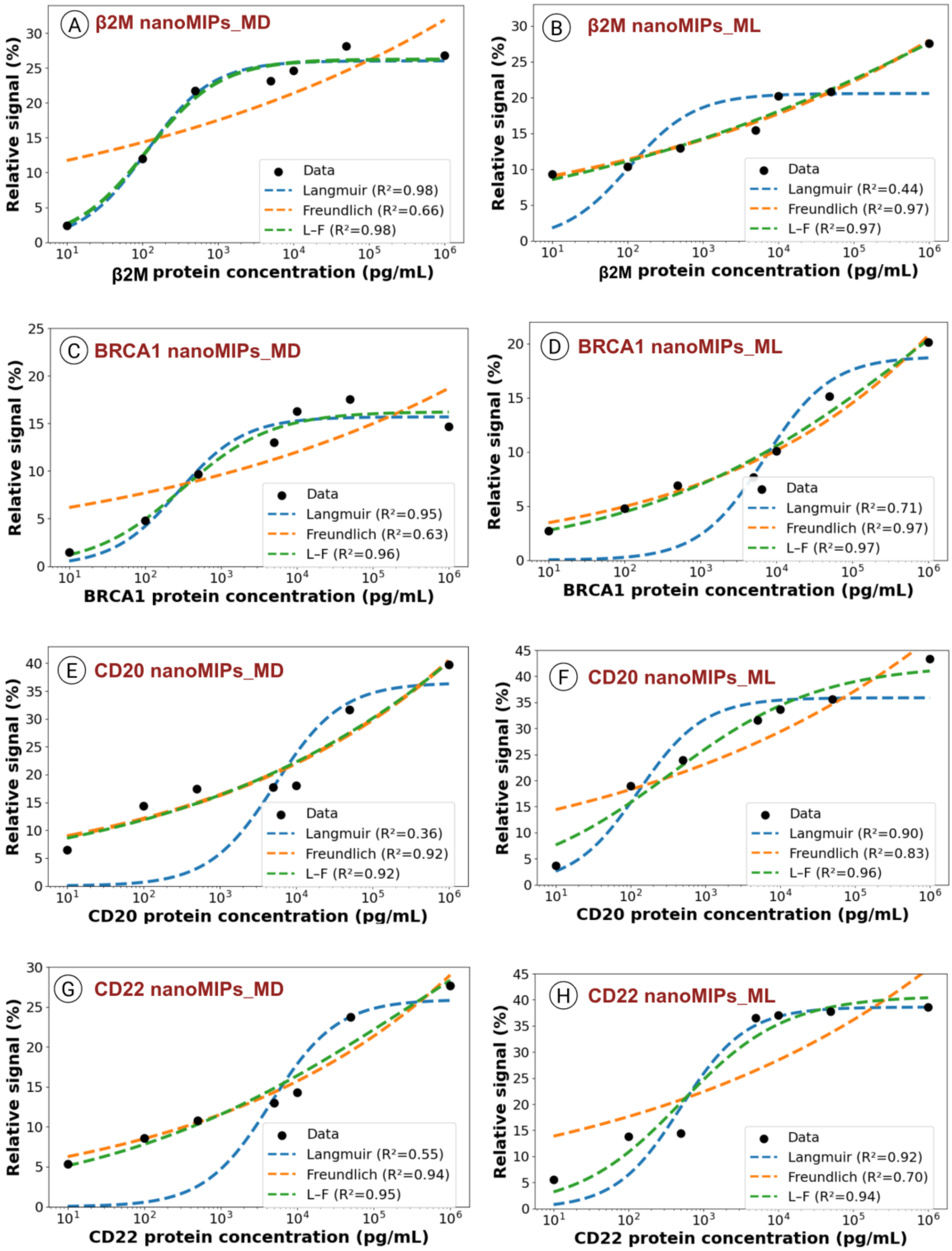

Figure S11. Binding isotherms (Langmuir, Freundlich, and Langmuir-Freundlich) fitting of the protein affinity data obtained on distinct SPGE (set 2; for reproducibility assessment) across nanoMIP formulations: MM/MD (A, C, E, G) and ML (B, D, F, H).

Moreover, the electrochemical data for protein affinity (on SPGE production batch 2) fitted to the binding isotherms (Figure S11) indicate, as expected from assays performed on SPGE production batch 1, that the underlying binding mechanism - with highest R^2 for Langmuir-Freundlich model, is preserved for both MD- and ML-derived nanoMIPs. The apparent and sporadic improvement in Langmuir fitting observed in the new SPGE data may be attributed to differences in electrode surfaces rather than changes in the intrinsic binding sites distribution of the nanoMIPs.

Taken together, Figure S10 and Figure S11 demonstrate reproducible and selective target biomarker recognition across independent electrode surfaces.

2.7. SPR-based affinity studies against cancer protein biomarkers

Table S9. Resulting equilibrium dissociation constant (**K_d**), association rate constant (k_a), dissociation rate constant (k_d), and maximum analyte binding capacity (R_{max}), and fitting parameters χ^2 and U-values upon affinity studies for MD vs ML-based nanoMIPs.

nanoMIP	K_d (M)	k_a ($M^{-1}s^{-1}$)	k_d (s^{-1})	R_{max} (RU)	χ^2	U-value
CD20 MD	2.47×10^{-12}	8.24×10^{5}	1.99×10^{-6}	248.4	29.8	3
CD20 ML	3.27×10^{-10}	8.05×10^{5}	2.64×10^{-4}	239.4	4.45	1
CD22 MD	9.93×10^{-11}	7.45×10^{6}	7.40×10^{-4}	857.0	41.6	3
CD22 ML	1.10×10^{-13}	4.70×10^{6}	5.18×10^{-7}	251.4	4.07	7
BRCA1 MD	1.49×10^{-13}	1.39×10^{7}	2.06×10^{-6}	868.0	621	2
BRCA1 ML	5.75×10^{-11}	1.59×10^{7}	2.06×10^{-4}	305.0	43.7	2
β2M MD	2.12×10^{-12}	7.20×10^{6}	1.52×10^{-5}	56.0	2.71	95
β2M ML	1.87×10^{-10}	5.24×10^{7}	9.80×10^{-3}	89.9	2.95	73

Reliable parameter estimation was achieved for most sensorgrams; however, the β2M nanoMIPs exhibited reduced parameter uniqueness. Consequently, the corresponding kinetic parameters, including the Kd values, should be regarded as approximate estimates for comparative purposes only. This reduced parameter identifiability likely reflects the complexity of the nanoMIP-target interactions and the relatively large hydrodynamic size of the β2M nanoMIPs relative to the SPR evanescent field penetration depth. χ^2 values were within acceptable limits for all sensograms except the BRCA1 MD nanoMIP.

2.9 Circular Dichroism (CD) measurements of isolated epitopes

The CD experiments (Figure S12) of the isolated epitopes in PBS (pH 7.4 at RT - same buffer recipe used during the nanoMIPs synthesis process) were included to characterize the isolated peptides of interest. Expectedly, the results indicate that all peptides adopt predominantly disordered and/or random-coil configurations (characteristic minima around 200 nm), and weaker contributions from extended structures (210-200 nm).

The observed differences between CD results and MD analysis of the peptides structural stability stem from several factors. First, in the simulated molecular environments, CHARMM force field captures short-lived local conformations and transient secondary structures during the simulation trajectory. Second, the absence of other binding partners and/or structural constraints induces structural plasticity that differ significantly from both protein/epitope-bound states and the imprinting environment. This behavior is consistent with the well-documented tendency of short fragments to exhibit intrinsic disorder in solution.[7,8] Third,

during nanoMIP synthesis (the solid-phase method used in this study), epitopes are covalently immobilized via spacer arms anchoring onto a solid support, which further restricts conformational freedom and promotes more defined structural arrangements, compared to free peptides in solution. Hence, it is important to note that CD measurements do not replicate the imprinting process, where the covalent immobilization of peptides and the presence of functional monomers and cross-linkers further influence epitope conformation.

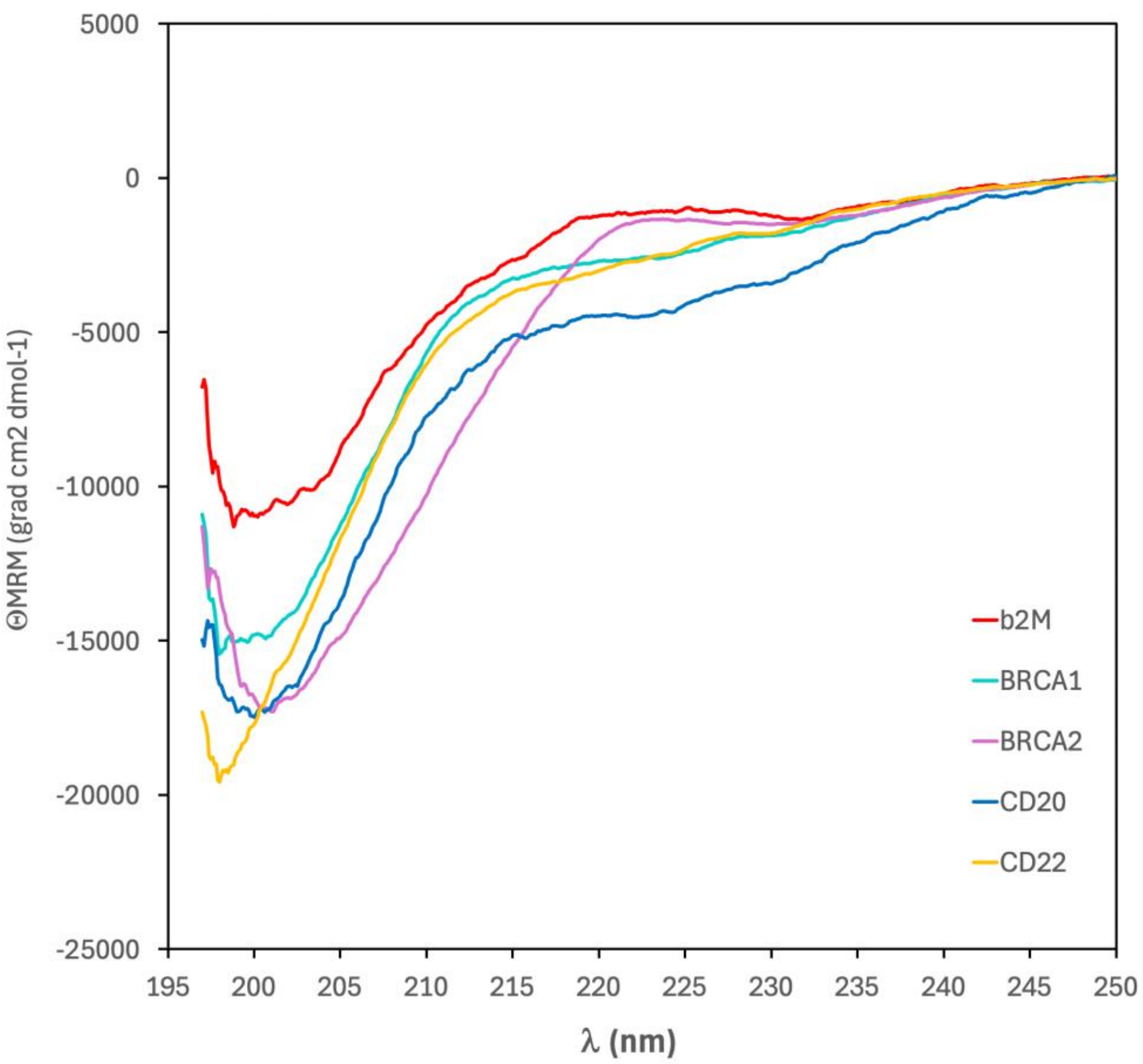

Figure S12. CD spectra of the isolated epitopes in PBS (pH 7.4, RT). Measurements were performed under diluted conditions: 0.11 mg/mL for β2M, 0.21 mg/mL for BRCA1, 0.20 mg/ml for BRCA2, 0.09 mg/mL for CD20 and 0.19 mg/mL for CD22, respectively.

4. Methods

Molecular docking and molecular dynamics (MD)

The main software used for visualization and modeling refinement purposes were PyMol[9] and Chimera[10]. For FMs modeling and initial structure optimizations, Universal Force Field (UFF)[11], the Avogadro software[12] was used. Within the docking framework (Figure S13, steps 1-4), search grids were defined to effectively encompass peptide molecules, ensuring comprehensive exploration of FMs' potential binding sites. The best docked FM conformers were selected based on binding affinity scores, RMSD values, and number of H-bonds formed between monomers and each peptide.

The large variety of small molecules, such as FMs, prevents the direct application of parameters developed for protein structures. Hence FMs were parameterized using CHARMM General Force Field (CGenFF)[13] external force field, highly compatible with CHARMM6.[14]

The employed CHARMM36 framework uses core parameters as depicted in Equation below:

$$PE = \sum k_b (b - b_o)^2 + \sum_{ang \leq k_\theta (\theta - \theta_o)^2 + \sum_{dihedral} k_\varphi [1 + cos(n\varphi - \delta)] + \sum_{L-J} 4\varepsilon_{ij} \left[\left(\frac{\sigma_{ij}}{r_{ij}} \right)^{12} - \left(\frac{\sigma_{ij}}{r_{ij}} \right)^{6} \right] + \sum_{Coulomb} \frac{q_i q_j}{4\pi \varepsilon_o r_{ij}}}$$

where k_b is the force constant of the bond, b is the bond length, b_o is equilibrium bond length, k_θ is the force constant of the angle, θ is the bond angle, θ_o equilibrium bond angle, k_φ is dihedral force constant, n is periodicity, φ is dihedral angle, δ is the phase angle, ε_{ij} is the depth of the potential energy well, σ_{ij} is the finite distance at which interatomic potential is zero, q_i and q_j are the charges of atoms i and j, and r_{ij} is the distance between the centers of atoms. Within the MD framework (Figure S13, step 12), all trajectories were analyzed considering the following quantities: (**i**) T-FMs minimum distances (MinDist), (**ii**) number of T-FMs H-bonds, (**iii**) solvent-accessible surface area (SASA) of the template molecules, (**iv**) radius of gyration (Rg) of the template-monomer-crosslinker complexes, and (**v**) total interaction energies, respectively.

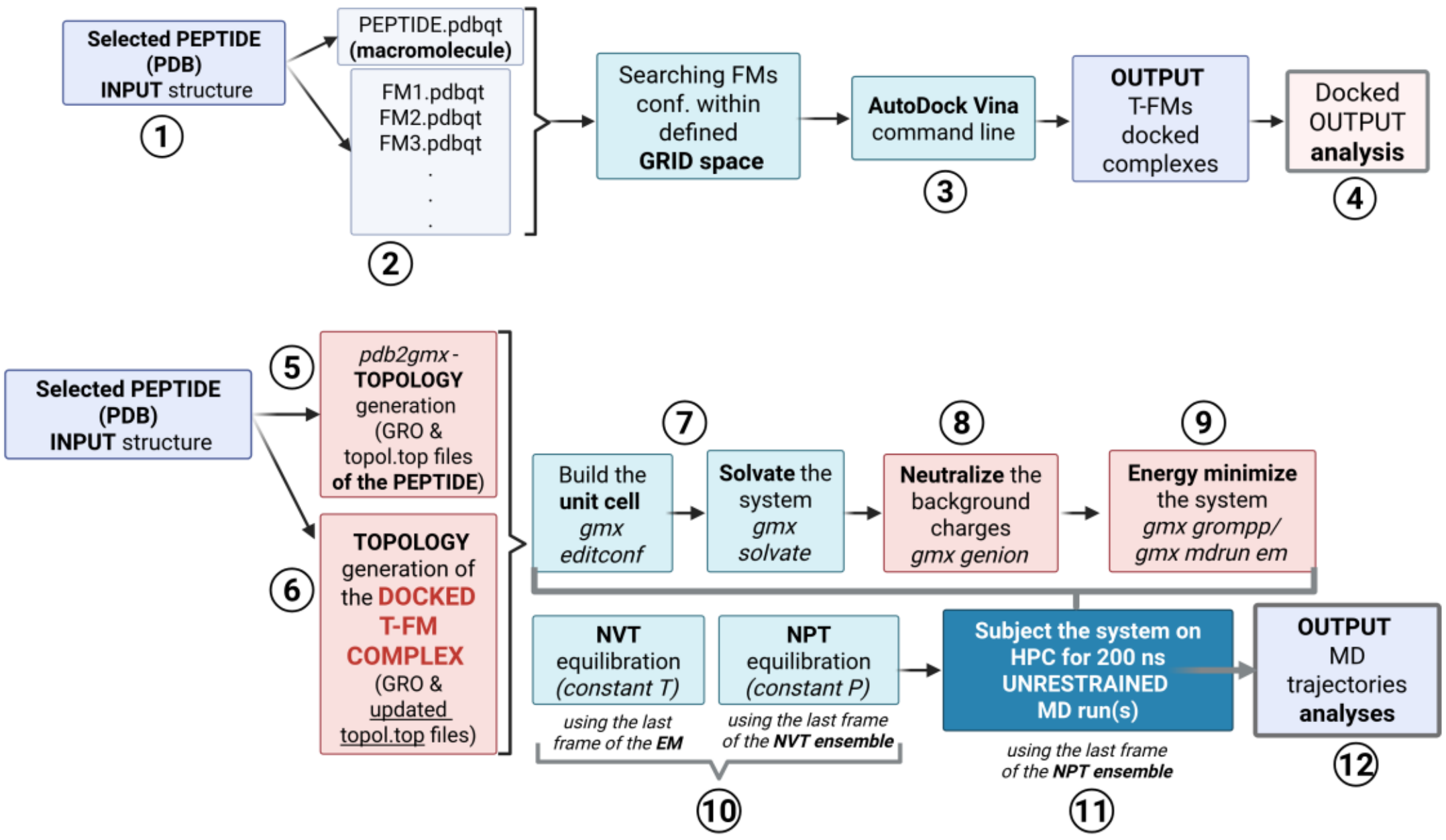

Figure S13. Computational framework used for nanoMIP design including both docking (1-4) and MD (5-12) methods.

For epitope selection studies, solvated protein biomarker systems were equilibrated under NVT/NPT ensemble for 1000 ps with mild position restraints on heavy atoms, at 298 K and 1 bar. Unconstrained MD productions were carried out for 500 ns. For the nanoMIP design targeting CD20, CD22, BRCA1, BRCA2, β2M, MMP9, and BRAF-V600E biomarkers, MD trajectories were performed covering various T and FM types, T-FMs ratios, and T-FMs±CL combinations and concentrations. Specifically, MD productions were performed on seven cancer biomarkers, including systems with lower T:FM ratios (e.g., 1:3). Additional runs containing 5, 10, and 15 FM molecules per template were performed using only the best performing five cancer biomarkers. Further screened conditions included CL molecules at ratios of 5 and 10 molecules in systems with 1:15 T:FMs ratio.

Leapfrog integrator[15] was used for all MD productions. Temperature couplings were managed using the V-rescale thermostat,[16] with separate coupling groups for the protein and/or T-FM complexes and solvent - with or without ions, each maintained at the reference temperature with a time constant of 0.1 ps. Pressure couplings were applied via Parrinello-Rahman algorithm,[17] enforcing isotropic scaling of the unit cell with a compressibility of 4.5e-5 bar^{-1}.

Additionally, LINCS algorithm[18] was used to maintain holonomic constraints, specifically on H-bonds. The need for LINCS-based constraints emerges from the fast vibrational motions of H atoms. Neighbor searching was conducted using the Verlet cutoff scheme[19] with a grid-based searching method. The short-range van der Waals interactions were calculated with a cutoff range set between 1.0-1.2 nm. Long-range electrostatics were handled using the Particle Mesh Ewald (PME) method.[20]

Analyses of MD trajectories were carried out using the Gromacs package tools, Grace plotting tool[21], and visualization packages such as PyMol[9] and VMD[22], supplemented by in-house developed Python scripts for further analyses, including statistics.

ML algorithm development

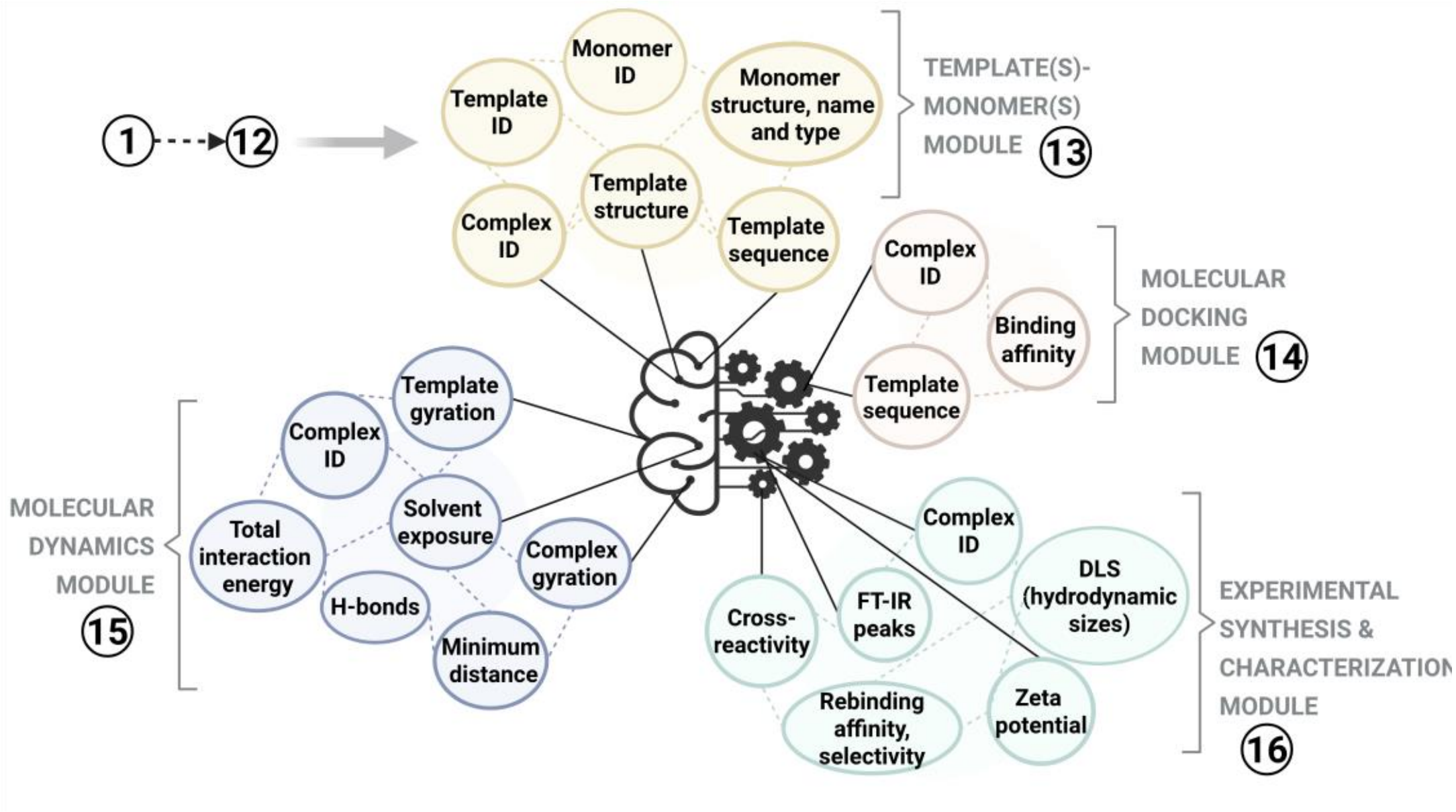

Figure S14. Input dataset features used in algorithm development. The 1-12 annotation in this figure refers to the earlier (MM/MD) steps outlined in Figure S13.

Table S10. Categorical features considered in the input dataset.

Features	Description	Module *
**ComplexID**	Merges the indices of each T-FM combination.	*T-FM Complex*
**TemplateID**	The index of each T molecule.	*Template molecule (T)*
**TemplateSequence**	The one-letter code of amino acids within each T sequence; e.g., DGTTEDANGNKVNTK.	
**TemplateSS**	Secondary structural components of each T sequence; e.g., LOOP-SHEET-LOOP-HELIX-LOOP (from the N-terminal end of each peptide).	
**MonomerID**	The index of each FM molecule.	*Functional monomer (FM)*
**MonomerName**	The common/selected name of each FM, e.g., APMA.	
**MonomerType**	The type FM of interest (acidic/basic/neutral).	
**MonomerFormula**	The chemical formula of FMs; e.g., C7H14N2O (C-carbon, H-hydrogen, N-nitrogen, O-oxygen).	
**Crosslinking**	Indicates whether the investigated recipes contain cross-linkers; e.g., TRUE for yes, FALSE for no.	*Crosslinker (CL)*
**Crosslinker**	The name of crosslinking monomer; e.g., BIS (for cases where crosslinkers were not used, the employed name is ‘MMM’ – denoting *missing*).	
**OutcomeID**	Identifies each T-FM +/- CL interaction, and indicates the FMs considered in distinctive recipes.	*Docking & MD*
**FingerprintPeak1-3**	Indicates the bending and stretching vibrations of the molecular bonds; 3 peaks extracted from FT-IR data.	*Experiment*

**LODUnit**	Measurement unit for the limit of detection of the resulted MIPs; extracted from rebinding/sensor studies.	
**OutcomeEXP**	Identifies each T-FM +/- CL interaction.	
**Outcome_availability**	Indicates the presence or absence of experimental data.	

* *The modules are not explicitly included in the dataset.*

Table S11. Numerical features considered in the input dataset.

Features	Description	Module *
**T-FMRatio**	The ratio for each T-FM combination, in decimal format; e.g., 0.142 denoting that 1 template molecule interacts with 7 FM molecules.	*T-FM Complex*
**BindingAffinity**	Resulted binding affinities between T and FMs upon molecular docking studies.	*Docking*
**SimulationTemperatureK**	Temperatures considered for MD productions (in K).	*MD*
**SimulationTimeNS**	The unconstrained MD runs duration in *ns*.	
**SolventExposureTemplate**	Indicates the average solvent accessible surface area (SASA) of the T molecule during MD runs.	
**TemplateGyrationValue**	Indicates the T's gyration behavior (averaged) during MD runs.	
**ComplexGyrationValue**	Indicates the average gyration behavior of T-FM-CL during MD runs.	
**T-FMMinimumDistance**	Indicates the average minimum distances between T and FM during MD runs.<br>(in the presence or absence of CL)	
**T-FMH-bonds**	Indicates the number of total hydrogen bonds formed between T and FM during MD runs.	
**T-FMLennard-JonesPotential**	Lennard-Jones potential energy between T and FMs.<br>(in the presence or absence of CL)	
**T-FMCoulombPotential**	Coulomb potential energy between T and FMs.<br>(in the presence or absence of CL)	
**T-FMTotalInteractionEnergy**	The sum between Lennard-Jones and Coulomb potential energies for each T-FM combination<br>(in the presence or absence of CL).	
**DLSsize25C/38C**	The hydrodynamic size of synthesized MIP nanoparticles, considered at different temperatures (°C).	*Experiment*
**ZetaPotential**	Indicates the surface charge of particles in suspension.	
**FT-IRPeak1-3**	Peaks of the molecular vibrations of the chemical bonds within MIPs; 3 peaks extracted from FT-IR data.	
**LODRebinding**	The obtained limit of detection of the resulted MIP-based sensors; extracted from rebinding/sensor studies.	
**TemperatureMIPsWash**	The temperature used for MIP collection.	

* *The modules are not explicitly included in the dataset.*

The categorical variables were encoded using *LabelEncoder* imported from scikit-learn's preprocessing module. The function used to count different types of amino acids (acidic, basic, neutral, polar and non-polar) within template sequences also included the special cases such as glycine, proline and cysteine.

After the algorithm predicts formulation classes using predefine criteria, the final recipe selection on unseen T sequences then integrates these class predictions alongside five key variables: (i) FM chemical type (acidic, basic, or neutral) and formula, (ii) target amino acid types, (iii) amino acid counts, (iv) peptide length, and (v) a flexible performance range threshold set at 0.5. This performance parameter allows a variable cutoff only for the primary selection feature, which is the total T-FM interaction energy in the presence or absence of crosslinking. Upon filtering the database according to these combined criteria and predicted class performances, the module samples one "Good" and one "Optimal" monomer, mapping them specifically to their complementary target amino acid types to calculate the final nanoMIP formulation component ratios.

For crosslinking, the crosslinker ratio is established by filtering the dataframe to identify if crosslinking conditions apply (TRUE or FALSE), depending on the presence of bifunctional monomers in the predicted formulation class and their ratios in the final recipe output.

Besides the best performing MIP formulation classes and potential alternatives, the users receive as output a classification report printing out the precision, recall, F1-score, and support metrics for each identified class, providing a comprehensive evaluation of the model's performance.

Lastly, the libraries considered in this study include NumPy[23] for numerical computations, Pandas[24] for data processing, Scikit-learn[25] for implementing the ML algorithm and evaluating the model's performance, Matplotlib[26] for data visualization, and SHAP[27] for Shapley Additive Explanations analysis.

Chemicals, reagents and instrumentation for nanoMIPs synthesis and characterization

Bare glass beads with a diameter of 0.75-1 mm, used for peptide immobilization, were purchased from Carl Roth (Karlsruhe, Germany). Functional monomers including 2-methacryloyloxyethyl phosphate (MEP), 2,6-bis(acrylamido)pyridine (BAAPy), N,O-bismethacryloyl ethanolamine (NOBE), N-(3-aminopropyl)methacrylamide hydrochloride (APMA), 2-acrylamido-2-methyl-1-propanesulfonic acid (AMPSA), N-isopropylacrylamide (NIPAM), and N,N′ methylenebisacrylamide, alongside N′-tetramethylethylenediamine (TEMED), ammonium persulphate (APS), bovine serum albumin (BSA), ethanolamine and phosphate-buffered saline (PBS) were purchased from Sigma Aldrich (Steinheim, Germany). PBS solution (10 mM) was used as buffer. The hexaferrocyanide ($K_3[Fe(CN)_6]$) for redox marker solution, ultra-pure water, methanol (MeOH), ethanol (EtOH), and syringe filters of 0.45 µm pore size were purchased from Carl Roth (Karlsruhe, Germany). Sodium hydroxide (NaOH) was purchased from VWR (Dresden, Germany), while acryloxyethyl thiocarbamoyl Rhodamine B (RhB monomer) was obtained from Polysciences Europe GmbH (Hirschberg an der Bergstrasse, Germany). All chemicals and solvents were high-performance liquid chromatography (HPLC)/analytical graded and were used without any further purification. All

peptides and proteins used in this study were purchased from GenScript Biotech (Netherlands) BV.

During synthesis, a vacuum oven (Vacutherm, Fischer Scientific GmbH, Germany) was used for drying purposes, and a bath sonicator (Bandelin Sonorex Digiplus, Eydam GmbH, Germany) for ensuring that monomers are uniformly distributed throughout the solution. The hydrodynamic size distribution and ζ-potential of nanoMIPs were characterized by using Zetasizer Pro, (Malvern Panalytical Ltd, United Kingdom) at a temperature of 25 ± 0.1°C. Additionally, MarvinSketch 23.14 software[28] from ChemAxon was used to analyze the primary structure of peptides, conduct elemental analysis and extract their molecular weights for further calculations. Fluorescent microscopy (BZ-X810 coupled with BZ-X800 Analyzer software, Keyence Corporation, Japan) was employed to take fluorescent images under 10× magnification. The nanoMIPs binding capabilities to the corresponding peptides and proteins were investigated via voltammetric measurements using a compact PalmSens4 Potentiostat with PS Trace 5.12 software (from PalmSens B.V., The Netherlands) and screen-printed gold electrodes (SPGEs) from Metrohm Deutschland GmbH & Co.

Surface functionalization

The nanoMIPs synthesis process (Figure S15) starts by weighting approximately 60 g of glass beads, as solid support for peptide immobilization. Prior to peptide immobilization, the beads were hydroxylated with 2 M NaOH via boiling(100-120°C) for 20 min. Following hydroxylation, the glass beads were washed with water until their pH reached 6.5 -6.8.

Upon 1 h drying process under vacuum, the hydroxylated beads were transferred into a round-bottom flask and subjected to 30 minutes N_2 flow. Subsequently, beads were incubated overnight with 50 mL of 2% (v/v) 3-aminopropyl)triethoxysilane (APTES) solution in anhydrous toluene. The silanized beads were washed with acetone and methanol and further functionalized with 50 mL 7% glutaraldehyde (GA) solution in PBS buffer for 2 h at RT.

Peptide immobilization

Upon GA incubation, ~10 mg of peptide dissolved in 40 ml PBS, was incubated with the functionalized glass beads overnight at RT. The beads drying involved the use of vacuum pump for 1 h.

Functional monomers (± crosslinker) polymerization

The template immobilized onto the beads was incubated for 30 min, at RT, with 35 mg of sodium borohydride ($NaBH_4$) in 35 mL PBS for Schiff-base reduction, and washed with 50 mL DI water. Afterwards the beads were incubated for 15 min at RT with 500 µL ethanolamine of 10 mM dissolved in 49.5 mL filtered PBS for blocking unoccupied sites on the beads surface. Lastly, beads were washed with DI water in multiple steps, and left to dry for 1 h under vacuum.

The dried beads and the polymerization mixtures prepared according to Table S12 were then subjected to N_2 purging for 30 min separately. Each polymerization solution was prepared in a total volume of 50 ml milli-Q water. Upon purging, the polymerization mixtures were sonicated

under vacuum 20 min. Subsequently, the polymerization solution was transferred onto the beads, under N_2 atmosphere, and complexated for 30 min at RT. The initiator solution (1% w/v) was prepared by dissolving 10.9 mg of APS and 3 µl of TEMED in a total volume of 1 ml milli-Q water, and it was then transferred into the polymerization mixture. The polymerization reaction was performed for 2 h at room temperature based on our previous works,[29-32] as a standard condition to ensure consistent comparison between MM/MD and ML-based design strategies. The low affinity particles, unreacted monomers, and oligomers were removed via a cold wash with DI water at 4°C. The high-affinity nanoMIPs were collected via sequential hot-wash elutions, leaving the beads and template molecules behind, with hot-wash temperatures ranging between 74-78 °C.

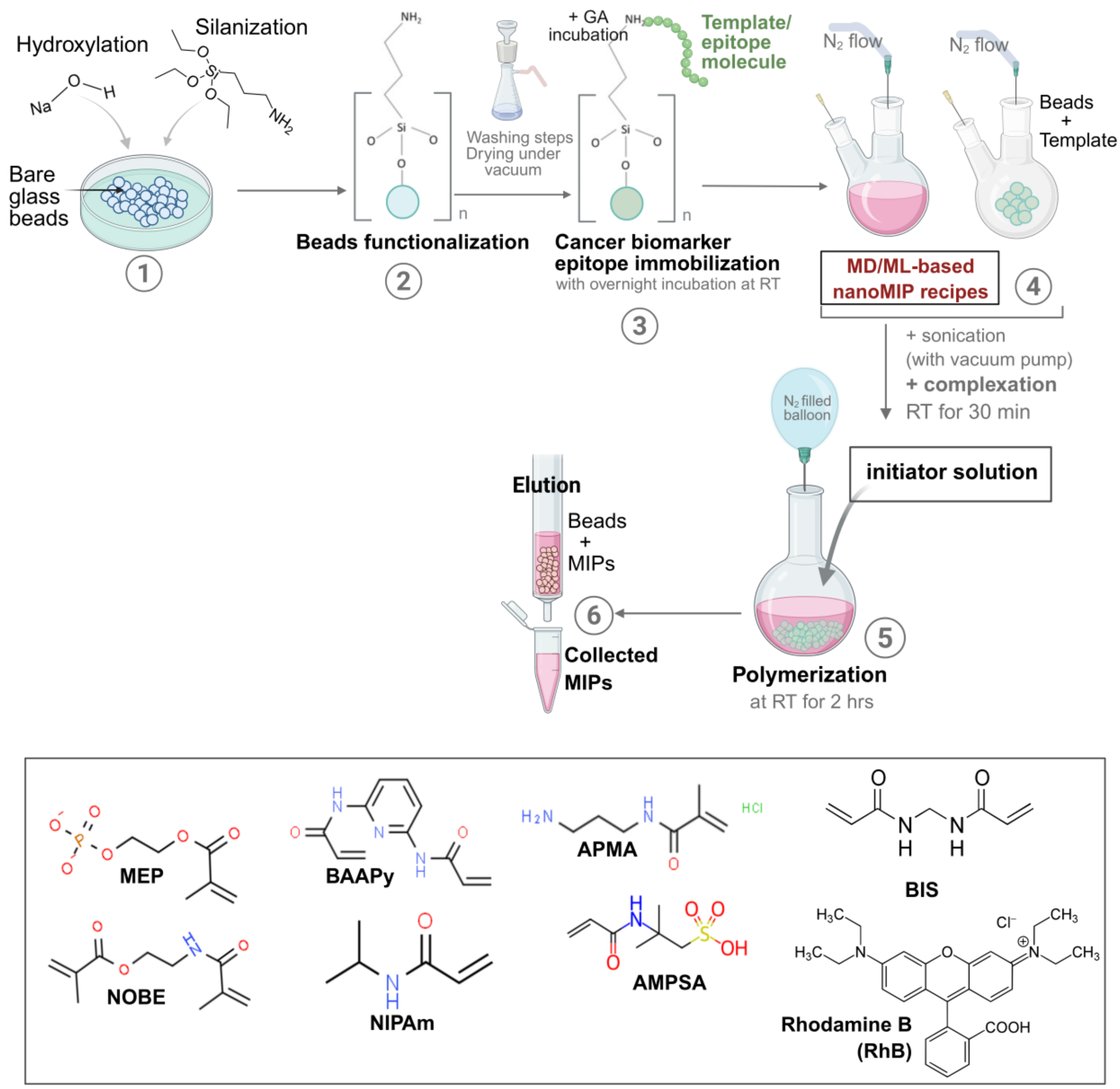

Figure S15. Solid-phase synthesis of nanoMIP recipes (steps 1-6), including the chemical structures of monomers, crosslinker and the dye monomer (RhB) used in the synthesis process.

Table S12. Polymerization mixtures of both MD and ML nanoMIPs.
This table should be considered in conjunction with Tables S1 and Table S8.

**NanoMIP formulation** ± CL	**Polymerization mixture** *in 50 ml total volume*	**Target epitope**
**AMPSA-BAAPy-NIPAM (1:15)**	AMPSA: 12.6 mg / 16.92 mg / 18.5 mg<br>BAAPy: 13.2 mg / 17.74 mg / 19 mg<br>NIPAM: 7 mg / 9.24 mg / 10 mg<br>RhB: 0.5 mg	CD20 (10.08 mg) / BRCA1 (10 mg) / β2M (10 mg)
**AMPSA-BAAPy-NIPAM (1:15)** + BIS (1:5)	AMPSA: 13.5 mg<br>BAAPy: 14.2 mg<br>NIPAM: 7.39 mg<br>BIS: 3.39 mg<br>RhB: 0.5 mg	CD22 (10.14 mg)
**AMPSA-BAAPy-NIPAM (1:10)**	AMPSA: 10.51 mg<br>BAAPy: 11.05 mg<br>NIPAM: 5.80 mg<br>RhB: 0.5 mg	BRCA2 (10 mg)
**AMPSA-BAAPy-NIPAM (1:4 - 1:1 – 1:15)** + BIS (1:5)	AMPSA: 3.5 mg<br>BAAPy: 1 mg<br>NIPAM: 6.94 mg<br>BIS: 3.15 mg<br>RhB: 0.5 mg	CD20 (10.08 mg)
**AMPSA-BAAPy-NIPAM (1:0 - 1:2 – 1:14)** + BIS (1:5)	BAAPy: 2.36 mg<br>NIPAM: 8.62 mg<br>BIS: 4.19 mg<br>RhB: 0.5 mg	BRCA1 (10 mg)
**AMPSA-BAAPy-NIPAM (1:2 - 1:2 – 1:13)** + BIS (1:5)	AMPSA: 2.11 mg<br>BAAPy: 2.21 mg<br>NIPAM: 7.46 mg<br>BIS: 3.91 mg<br>RhB: 0.5 mg	BRCA2 (10 mg)
**AMPSA-APMA-NOBE (1:3 - 1:2 – 1:15)** + BIS (1:5)	AMPSA: 2.73 mg<br>APMA: 1.57 mg<br>NOBE: 13.01 mg<br>BIS: 3.39 mg<br>RhB: 0.5 mg	CD22 (10.14 mg)
**MEP-APMA-NIPAM (1:1 - 1:1 – 1:13)** + BIS (1:5)	MEP: 1.30 mg<br>APMA: 1.10 mg<br>NIPAM: 8.6 mg<br>BIS: 5 mg<br>RhB: 0.5 mg	β2M (10 mg)

$Mw_{CD20\ peptide}$ = 2444.89 g/mol; $Mw_{CD22\ peptide}$ = 2272.58 g/mol; $Mw_{BRCA1\ peptide}$ = 1836.13 g/mol; $Mw_{BRCA2\ peptide}$ = 1972.235 g/mol; $Mw_{\beta 2M\ peptide}$ = 1712.90 g/mol.
Mw_{AMPSA} = 207.25 g/mol; Mw_{BAAPy} = 217.22 g/mol; Mw_{NIPAM} = 113.16 g/mol; Mw_{NOBE} = 197.23 g/mol; Mw_{APMA} = 178.7 g/mol; Mw_{MEP} = 210.12 g/mol; Mw_{BIS} = 154.17 g/mol.

The nanoMIP yields obtained via freeze-drying are summarized in Figure S16. NanoMIP yield was determined gravimetrically using pre-weighed glass vials. A 10 mL aliquot of nanoMIP suspension (from a total elution volume of 50 mL per formulation) was freeze-dried and the vial reweighed (n = 5). The nanoMIP mass was calculated from the difference between pre- and post-drying weights, and values were converted to µg mL^{-1}. The obtained yield was in

agreement with the literature reports.[33-35] Correlation analyses (both Pearson and Spearman) were performed to assess the potential relationship between the obtained nanoMIP yields and their hydrodynamic sizes vs SS components within epitopes used during syntheses.

NanoMIP recipe	NanoMIP concentration
CD20 nanoMIP_MD	23.4 µg/ml
CD20 nanoMIP_ML	24 µg/ml
CD22 nanoMIP_MD	26 µg/ml
CD22 nanoMIP_ML	30.4 µg/ml
BRCA1 nanoMIP_MD	63 µg/ml
BRCA1 nanoMIP_ML	60 µg/ml
BRCA2 nanoMIP_MD	40 µg/ml
BRCA2 nanoMIP_ML	128 µg/ml
β2M nanoMIP_MD	200 µg/ml
β2M nanoMIP_ML	120 µg/ml

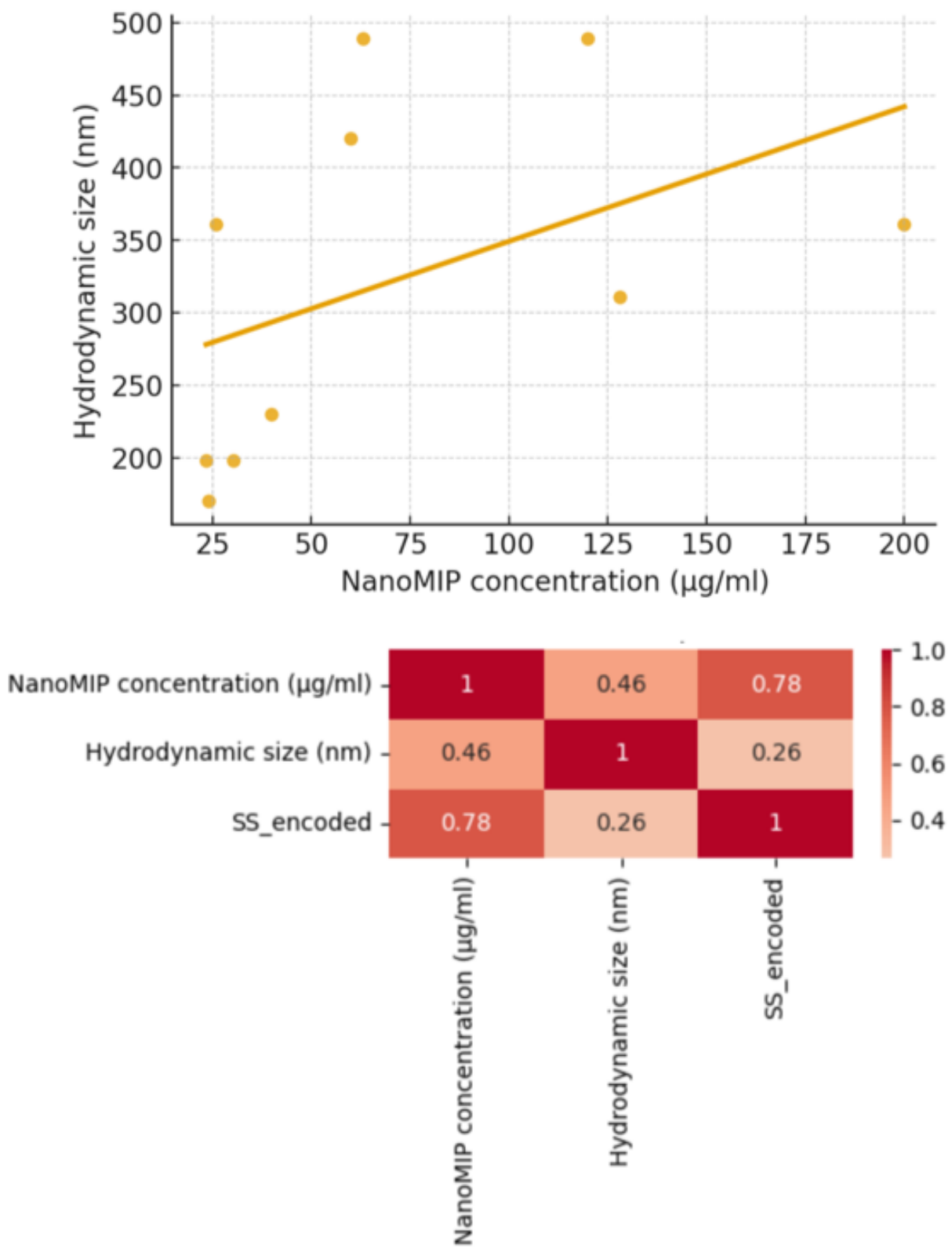

Figure S16. NanoMIP concentrations obtained in 1 mL freeze-dried solution (Table, left), and Pearson/Spearman correlation analysis between the obtained yields and hydrodynamic sizes (right, top), and the inclusion of MD-derived SS components within epitope sequences for comparison (right, bottom). While trends between nanoMIP yield and physicochemical properties were observed, correlations involving structural descriptors were interpreted cautiously due to limited sample size and simulation constraints.

A moderate positive correlation was observed between nanoMIP yield and hydrodynamic size (Pearson $r = 0.46$, $p = 0.18$; Spearman $\rho = 0.64$, $p = 0.048$), suggesting that higher yields may be associated with larger particle sizes, but for Pearson without significance and with borderline significance for Spearman. Interestingly, a stronger association was identified between yields and the MD-derived SS motifs of the template peptides (Pearson $r = 0.78$, $p = 0.12$; Spearman $\rho = 0.90$, $p = 0.037$). While the limited dataset prevents definitive conclusions, these findings indicate that both particle hydrodynamic sizes and peptides SS propensities may influence resulting yield.

Sensitivity and specificity studies: electrochemical and SPR-assays

Prior to electrochemical assays, 10 µL the of nanoMIP solution was drop-cast onto the SPGEs and incubated overnight. To minimize non-specific interactions and ensure a uniform surface chemistry, the nanoMIP-modified SPGEs were first incubated with bovine serum albumin (5

μg/mL) and ethanolamine (1 mM) for 5 min each, with PBS washes between steps. To establish a baseline, 40 μL of PBS was then added onto the electrode and incubated for 15 min, followed by square wave voltammetry (SWV) measurements (in triplicates) with the redox marker solution. Upon this blocking and reference setup, the electrodes were further incubated with 40 μL of PBS for 15 min, after which a final reference readout was recorded.

Different analyte concentration ranges were subsequently used for (i) epitope and (ii) protein affinity/cross-reactivity studies: (i) 0.01 pg/mL, 0.1 pg/mL, 1 pg/mL, 10 pg/mL, 100 pg/mL, 1 ng/mL, 10 ng/mL, 100 ng/mL, 1 μg/mL; and (ii) 10 pg/mL, 100 pg/mL, 500 pg/mL, 5 ng/mL, 10 ng/mL, 50 ng/mL, 1 μg/mL. The selected log-spaced peptide concentration ranges account for both sub-saturation and near-plateau regimes needed for robust linear/isotherm fits. Moreover, the 1 pg/mL–1 μg/mL (fM–μM) range is typically used for the affinity assessment of epitope-imprinted nanoMIPs, while ultra-low ranges (0.01 pg/mL–1 pg/mL) test the sensor's limit of detection (LOD) without immediate site saturation. For protein binding studies, 10 pg/mLwas chosen as an early dynamic range close to the LOD obtained for all epitope affinity assays. Additionally, the 10 pg/mL–1 μg/mL range is suited for capturing linear and near-saturation regions for fitting (linear/ Langmuir), and are based on reported biomarker levels in humans. For instance, up to 50 ng/mL, the concentration range covers normal and pathological levels for CD22.[36] For CD20 detection, between 10–500 pg/mL, the range identifies with early detection, while the pathological ranges (in extracellular vesicles, EVs) were reported above 2 ng/ml and up to ~120 ng/mL.[37] Regarding the BRCAs, human BRCA2 levels from 0.5 to 2.1 ng/mL were reported in serum, correlated with different clinical stages;[38] the concentration range used in our studies covers below-normal, normal, and well-above-normal levels, while 50 ng/mL–1 μg/mL range was included for differentiating between BRCAs specific vs non-specific binding. Finally, the 1 μg/mL was also considered to account for upper-normal to low pathologic boundary for β2M biomarker, although the literature on human β2M detection range is sparse. The naturally occurring levels of β2M in healthy individuals are reported between ~0.23–0.30 μg/mL.[39]

Each sample (target analyte) was incubated for 15 min, starting from the lowest to highest concentration. After each incubation, electrode surfaces were washed with PBS. Importantly, the volume used for all incubation steps, washings, and signal readouts was maintained at 40 μL for consistency. SWV readings were recorded in triplicates, and the average was taken as readout signals per each analyte type and concentration. SWV measurements were conducted using the following settings: amplitude 0.05 V; frequency 10.0 Hz and the current ranges from 1 μA to 1 mA, E_{begin} − 0.3 V, and E_{end} 0.6 V. Finally, the relative signals, representing the sensor response with respect to the reference, were calculated according to literature.[40]

Protein affinity investigations of MM/MD and ML nanoMIPs were also performed using Biacore X100 (Cytiva, Germany), a surface plasmon resonance (SPR)-based sensing device. Two-channel SPR system was docked with a gold SPR chip modified with a self-assembled monolayer (SAM) of 11-Mercaptoundecanoic acid (MUDA). Both 1-ethyl-3-(3-dimethylaminopropyl) carbodiimide (EDC) and N-hydroxy-succinimide (NHS) were purchased from Sigma Aldrich (Steinheim, Germany). The MUDA-functionalized chip surface was activated with 0.2 M EDC and 0.05 M NHS solution for 4 min. A solution of 100 ng/mL target protein (e.g., CD20, CD22, BRCA1, and B2M) in sodium acetate buffer (10 mM, pH 5)

was injected for 5 min for covalent immobilization, followed by 4 min of 10 mM ethanolamine injection for blocking on both channels.

The apparent molar concentrations of nanoMIPs were estimated as previously described, [41] using the equation below:

$$[nanoMIPs] = \frac{6}{\pi N_A d^3 \rho} \chi$$

in which the N_A denotes for Avogadro's number, d is the hydrodynamic diameter of nanoMIPs, ρ is the polymer density and χ is the weight concentration of the polymer solution in mg mL^{-1}.

The protein immobilized chips were utilized for single cycle kinetics assay of nanoMIPs for affinity investigation. Prior to kinetics assay, nanoMIPs were sonicated, syringe filtered (0.45 µm), dried, and dispersed in running buffer (10 mM PBS with 0.05 % Tween 20). They were serially diluted to obtain nanoMIP samples with concentration of 0.3, 0.15, 0.075, 0.0375, 0.01875 nM. Channel 1 was used for testing MD-based nanoMIPs, while ML-based nanoMIPs were injected on channel 2. Starting from the lowest to highest nanoMIP concentration, each sample was injected for 9 min for association, followed by 2 min of dissociation phase. Sensorgrams were processed using reference measurements to correct for baseline drift and bulk refractive index changes. The resulting sensorgrams were evaluated using Biacore X100 software to determine the equilibrium dissociation constant (K_d) as a measure of nanoMIP affinity. The methodology presented herein, including electrochemical and SPR-based sensors development, was consistently applied using the same parameters across all nanoMIP formulations.

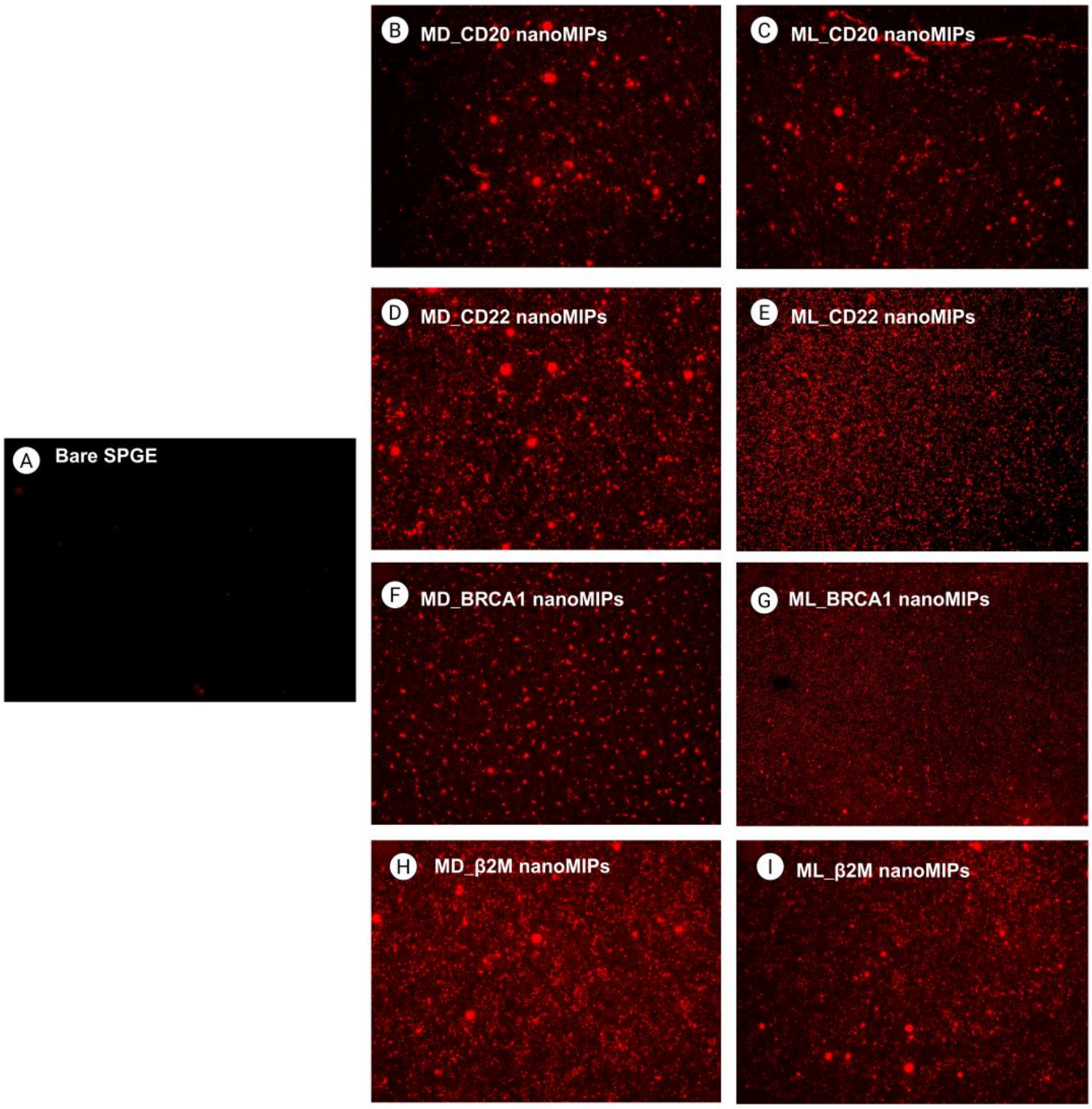

Figure S17. Fluorescence image acquisitions (10X, 56 µm scale) of bare SPGE vs electrodes used in protein rebinding studies upon electrochemical sensors development. NanoMIPs incorporating acryloxyethyl thiocarbamoyl rhodamine B monomer were used for fluorescence imaging.

Statistical analysis

All statistical analyses were conducted in Python v3.8. For the overall comparisons and correlations between nanoMIP descriptors, a dataset of 10 nanoMIP formulations (CD20, CD22, BRCA1, BRCA2, and β2M), synthesized in both MD and ML recipes, was used. Across formulations, quantitative descriptors included hydrodynamic size, PDI, ζ-potential, binding constants (K_d), heterogeneity index (m), and multiple epitope rebinding fit parameters (R^2 from linear, Langmuir, Freundlich, and Langmuir-Freundlich isotherms). Descriptive statistics (mean, variance, quartiles) were computed for each variable. Pairwise associations were examined using Pearson's correlation (linear, two-tailed) and Spearman's correlation (rank-based, non-parametric), with significance indicated at $\alpha = 0.05$. Correlations were visualized with annotated heatmaps and regression plots.

Group-level comparisons were carried out between the MD and ML subgroups and across individual recipe types. One-way ANOVA (parametric) and Kruskal-Wallis (non-parametric) were used to assess nanoMIP recipe-specific effects. For MD vs ML comparisons, Levene's test (for variance homogeneity) and Shapiro-Wilk tests (for normality) determined subsequent choices: independent *t*-tests with pooled variance (equal) or Welch's correction (unequal) for normally distributed data, and Mann-Whitney U tests otherwise. Welch's *t*-tests were also applied in SPR analyses of maximum and average RU values, hydrodynamic sizes, and ζ-potential.

The similarity between the amino acid sequences across epitopes was quantified using character-level bigram encoding (ngram_range = (2,2), analyzer = char_wb), followed by cosine similarity. Mean similarity vectors were calculated and correlated with numerical descriptors by Pearson correlation. Secondary structure annotations were one-hot encoded, enabling calculation of Pearson correlations between motif presence/absence and numeric features, as well as visualization using boxplots of each feature vs structural components. Local neighborhood structure was examined using t-SNE (perplexity = 3, Euclidean distance, random_state = 42). Multivariate modeling was performed with multiple linear regression to predict RMSE from size, ζ-potential, and heterogeneity indices, with multicollinearity assessed via variance inflation factors.

References

[1] M. Kihara, E. Chatani, K. Iwata, K. Yamamoto, T. Matsuura, A. Nakagawa, H. Naiki, Y. Goto, *J. Biol. Chem.* 2006, *281*, 31061–31069. https://doi.org/10.1074/jbc.M605358200.

[2] X. Liu, J. A. A. Ladias, *Biochemistry* 2013, *52*, 7618–7627. https://doi.org/10.1021/bi400714v.

[3] H. Yang, P. D. Jeffrey, J. Miller, E. Kinnucan, Y. Sun, N. H. Thomä, N. Zheng, P.-L. Chen, W.-H. Lee, N. P. Pavletich, *Science* 2002, *297*, 1837–1848. https://doi.org/10.1126/science.297.5588.1837.

[4] A. Kumar, C. Planchais, R. Fronzes, H. Mouquet, N. Reyes, *Science* 2020, *369*, 793–799. https://doi.org/10.1126/science.abb8008.

[5] J. Ereño-Orbea, T. Sicard, H. Cui, M. T. Mazhab-Jafari, S. Benlekbir, A. Guarné, J. L. Rubinstein, J.-P. Julien, *Nat. Commun.* 2017, *8*, 764. https://doi.org/10.1038/s41467-017-00836-6.

[6] R. K. Mondal, D. Sen, A. Arya, S. K. Samanta, *Sci. Rep.* 2023, *13*, 17843. https://doi.org/10.1038/s41598-023-45016-3.

[7] H.J. Dyson, P.E. Wright, 2005. Intrinsically unstructured proteins and their functions. Nat. Rev. Mol. Cell Biol. 6, 197–208. https://doi.org/10.1038/nrm1589

[8] M. Fuxreiter, 2012. Fuzziness: linking regulation to protein dynamics. Molecular BioSystems (MBS) 8, 168–177. https://doi.org/10.1039/C1MB05234A

[9] L. Schrödinger, W. DeLano, PyMOL, 2020, http://www.pymol.org/pymol.

[10] E. F. Pettersen, T. D. Goddard, C. C. Huang, G. S. Couch, D. M. Greenblatt, E. C. Meng, T. E. Ferrin, *J. Comput. Chem.* 2004, *25*, 1605–1612. https://doi.org/10.1002/jcc.20084.

[11] A. Anthopoulos, I. Cameron, *J. Comput. Chem.* 2022, *43*, 23–30. https://doi.org/10.1002/jcc.26829.

[12] M. D. Hanwell, D. E. Curtis, D. C. Lonie, T. Vandermeersch, E. Zurek, G. R. Hutchison, *J. Cheminform.* 2012, *4*, 17. https://doi.org/10.1186/1758-2946-4-17.

[13] K. Vanommeslaeghe, E. Hatcher, C. Acharya, S. Kundu, S. Zhong, J. Shim, A. D. Mackerell, *J. Comput. Chem.* 2010, *31*, 671–690. https://doi.org/10.1002/jcc.21367.

[14] J. Huang, S. Rauscher, G. Nawrocki, T. Ran, M. Feig, B. L. de Groot, H. Grubmüller, A. D. MacKerell Jr., *Nat. Methods* 2017, *14*, 71–73. https://doi.org/10.1038/nmeth.4067.

[15] R. W. Hockney, J. Eastwood, *Computer Simulation Using Particles*, Taylor & Francis, 1988.

[16] G. Bussi, D. Donadio, M. Parrinello, *J. Chem. Phys.* 2007, *126*, 014101. https://doi.org/10.1063/1.2408420.

[17] M. Parrinello, A. Rahman, *Phys. Rev. Lett.* 1980, *45*, 1196–1199. https://doi.org/10.1103/PhysRevLett.45.1196.

[18] B. Hess, H. Bekker, H. J. C. Berendsen, J. G. E. M. Fraaije, *J. Comput. Chem.* 1997, *18*, 1463–1472. https://doi.org/10.1002/jcc.540181209.

[19] L. Verlet, *Phys. Rev.* 1967, *159*, 98–103. https://doi.org/10.1103/PhysRev.159.98.

[20] U. Essmann, L. Perera, M. L. Berkowitz, T. Darden, H. Lee, L. G. Pedersen, *J. Chem. Phys.* 1995, *103*, 8577–8593.

[21] Grace Development Team, Grace – 2D plotting tool, Version 5.1.25, 2023, http://plasma-gate.weizmann.ac.il/Grace/.

[22] W. Humphrey, A. Dalke, K. Schulten, *J. Mol. Graphics* 1996, *14*, 33–38. https://doi.org/10.1016/0263-7855(96)00018-5.

[23] C. R. Harris, et al., *Nature* 2020, *585*, 357–362. https://doi.org/10.1038/s41586-020-2649-2.

[24] J. Reback, pandas-dev/pandas: pandas 1.3.0, *Zenodo* 2020. https://doi.org/10.5281/zenodo.3509134.

[25] F. Pedregosa, et al., *J. Mach. Learn. Res.* 2011, *12*, 2825–2830. https://jmlr.org/papers/volume12/pedregosa11a/pedregosa11a.pdf.

[26] J. D. Hunter, *Comput. Sci. Eng.* 2007, *9*, 90–95. https://doi.org/10.1109/mcse.2007.55.

[27] S. M. Lundberg, S. I. Lee, *Adv. Neural Inf. Process. Syst.* 2017, *30*, 4765–4774.

[28] ChemAxon, MarvinSketch 23.14, 2023, https://chemaxon.com/products/marvin.

[29] E. Akin, E. Sehit, N.S. Moldovean-Cioroianu, S. Tavaragondi, S. Slenczka, R. Süssmuth, F. Jurk, M. van Gemmeren, Z. Altintas, 2025. Atomic-Level Engineering of Synthetic Receptors for Enhanced Virus Detection and Removal. *Adv. Healthc. Mater*. 2502043.

[30] Z. Altintas, M. Gittens, A. Guerreiro, K.-A. Thompson, J. Walker, S. Piletsky, I.E. Tothill, 2015a. Detection of Waterborne Viruses Using High Affinity Molecularly Imprinted Polymers. *Anal. Chem*. 87, 6801–6807.

[31] Z. Altintas, J. Pocock, K.-A. Thompson, I.E. Tothill, 2015b. Comparative investigations for adenovirus recognition and quantification: Plastic or natural antibodies? *Biosens. Bioelectron*. 74, 996–1004.

[32] E. Sehit, G. Yao, G. Battocchio, R. Radfar, J. Trimpert, M.A. Mroginski, R. Süssmuth, Z. Altintas, 2024. Computationally Designed Epitope-Mediated Imprinted Polymers versus Conventional Epitope Imprints for the Detection of Human Adenovirus in Water and Human Serum Samples. *ACS Sensors*.

[33] Z. Altintas, 2018. Surface plasmon resonance-based sensor for the detection of glycopeptide antibiotics in milk using rationally designed nanoMIPs. *Sci. Rep*. 8, 1–12. https://doi.org/10.1038/s41598-018-29585-2.

[34] C. Cáceres, E. Moczko, I. Basozabal, A. Guerreiro, S. Piletsky, 2021. Molecularly Imprinted Nanoparticles (NanoMIPs) Selective for Proteins: Optimization of a Protocol for Solid-Phase Synthesis using automatic chemical reactor. *Polymers* (Basel). 13, 314.

[35] S. Choudhary, Z. Altintas, 2023. Development of a Point-of-Care SPR Sensor for the Diagnosis of Acute Myocardial Infarction. *Biosensors* 13, 229.

[36] K. Matsushita, I. Margulies, M. Onda, S. Nagata, M. Stetler-Stevenson, R. J. Kreitman, *Blood* 2008, *112*, 2272–2277. https://doi.org/10.1182/blood-2008-01-131987.

[37] L. E. Martínez, S. Lensing, D. Chang, et al., *Sci. Rep.* 2025, *15*, 25181. https://doi.org/10.1038/s41598-025-11128-1.

[38] S. Saha, P. Mandal, S. Ganguly, D. Jana, A. Ayaz, A. Banerjee, R. Chouhan, D. K. Sarkar, *Indian J. Surg. Oncol.* 2015, *6*, 378–383. https://doi.org/10.1007/s13193-015-0449-1.

[39] H. Nasimi, J. S. Madsen, A. H. Zedan, et al., *Sci. Rep.* 2023, *13*, 10658. https://doi.org/10.1038/s41598-023-37886-4.

[40] E. Sehit, J. Drzazgowska, D. Buchenau, C. Yesildag, M. Lensen, Z. Altintas, *Biosens. Bioelectron.* 2020, 112432. https://doi.org/10.1016/j.bios.2020.112432.

[41] Y. Hoshino, T. Kodama, Y. Okahata, K.J. Shea, 2008. Peptide imprinted polymer nanoparticles: A plastic antibody. *J. Am. Chem. Soc*. 130, 15242–15243. https://doi.org/10.1021/ja8062875.
